# The Epc-N domain: a predicted protein-protein interaction domain found in select chromatin associated proteins

**DOI:** 10.1186/1471-2164-7-6

**Published:** 2006-01-16

**Authors:** Jason Perry

**Affiliations:** 1 Division of Biological Sciences, University of California at San Diego, La Jolla, USA

## Abstract

**Background:**

An underlying tenet of the epigenetic code hypothesis is the existence of protein domains that can recognize various chromatin structures. To date, two major candidates have emerged: (*i*) the bromodomain, which can recognize certain acetylation marks and (*ii*) the chromodomain, which can recognize certain methylation marks.

**Results:**

The Epc-N (Enhancer of Polycomb-N-terminus) domain is formally defined herein. This domain is conserved across eukaryotes and is predicted to form a right-handed orthogonal four-helix bundle with extended strands at both termini. The types of amino acid residues that define the Epc-N domain suggest a role in mediating protein-protein interactions, possibly specifically in the context of chromatin binding, and the types of proteins in which it is found (known components of histone acetyltransferase complexes) strongly suggest a role in epigenetic structure formation and/or recognition. There appear to be two major Epc-N protein families that can be divided into four unique protein subfamilies. Two of these subfamilies (I and II) may be related to one another in that subfamily I can be viewed as a plant-specific expansion of subfamily II. The other two subfamilies (III and IV) appear to be related to one another by duplication events in a primordial fungal-metazoan-mycetozoan ancestor. Subfamilies III and IV are further defined by the presence of an evolutionarily conserved five-center-zinc-binding motif in the loop connecting the second and third helices of the four-helix bundle. This motif appears to consist of a PHD followed by a mononuclear Zn knuckle, followed by a PHD-like derivative, and will thus be referred to as the PZPM. All non-Epc-N proteins studied thus far that contain the PZPM have been implicated in histone methylation and/or gene silencing. In addition, an unusual phyletic distribution of Epc-N-containing proteins is observed.

**Conclusion:**

The data suggest that the Epc-N domain is a protein-protein interaction module found in chromatin associated proteins. It is possible that the Epc-N domain serves as a direct link between histone acetylation and methylation statuses. The unusual phyletic distribution of Epc-N-containing proteins may provide a conduit for future insight into how different organisms form, perceive and respond to epigenetic information.

## Background

Cellular DNA is packaged as chromatin, a condensed fiber composed of nucleosome core particles. Each core particle comprises 147 base pairs of DNA wrapped nearly twice around an octomer of histone proteins, which is canonically defined by an H3/H4 tetramer flanked by two H2A/H2B heterodimers [1].

The amino- and carboxy-terminal tails of histone proteins protrude from the core particle into the solvent, and are therefore amenable to post-chromatin packaging modification. Well known histone modifications include Lys acetylation, Ser/Thr phosphorylation and Lys/Arg methylation at N-terminal tails; however, examples of histone tail ubiquitinylation, SUMOylation, ADP ribosylation, glycosylation, carbonylation and biotinylation have also been described. The dynamic composite of these modifications (the epigenetic state) predicates chromatin structure, and therefore gene activity, in a manner that is not yet fully understood. It is generally thought, however, that acetylation is positively correlated with transcriptional activation and that methylation is positively correlated with gene silencing, though reciprocal examples of each paradigm have been shown (for recent reviews) [2-4].

There are two competing, but not necessarily mutually exclusive, models for how a cell interprets epigenetic information. The first is the "histone code" model which states that histone tail modification, occurring in sequential, interdependent layers, specifically alters the affinity for various chromatin associated proteins in a way that influences downstream function. For example, the histone code is thought to underlie the determination of bulk chromatin properties such as the formation and maintenance of heterochromatic and euchromatic domains [5-7]. A second hypothesis likens histone modification to commonly known receptor mediated signal transduction networks [8]. This "signaling network" model more easily accounts for the apparent degeneracy amongst certain histone modifications, and suggests that such modifications serve to confer bistability, robustness and adaptability to a presumed chromatin based network.

Regardless of operative model, certain proteins must be able to recognize particular chromatin structures or outputs, and capacitating motifs have evolved apparently for this purpose. Two well-characterized examples are the bromodomain and the chromodomain. The bromodomain, first identified in the *Drosophila *chromatin remodeling protein Brahma, is a left-handed four-helix bundle that binds selectively to acetyl-lysine [9,10]. It is conserved amongst eukaryotes, and has been found distributed into three major protein families: (*i*) ATP-dependent chromatin remodeling factors, (*ii*) histone acetyltransferases (HATs, e.g., GCN5, PCAF, TAF_II_250) and (*iii*) BET (bromodomain + ET domain) transcriptional regulators. Bromodomains can occur as a single copy or in duplicate, and when they occur in tandem, as in TAF_II_250, they can bind selectively to diacetylated histone tails with appropriately spaced acetyl-lysine moieties [11]. By contrast, chromodomains comprise histone methylation mark recognizing motifs defined by three antiparallel β-strands reinforced with a single cross-strand helix [12]. Chromodomains are also conserved across eukaryotes, and have even been found in two *Phycodnaviridae *viruses. Like the bromodomain, the chromodomain has been distributed into three major protein families, (*i*) proteins with an amino-terminal chromodomain followed by a chromo-shadow domain [Su(var)205], (*ii*) proteins with a single chromodomain in conjunction with other non-related domains (e.g., Polycomb and the *S. cerevisiae *histone acetyltransferase Esa1) and (*iii*) proteins with tandem chromodomains (CHD1), all of which participate in epigenetic events.

The Epc-N (Enhancer of the Polycomb-N-terminus) domain is defined and described below. The core of this domain is predicted to be a right-handed orthogonal four-helix bundle. It is further defined by the presence of β-strand extensions at both termini, and its conserved, and therefore PSI-BLAST defining, residues suggest a role in protein-protein interaction surface formation, possibly in the context of chromatin binding. The Epc-N domain occurs across eukaryotes, and two distinct Epc-N-containing protein families have been identified. Each of these families is composed of two subfamilies. Members of three of the four identified subfamilies have already been evidenced to participate in epigenetic events, most notably as components of histone acetyltransferase (HAT) complexes. Two of the four subfamilies (III and IV) are further defined by the presence of a five-center-zinc-binding-motif in the loop that connects the second and third helices of the four-helix bundle. This motif is composed of a PHD followed by a mononuclear Zn-knuckle, followed by a PHD-like derivative, which will be referred to as the PZPM (PHD/Zn-knuckle/PHD motif). The PZPM is also an evolutionarily conserved translocatable module, and all proteins studied to date that contain this motif have been implicated in histone methylation and/or gene silencing. Therefore, the Epc-N domain emerges as a candidate to be another building block in the limited repertoire of domains that could have affinity for specific epigenetic signatures, and a peculiar phyletic distribution of the four protein families in which it is found seems to reflect significant discrepancies in how different organisms have evolved to form and interpret epigenetic information.

## Results

### Epc-N domain discovery and annotation

The hint of a conserved sequence common to some of the proteins described below was first noted by Stankunas *et *al. when they cloned the *Enhancer of Polycomb *E(Pc) gene from *Drosophila *[13]. Later bioinformatic analyses by Koonin and colleagues also suggested the possibility of conserved sequences common to E(Pc) and Lin-49 during their seminal work on domain accretion [14,15]. At that time, fewer sequences were available, and it appeared that there could be two potentially independent modules, coined EP1 and EP2 [14,15]. However, because of the less complete repertoire of available sequences at that time, these domains could not be precisely defined. With many more sequences in hand, it is now clear that EP1 and EP2 always co-occur to form a single domain, which will be referred to simply as Epc-N (below).

**Figure 1:**
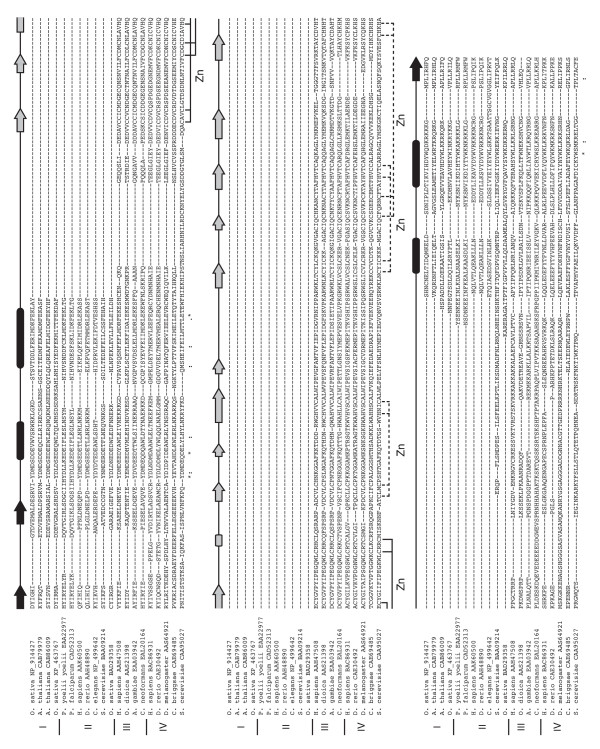
An alignment of the Epc-N domains from representative sequences of each of the four identified Epc-N protein subfamilies (I-IV). The alignment was constructed as described in the text. Secondary structure is defined by arrows (β-type extended strands) and bars (α-helices) and is annotated based on results from Jpred and corroborated by various prediction algorithms at the Polish metaserver [52]. The presumed ligands (20 total) of the PZPM found as part of the Epc-N domain in subfamily III and IV proteins are as shown.

Here, the Epc-N domain was initially recognized during an analysis of a family of plant proteins containing an unusual Agenet domain derivative (Perry and Kleckner, unpublished data). Pfam [16] and SMART [17] database searches returned no additional domain architecture information for one of the members of this family, [ref | NP_914427] from the rice *Oryza sativa*; however, a simple BlastP analysis identified a segment of sequence ~100 amino acid residues in length that was conserved amongst a group of otherwise seemingly unrelated sequences, including some from metazoans [18,19]. Using this segment as a query, a PSI-BLAST search of the non-redundant protein database (inclusion threshold E = 0.005) converges after the eighth iteration and retrieves over 300 sequences [20]. None of the above threshold hits appeared to be false positive identifications, and a few additional candidate sequences were extracted by manual inspection of the below threshold sequences retrieved by the search. The domain was delimited by the presence of divergent low complexity loops at either end, that in some instances led either to previously identified domains or to the N-terminus of the protein. Two major protein families, that could be split into four distinct protein subfamilies (I-IV) could be identified (below), some of which had additional features proximal to the Epc-N domain that appeared conserved amongst subfamily members, but not amongst all members of the superfamily. The core elements conserved across the superfamily are shown in a manually constructed [with consideration of all PSI-BLAST pairwise alignments and Jpred [21] predicted secondary structure] composite of ClustalW and T-coffee alignments of 20 representative sequences (Figure 1) [22,23]. A current list of Epc-N domain containing sequences, complete with Pfam annotation statistics for the previously identified co-occurring domains is available [see Additional file 1]. The Jpred secondary structure consensus of the Epc-N core suggests that it comprises two short extended strands, followed by four helices, and finally, an additional extended strand. Two of the four subfamilies (III and IV) are further characterized by the presence of the predicted PZPM in the loop connecting the second and third helices (below). Additional immediately obvious conserved features include several aromatic amino acid residues distributed throughout the domain (in a linear sequence sense), plus an acidic region at the N-terminus of the first helix and a basic region at the C-terminus of the fourth helix.

### The five-center-zinc-binding motif of subfamily III and IV Epc-N domains

**Figure 2:**
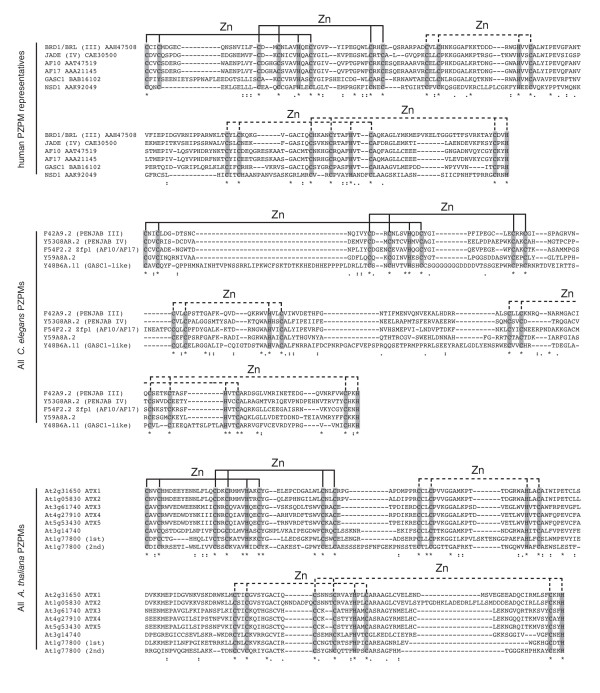
PZPMs in humans, *C. elegans *and *A. thaliana*. The alignments were constructed by T-coffee and refined manually as necessary. All PZPMs found in *C. elegans *and *A. thaliana *are shown. For human sequences, at least one representative of each identified PZPM family is shown. There are multiple homologs/paralogs in each family except NSD1 that have been omitted for clarity (3 BRD1-like molecules, 3 Jade-like molecules, 3 AF10-like molecules, 2 GASC1-like molecules). Human sequences are given with GenBank accession numbers, *C. elegans *sequences with wormbase accession numbers, and *A. thaliana *sequences with chromosomal addresses. All identifiers can be used as queries at [30]. The first portion of the PZPM (solid ligand lines) is identified by Pfam as a PHD. The remainder of the PZPM (dashed ligand lines) is not identified by Pfam but appears to comprise a previously undefined C2HC zinc knuckle or ribbon followed by a PHD-like derivative. It should be noted that the entire domain is conserved amongst all PZPM proteins, and for functional considerations it should not be viewed as simply a PHD.

As noted above, Epc-N subfamilies III and IV are further defined by the presence of a predicted PZPM (PHD-Zn knuckle-PHD-like motif) within the loop connecting the second and third helices of the canonical domain (Figures 1 and 2). As suggested, the first portion of this motif is a statistically verifiable PHD (plant homeodomain) (Pfam E ≤ 1.4e^-06 ^in all cases). However, manual inspection of an alignment of a number of subfamily III and IV sequences indicated the presence of additional conserved, potentially zinc binding residues. Since zinc binding motifs are defined by coordinating residue type and position but typically not by specifics in connecting sequence, they can be difficult to define by algorithm. Also, the canonical PHD at the beginning of the motif will not allow a PSI-BLAST search to converge with the entire domain. Therefore, a conservative approach was taken to identify which proteins in humans, the nematode *Caenorhabditis elegans *and model plant *Arabidopsis thaliana *contained the entire PZPM.

A PSI-BLAST search (E = 0.001) with the PZPM of human BRD1 [gb | AAH47508] was restricted to the first iteration of human sequences and returned 333 candidates that were analyzed manually for the presence of the entire domain. An alignment with a representative from each protein family is shown in Figure 2A. The alignment suggests that the entire PZPM comprises the verified PHD, followed by a C2HC knuckle/ribbon not described previously, which is followed by a PHD-like derivative. It should be noted that the PHD-like derivative is not identified by algorithm analysis of any sequence as a statistically significant PHD, and that the eighth ligand is an evolutionarily conserved and motif-defining histidine residue. Five groups of proteins in humans were found to contain the PZPM: (*i*) members of Epc-N subfamily III, (*ii*) members of Epc-N subfamily IV, (iii) mixed lineage leukemia (MLL) proteins (trithorax homologs) including AF10, AF17 and MLLT6, (*iv*) Jumonji transcription factors including GASC-1 and (*v*) NSD1, the nuclear receptor binding SET [Su(var), Enhancer of zeste, Trithorax] domain protein.

**Figure 3:**
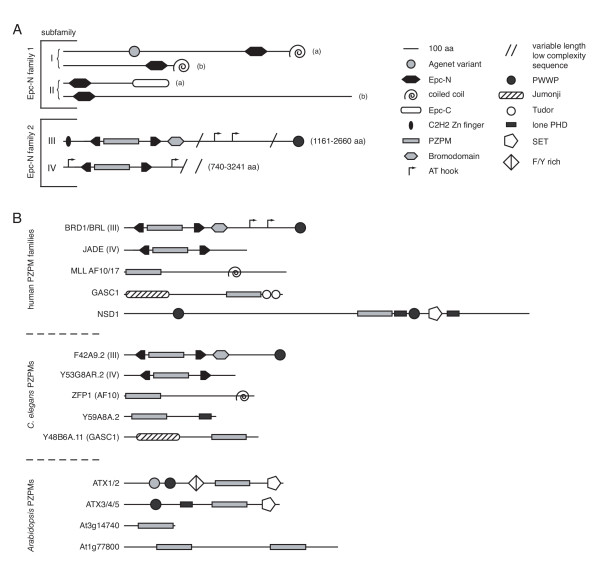
Domain architecture diagrams. (a) Domain architecture of Epc-N-containing proteins. (b) Domain architecture of PZPM-containing proteins. All diagrams are approximately to scale.

An analogous search restricted to *C. elegans *sequences was performed using the PZPM of F42A9.2 [gb | AAB03164] (the *C. elegans *Epc-N subfamily III protein) as the query. Proteins similar to those in humans were found in this organism with the exception that NSD1 is not found but instead there is a potentially related protein with simpler domain architecture (below) (Figure 2B). Interestingly, the PZPM is retained in higher plants, but not as part of an Epc-N domain. The same human BRD1 PZPM sequence fragment used above was presented as a query to the *Arabidopsis *genome WU-BLAST server (E = 0.001), which returned only Trithorax homologs and two other predicted proteins of unknown function (Figures 2C and 3B).

It should be noted that the entire PZPM is conserved across all Epc-N subfamily III and IV proteins, as well as in the other described proteins. Therefore, for functional considerations, it should not be viewed as simply a PHD, but as a single, large, evolutionarily translocatable unit that happens to exist as a subdomain in Epc-N subfamily III and IV proteins.

### Domain architectures of Epc-N and PZPM containing proteins

As alluded to above, Epc-N containing proteins can be divided into two major families (based on the presence or absence of the PZPM), each with two subfamilies. Domain architecture diagrams of the four identified Epc-N domain containing protein subfamilies are shown in Figure 3A. All Epc-N domain containing proteins are predicted by PSORT [24] to be nuclear, most contain known chromatin-associated domains, and they range in length from ~400 ([emb | CAB96695] from *P. vivax*) to over 3200 ([gb | AAS64921], RHINOCEROS, from *D. melanogaster*) amino acid residues [25]. Epc-N subfamily I proteins are characterized by C-terminal Epc-N domain followed by a coiled-coil domain at the extreme C-terminus of the polypeptide. Subfamily I can be further subdivided into two groups based on the presence (a) or absence (b) of the aforementioned Agenet domain derivative closer to the N-terminus. Subfamily II proteins are characterized by an N-terminal Epc-N domain and a C-terminal Epc-C (Enhancer of the polycomb C-terminal) domain. This subfamily can also be subdivided into two groups (a) and (b); however, to date a (b) protein has only been found in *Drosophila *(below). Though they have distinct domain architectures, subfamily I can be viewed as a plant lineage-specific expansion of subfamily II.

As described above, Epc-N subfamilies III and IV are defined by the presence of a PZPM between the second and third helices of the canonical Epc-N domain. Subfamilies III and IV are related in as much as the N-terminal positioning of the PZPM-containing Epc-N domain, and the periodic occurrence of AT-hook motifs. These subfamilies likely arose from duplication events in a primordial fungal-metazoan-mycetozoan ancestor. The difference between these subfamilies is that subfamily III members are further defined by the presence of a bromodomain (an acetyl-lysine binding four helix bundle) adjacent and C-terminal to the Epc-N domain followed by a low complexity region leading up to a PWWP domain at the C-terminus. In addition, there is a C2H2 zinc finger at the N-terminus of select subfamily III proteins (below). By contrast, subfamily IV proteins contain only the PZPM-Epc-N domain followed by a variable length stretch of low complexity sequence.

Domain architecture diagrams of PZPM-containing proteins are shown in Figure 3B. As defined above, there are five groups of PZPM proteins in humans: (*i*) members of Epc-N subfamily III, (*ii*) members of Epc-N subfamily IV, (*iii*) various MLL proteins, (*iv*) various Jumonji transcription factors, and (*v*) NSD1. Groups (*i*) and (*ii*) are as described above. Group (*iii*) members consist of an N-terminal PZPM and a C-terminal coiled-coil, while group (*iv*) members are defined by an N-terminal Jumonji domain with C-terminal PZPM followed immediately by two copies of a Tudor domain. Finally, NSD1 (*v*) is a large protein with an N-terminal PWWP domain followed by a large undefined stretch of sequence leading up to a PZPM, a lone PHD, a second PWWP domain, a SET domain and then a second lone PHD that all occur in rapid succession.

*C. elegans *PZPM proteins are very similar to those found in humans, and the respective genes are likely orthologs. There are Epc-N subfamily III and IV representatives, an apparent MLL-AF10/AF17 homolog, and an N-terminal Jumonji domain transcription factor. The major differences between *C. elegans *and humans at this level are that: (*i*) humans have multiple copies, potentially paralogs, of each PZPM-encoding gene except NSD1 whereas *C. elegans *has retained only one copy of each gene and (*ii*) that there is no apparent NSD1 in *C. elegans *but rather a unique protein (Y59A8A.2) consisting of an N-terminal PZPM and a C-terminal PHD. Since NSD1 contains lone PHD motifs in addition to its PZPM, it is possible that Y59A8A.2 and NSD1 modulate similar functions and that they are in fact encoded by orthologous genes.

As noted above and discussed below, the PZPM is conserved in plants, but not as part of an Epc-N domain. The *Arabidopsis *genome is completely sequenced, and seven genes were found to encode for proteins with the PZPM. Five were trithorax homologs, and two types of domain architectures were apparent in this group. ATX1 and ATX2 are defined by an Agenet variant (interestingly, the same variant as that found in Epc-N subfamily 1a proteins), followed by a PWWP domain, a phenylalanine-tyrosine rich domain, the PZPM and finally a C-terminal SET domain. ATX3, ATX4 and ATX5 are somewhat different and are defined by a PWWP domain followed by a lone PHD, the PZPM and the C-terminal SET domain. The other two PZPM proteins in *Arabidopsis *feature the PZPM as a stand-alone motif, one with a single copy and one where it has been duplicated.

### Phyletic distribution of Epc-N domain containing protein subfamilies

**Figure 4:**
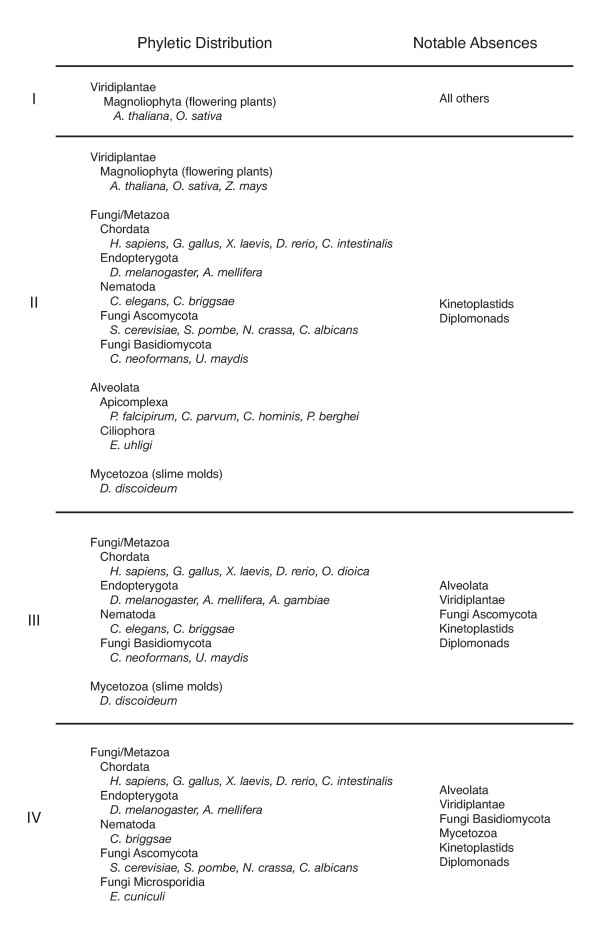
Phyletic distribution of the Epc-N domain. This domain is only found in eukaryotes with PSI-BLAST.

On analysis of Epc-N proteins at the subfamily level, and an intriguing species distribution is immediately evident (Figure 4). Subfamily I is exclusive to plants, subfamily II is almost universally conserved across eukaryotes (with the notable exceptions of kinetoplastid and Diplomonad parasites) and subfamilies III and IV, members of which contain the PZPM, are found in Fungi/Metazoa and Mycetozoa (Subfamily III only) but are conspicuously absent in plants and Aveolata parasites. Several other unusual features are observed: (*i*) there was an inferred evolutionary event at the Fungi Ascomycota-Fungi Basidiomycota split in which the primordial basidiomycote retained a member of subfamily III but not subfamily IV, and the primordial ascomycote retained a member of subfamily IV but not subfamily III, (*ii*) as described above, the PZPM found in Epc-N subfamilies III and IV persists in plants, but not as part of a Epc-N domain, and (*iii*) the domain architecture of the subfamily III protein from the chordate plankton component *O. dioica *is more similar to those of Endopterygota (all have retained the N-terminal C2H2 Zn finger) than it is other Chordata (which have not). Observation (*iii*) therefore presents an interesting example of a lower chordate serving as an evolutionary intermediate between vertebrates and invertebrates not only at the anatomical level but also at the level of protein structure/domain architecture.

### Data mining for the biological roles of Epc-N proteins

Data has been extracted from cited literature and from publicly available genomic databases including those linked to the following websites: [26-30]

**Figure 5:**
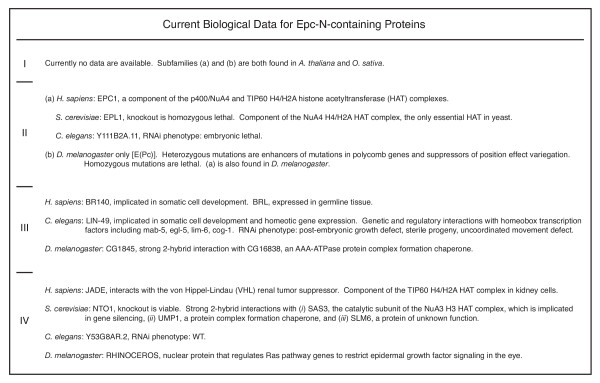
Currently available biological data regarding Epc-N-containing proteins.

Epc-N proteins appear to exist as components of large protein complexes, typically, histone acetyltransferase (HAT) complexes (Figure 5). There are not yet any data regarding subfamily I proteins; however, subfamily II proteins have been found to be essential in several organisms including budding yeast, *Drosophila *and *C. elegans*, consistent with their near omnipresence in the preceding phyletic distribution analysis. The Epc-N subfamily II protein of *S. cerevisiae*, ELP1, has been shown to be a component of the NuA4 H4/H2A HAT, the only essential HAT in that organism [31]. The homologous *H. sapiens *protein, EPC1, has also been found in similar complexes (p400/NuA4 and TIP60) in human cell lines [32,33]. In an apparent functional paradox, heterozygous mutations in a *Drosophila *subfamily II gene [E(Pc)] are suppressors of position effect variegation [Su(var)] and enhancers of mutations in polycomb genes, and EPC1 has recently been found in an additional complex, with Ezh2 (Enhancer of zeste, a SET domain protein) [13,34,35]. Thus, Epc-N subfamily II proteins are appear to be directly implicated in basic, essential epigenetic events that govern both transcriptional activation and repression.

Epc-N subfamily III proteins are immediately connected to some level of epigenetic regulation by the presence of their family-defining bromodomains. With respect to subfamilies II and IV, somewhat less is currently known about subfamily III proteins, but it is known that in humans one subfamily III protein, BR140, is involved in somatic cell development while a presumed paralog, BRL, is most highly expressed in germline tissues [36]. The subfamily III protein in *C. elegans *is LIN-49. Like BR140, LIN-49 has been shown to be involved in somatic cell development, specifically through the regulation of homeotic gene expression [37]. The RNAi knockdown line of LIN-49 shows post-embryonic growth defects, sterile progeny and uncoordinated movement defects, phenotypes which are also observed in a naturally occurring mutant in which the sixteenth ligand (20 ligands total) of the PZPM has been changed from Cys to Ser, presumably disrupting the motif [38]. Finally, the subfamily III protein of *Drosophila*, CG1845, was shown to have a 2-hybrid interaction with CG16838, an AAA-ATPase thought to function as a chaperone in protein complex assembly-dissolution. Interestingly, the subfamily IV protein in *S. cerevisiae*, NTO1, also has a two-hybrid interaction with a protein complex assembly chaperone, suggesting that this is a *bona-fide *property of HAT complex regulation.

As suggested, there is a bit more information available regarding Epc-N subfamily IV proteins. Subfamily IV proteins do not appear to be essential, and are thus inferred to take on more specialized roles than subfamily II proteins, whose roles seem to be more basic. The NTO1 knockout is viable, and the RNAi of the subfamily IV transcript in *C. elegans *has a WT phenotype. Subfamily IV proteins have been found associated with two different HATs. The human protein, JADE (of which there is three isoforms), interacts physically with the von Hippel-Lindau tumor suppressor and the H4/H2A HAT TIP60 in kidney tissue [39,40]. By contrast, NTO1 has a two-hybrid interaction with SAS3, the catalytic subunit of the NuA3 H3 HAT complex, which is involved in gene silencing. NTO1 also has two-hybrid interactions with the protein complex assembly chaperone UMP1 and SLM6, a protein of unknown function. Finally, the subfamily IV protein of *Drosophila*, RHINOCEROS, regulates Ras pathway genes to restrict epidermal growth factor signaling in the eye, but its molecular mechanism is currently unknown [41].

### Data mining for the biological roles of non-Epc-N PZPM proteins

**Figure 6:**
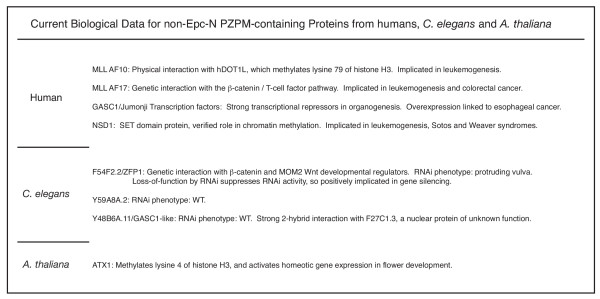
Currently available biological data regarding non-Epc-N PZPM-containing proteins.

All currently available data regarding non-Epc-N PZPM proteins point to a role in histone methylation and gene silencing (Figure 6). Consistent with this, all human PZPM encoding genes, including JADE, have emerged as proto-oncogenes with the exception of BR140 and BRL, for which there are no data on the subject [42-50]. The human non-Epc-N PZPM proteins each take on different but related roles: (*i*) AF10 has recently been shown to interact physically with DOT1L36, which methylates lysine 79 of histone H3, (*ii*) AF17 has a genetic interaction with the β-catenin-TCF pathway, (*iii*) GASC1 and other Jumonji transcription factors are robust transcriptional repressors in organogenesis, and (*iv*) NSD1 is a SET domain protein that methylates lysine 36 of histone H3 and lysine 20 of histone H4. In *C. elegans*, the homolog of AF10/AF17, ZFP1, also has a genetic interaction with the β-catenin/Wnt developmental pathway, and its loss of function by RNAi suppresses RNAi activity, strongly indicating a WT role in gene silencing. Less is known about the other *C. elegans *genes, Y59A8A.2 and Y48B6A, but they have WT RNAi phenotypes, similar to what is observed in the Epc-N subfamily IV RNAi knockdown line. Finally, in *Arabidopsis*, only one PZPM encoding gene, ATX1, has been studied thus far. ATX1 methylates lysine 4 of histone H3 and activates homeotic gene expression in flower development [51]. In sum, non-Epc-N PZPM containing proteins are clearly involved in homeotic gene expression and local and global transcriptional repression at least sometimes as a direct consequence of histone methylation. To date, no other functions have been assigned to PZPM proteins.

### Structural considerations

**Figure 7:**
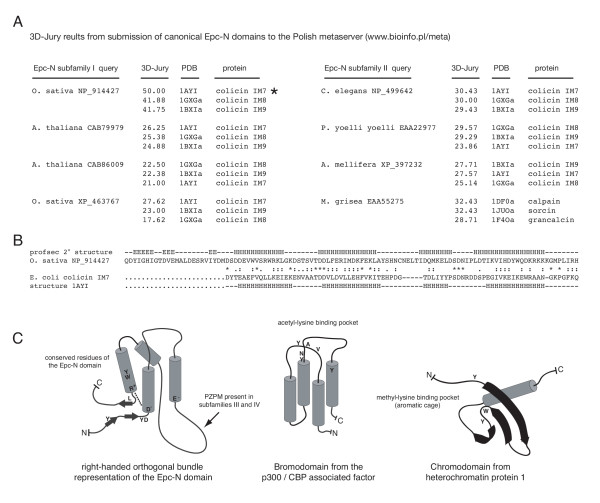
Structural classification of the Epc-N domain. (a) Results from submitting canonical Epc-N domain sequences (those lacking the PZPM) to the Polish metaserver [52] for tertiary structure analysis. A 3D-Jury score of 50 or greater is considered a significant match. The top three matches for the highest scoring sequences are shown. The subfamily I Epc-N domain from *O. sativa *NP_914427 produced a significant match to the colicin IM7 protein, a right-handed orthogonal four-helix bundle. Note that while considered statistically below threshold, all of the other top three candidates from the highest scoring sequences were matched to right-handed four-helix bundles. (b) A metaserver-generated alignment between the Epc-N domain of NP_914427 and the matched colicin IM7 structure PDB 1AYI. (c) Structural diagrams.

Jpred secondary structure analyses strongly indicated that canonical Epc-N domains (those from subfamilies I and II, that lack the PZPM) comprise four-helix all-alpha folds (above). Four-helix all-alpha folds typically form bundles, which may be right-handed or left-handed, and may have either an orthogonal or up-and-down helix-to-helix spatial orientation. A commonly known right-handed orthogonal four-helix bundle is the E/F hand, such as those found in calmodulin. A commonly known left-handed up-and-down bundle is the bromodomain, which, as alluded to above, is found in a whole host of chromatin-associated proteins, including Epc-N subfamily III members. In order to glean some evidence of what type of bundle the Epc-N domain might form, canonical Epc-N domains (those from subfamilies I and II) were submitted to the Polish metaserver [52] for tertiary structure homology analysis. The metaserver simultaneously submits a query sequence to an entire battery of threading and three-dimensional profile search algorithms (e.g., 3D-PSSM [53] mGenTHREADER [54], FUGUE [55], SUPERFAMILY [56] etc.) and then the program 3D-Jury adjudicates between conflicting results [57]. All family I and 20 family II Epc-N domains were applied to the metaserver, and the three highest scoring matches for the best fit sequences are shown in Figure 7A. A 3D-Jury score of 50 or greater is considered significant, and while one does not expect a high scoring hit with a newly defined domain for which no structures are available, one Epc-N domain sequence ([ref | NP_914427], from the rice *O. sativa*) did produce a match scored at exactly 50.00 to the colicin immunity protein (IM) 7 from *E. coli *(Figure 7B). Colicin IM proteins form right-handed orthogonal four-helix bundles and are characterized by a relatively short third helix [58]. All of the highest scoring Epc-N domains were matched to colicin IM proteins with the exception of the Epc-N domain from the family II protein of the ascomycote *M. grisea*, which found lower-scoring matches to human E/F hand proteins. As stated above, E/F hands are also right-handed orthogonal four-helix bundles. Also, like other known right-handed four-helix bundles, the third helix of the Epc-N domain is the shortest in all sequences examined. Finally, there were no matches (including sub-significant hits) to the bromodomain (a prominent left-handed four-helix bundle in the PBD) or any other left-handed bundle for any of the sequences examined. Therefore, while the evidence is not strong enough to claim a direct relationship between the Epc-N domain and colicin IM proteins, the available data do suggest that the core of the domain is likely a right-handed orthogonal four-helix bundle. The rest of the domain comprises beta structures at either end of the linear sequence that are expected to be in proximity to one another given the structure of a four-helix bundle. Of course, these computational results will need to be verified experimentally, but a diagram of a right-handed orthogonal four-helix bundle representation of the Epc-N domain is provided in Figure 7C, and compared to the bromodomain and the chromodomain. Again, the bromodomain is a left-handed up-and-down four-helix bundle that forms an acetylated chromatin-binding motif using the indicated conserved residues [10]. By contrast, the chromodomain comprises three antiparallel extended β-strands reinforced by a cross-strand helix that forms a methylated chromatin-binding motif using an aromatic cage [12]. Interestingly, strikingly similar types of residues are conserved amongst, and therefore define, Epc-N domains, suggesting that it also could form a chromatin-binding motif, but this too will need to be assessed experimentally.

## Discussion

From the sum of the analyses described above, the Epc-N domain defined here emerges as a crucial component of epigenetic regulation. The Epc-N domain appears to be a protein-protein interaction module, and can be included with the bromodomain and the chromodomain in the small cadre of potential histone code interpreters. Current evidence suggests that the core of this domain is a right-handed orthogonal four-helix bundle. Further, it can occur with a PZPM between the second and third helices, and the nature and apparent positioning of its conserved amino acid residues suggests that it may have intrinsic affinity for chromatin. Several Epc-N-containing proteins have been directly implicated as components of HAT complexes (e.g., NuA4 and TIP60 H4/H2A HATs and NuA3 H3 HAT), but intriguingly all proteins studied with PZPMs are associated with histone methylation and gene silencing. Therefore, PZPM-containing Epc-N proteins may be direct links between histone acetylation and methylation statuses.

The unusual phyletic distribution of Epc-N containing proteins likely reflects significant discrepancies in the way different organisms form, perceive and respond to epigenetic information. Most eukaryotes for which sequence information is available appear to retain at least one subfamily II gene (the notable exceptions are the kinetoplastid and Diplomonad parasites), and the available knockout lines are all homozygous lethal. Both of these observations indicate a rather fundamental role for Epc-N subfamily II proteins in epigenetic structure formation and/or recognition. However, the evolutionary peculiarities of the other three subfamilies suggest that the basic properties of the Epc-N domain can be harnessed for more specialized functions. For example, plants are strikingly different from all other eukaryotes in that they have a unique Epc-N subfamily (I, which appears to be a lineage specific expansion of subfamily II) but lack representatives of two other prominent subfamilies (III and IV). Plant cells have retained totipotency and the ability to dedifferentiate, hinting at the presence of epigenetic regulatory mechanisms different from those found in other complex multicellular organisms. Previously identified differences expected to contribute to these phenomena include a unique class of HD2-type histone deacetylases and an acetylation mark on Lys 20 of H4, which is the site of a methylation mark in animals and fungi [59,60]. The irregular distribution of the Epc-N domain proteins in plants documented here suggests that it may also have a role in plant-specific epigenetic regulation.

Epc-N subfamily III and IV members apparently participate in specialized epigenetic processes in Fungi, Metazoa and Mycetozoa. Some effects in higher organisms including those mediated by JADE, BRL and RHINOCEROS appear to be tissue specific and have localized developmental consequences [36,39-41]. Most organisms that contain one or more subfamily III proteins also have retained one or more subfamily IV proteins, so while they are similar in some sense (and likely arise from the duplication of a single primordial gene), it is doubtful that their functions are completely overlapping. This is further evidenced by the fact that RNAi of the subfamily III transcript in *C. elegans *causes severe phenotypes, while RNAi of the subfamily IV transcript does not. It is therefore interesting that Mycetozoa and Fungi Basidiomycota have retained a subfamily III gene but lack a subfamily IV gene and Fungi Ascomycota have retained a subfamily IV gene but lack a subfamily III gene. In budding yeast (an ascomycote), the subfamily IV protein NTO1 is associated with a non-essential HAT involved in gene silencing, and therefore its is likely to play a role in mating-type switching and/or telomere and rDNA maintenance. Thus, the evolutionary event described here suggests that there exists some fundamental and perhaps phylum defining difference between ascomycotes and basidiomycotes at the level of epigenetic regulation, possibly related to reproduction or telomere maintenance.

In summary, exhaustive experimental analyses of the Epc-N domain and the proteins in which it is found are anticipated to provide significant insight into both organism and tissue specific differences in epigenetic regulation and basic universal chromosomal processes.

## Conclusion

The Epc-N domain is a functionally uncharacterized globular domain found in proteins with known roles in epigenetic processes. This domain appears to be a right-handed orthogonal four-helix bundle that can accommodate a predicted five-center-zinc-binding motif (the PZPM) between its second and third helices. It is possible that this domain has intrinsic affinity for chromatin and that it links histone acetylation and methylation statuses.

## Authors' contributions

JP is the sole author of this manuscript

## Supplementary Material

Additional File 1 A collection of Epc-N proteins. Known domains are annotated with statistical significance scores from Pfam [16] analysis. Click here for file
