# Supplementary material for: The Epc-N domain: a predicted protein-protein interaction domain found in select chromatin associated proteins
Source: BMC Genomics. 2006 Jan 16;7:6. doi: 10.1186/1471-2164-7-6 (PMC1388200; doi:10.1186/1471-2164-7-6)
Supplement: Additional File 1 — A collection of Epc-N proteins. Known domains are annotated with statistical significance scores from Pfam [16] analysis. [file 1471-2164-7-6-S1.pdf]

Epc-N SUBFAMILY I

```
>OsativaNP_914427
MRSPCFLNHGGSRTDGTEVCVFLLRFLFHCAPFALRIVEIPSLGYGLSPLRLYGVFSTDLHTSRSTDRPW
EGAFDADRFYPRRRIDSAAQFGADPRVGRIRRTIQSSCVFSCEICGVVRDWRSEVVCVIRIFSAAGGGSG
LDSVVGSTLLLSGSDRPSATDATESVQGLDGEETLSERASVGAGQGPSSQNGGGKEEPSSSSSAGSKRKR
TSLSSDRVELNGLDAAGTSSGDSTWSEDSFNGRHGRSLPRNKDGHLEHSVNSGEVAVIRQPRGVLRLRKL
AQNVSIKSGTGGRKVLRSNGITKATQVQRRKRRKSQTLKENRVGSNDPINCLKVENGTCDQDNSTNFCSE
NDVSVEKQPNLSGEPSKHVHPTKESSVHVQEEDNVNLEENAARMLCSLSDNMCASSLRKSAKSLNRSSKA
YFVQHSEHFKDSCNKDKGMAGPARLLRNRDGKASSKKRRPRRHFYEVSPHDVDPFCIVKERIRVFWPLDE
IWYFGLVKEYDPMTRLHHVRYDDKDEEWINLQNERIKLLLLPSEARHKSNRRNPRSIFKPKYEVDEREDI
DRNSTGSSESGPIISLLSRSNHARSATSSNTNKQNHTHSDISPVMFDQKQSMLCSDDRPGGSPANAGEEI
PEDRTTLKDSRFRFVYSRKRSCRRKNGFLNTSEQDSDLKYRKVTGALFASVAGWGSVTENVTSRRHGILV
LSQPLKSIYKIMSEACHVWLLNALVLLHRGAMVSLWPAVHLEILLVDDTLGLKHLLLETSLRSAVSLFCL
LVGCFNSYSKASTRNESKMLCTSVRVRISGLHGRSQVVFLMFSFVGVKYPKWKHLQGQLQHHHIKRGLSK
VNCACADIKQLTNGTDQRVCTSTEHFSKGLSLDAQDSLFFTESKYSNVDPIIFCLDERSKSVQNHLDVAA
APSLLFFHHLKLRSESNLTSKSLPEFMPITLEEDQQSLPQHTSDLVHLAASASEVCSVYVSPSNTGSLDM
GTAGCINHSGSASSKLNTAKRTVSLDCNSRGIGGANITSRSFPDQIMDGSLSAVCPPQKYQKRCSSISIP
GDNISDPPDDKLLNKEEKAMQPTSDLVQELNEYPIGRVTPTAPRTPYHRNRFTSLSRTFGDGSKLWQEDI
MVTGFAGGSKKPRTQVLYSVSPRSDEPGSKHKGHFRKIQSHSSAKRLPDNSRSEQSSPESLACVANVLVT
VGDRGWREYDTQITIDSDGQSERRICVKLAEGTKYAHKVLQVLQPGATNRYTHAMIWKGGTEWCLEFPDR
SQWLIFKQMHDECYSHNIRAASVKNIPIPGVCFAEAHDDHDAVSFVRSQDYIGHIGTDVEMALDESRVIY
DMDSDDEVWVSRWRKLGKDSTSVTDDLFERIMDKFEKLAYSHNCNELTIDQMKELDSDNIPLDTIKVIHD
YWQDKRKKKGMPLIRHFQSAMWKIHEQQLQEWESTALRIQGSSNGYQVKLPPKPALFAFCLRPRGLQPQI
PYKGPKQRSHKKLMSSGSFSREQDGFYRPGYSPRFSVKTESPRAFDGLERCSTPRFLRTNSVKRTASFAF
SEDHQPSPSFRHQRVKRGAPDHWNAVIHEWQSSKHLLPGASQSPRVDIEELKQRDASSAAQHAVAMAKLK
REKAHLLMHKADLALHKATVALMMADAIKASSRDSSRDGRRDFRDD

Agenet variant
Epc-N
Coiled coil

>AthalianaCAB79979
MQVAFMENRLGNSNGVGISKKSRSLDLKTLYKSSISKDSVNKSFKRKHRSGIDGDQLKQDKKSRKVVSLS
SFKKVGSQNESILDKACNGTTILHNLEDSKEVGLDEKLCDSNGLQVISVGLASSTIYVPRRRRDFVGRSR
FENGLAQKSAGESDSQEELVVNIPKVTAEESSVQDQPSKVEEKDSDKDIKESNSAAPLQLENGHSNQSPV
KDDQLVVVKQRNSNSRKRKSSASNRRVGKEAKSSGDASGRISKVSREDDEENLEANAAIMLSSRFDPNCT
QFPSNSVTPGSPSASRLHPLPSGKNSVDPRSELLSSKCVSDDTDDRMLRPRRHNDDGKGKVRKRRHFYEI
LFSDVDSHWLLNKKIKVFWPLDERWYHGFVDGFDGDKNLHHVKYDDRDEEWINLQGERFKILLFPSEVPG
KNQRKRRCSESKSTQKVKGNDTSSKDEEKQKEKLEDDSCMESEPIITWLARSRHRDKSSTLKAVQKRKKT
DVMTSNESVKMNGDVTDRSASSLASCGLPGPSKNELESSGFRNGSIFPIVYCRRRLHTAKKDIYKESGYN
SVEFLKQFLVSKSPDPGVEFLPIEDSGDLELCCPWNESEQFELSLSLQGVSLMSYFLMADVDWLSRAALL
LRHGTLVTLWPRVRLEMIFLNNQDGLRYLIFEGCLMEVVQLIFRILMVVDHSNKQGAQGADADLQLPVFS
IGLQVSCIPGFQRQLGFQIYSFHEVKHSKWSYLEQNVRRHSLLVKQVSIAECTHNNMKVLQKVMQKRSRH
GISSGLVSRGSSSAEAWPTSVCYKKQNTSPFALLFTARPPTLLLSLHLNMIRELGHDSADFLGIERDLVT
HRGCDMADFTNEHSELSLKSKSQTDEPIITSSRAQESKDLHTPSQSQQLGSDSENWMSYSSSVVRHKHET
RSNVSVNGISIQVPISDDCEDGTPQSSNLALNIQGSSNSSPKATAPRSMWNRSKSSLNGHLSHGWSDSKG
DFLNTNLANGPKKRRTQVSYSLPSGGSDSRNKGSLLKGMPNKRIRRSTADVTKGIQKDLESSLCDANVLV
TLGDRGWREYGAQIFLEPFDNNEWRLAVKISGTTKYSHRAHQFLQPGSVNRFTHAMMWKGGKDWTLEFPD
RGQWFLFKEMHEECYNRNTRAALVRNIPIPGIRMIERDNFDGTETEFIRSSSKYFRQTETDVEMALDPSR
VMYDMDSDDEQCLLRIRECSSAENSGSCEITEDMFEKAMDMFEKASFVKQRDNFTLIEIQELTAGVGSLE
AMETIYELWRTKRQRKGMPLIRHLQPPLWEKYQRELKDWELVMSKANTPNSCGSQKKQSPTEKPAMFAFC
FKPRGLEVKHRGTKHRSQKKLSVYAQHSSALGDYDGCNSSGRRPVGFVSGDERFLYSNQSYEHSNEFSVH
PGTYSPRDLGMGYFSSGGNGYHRNHQNKSQRINGKRNTSERWDAGYSECPSSNLVCYSNGSQRPDVEGIR
NSTDIDEYKLRDAAGAARRACALAKLKRERAESLRYKADLAIQKAAAALMCAEAVKASSEDLGNNNGVES
SSEG

Agenet variant
Epc-N
Coiled coil
```

>AthalianaCAB86009
MPSVGMRRTTRVFGVVKAADGARVLRSGRRIWPNVGEPKVRRAHDVVDRDCDSVLKNQNKSKGNKVSSGK
SNSQPCSPKQVSSEKEDKVDDFPVTKRRKVRNEGVGDEKTVDKMFGIVYSRKRKRLCEPSSSDRSEEPLR
SLKFYRRRRKLSQRVSSVLTLTVDWSCEDCWFLTVFGLAMRYIRREELRLSSLASFFLSQPINQVFADHG
VRFLVRSPLSSRGVCKFFGAMSCLPLFSADFAVIPRWFMDMHFTLFVRVLPRSFFFVEKSLYLLNNPIEE
SDSESELALPEPCTPRNGVVVGLHPSVRASKLTGGNAQYRGNLGSHSFQKRRSSLRRRRARNLSHNAHKL
NNGTPVFDISGSRKNRTAAVSSKKLRSSVLSNSSPVSNGISIIPMTKTKEELDSICCSANILMIHSDRCT
REEGFSVMLEASSSKEWFLVIKKDGAIRYSHMAQRTMRPFSSNRITHATVWMGGDNWKLEFCDRQDWLGF
KDIYKECYERNLLEQSVKVIPIPGVREVCGYAEYIDNFPSFSRPPVSYISVNEDEVSRAMARSIALYDMD
SEDEEWLERQNQKMLNEEDDQYLQLQREAFELMIDGFEKYHFHSPADDLLDEKAATIGSISYLGRQEVVE
AVHDYWLKKRKQRKAPLLRIFQGHQVKKTQLLSKPVFRKRRSFKRQGSQLHGKAKQTSPWMVAVKAAEPE
EEDDILRMEEAKVLADKTMETAIAKRRRAQILAENADLAVYKAMRALRIAEAIKEAESREVDTTVV

Epc-N
Coiled coil

>OsativaXP_463767
MRKRTASDRIRVPSSNPAPSPSPPPPPPPEEPAVPMPHVGARRSTRVFVPKTPRPPQPSDPARVLRSGK
RLAFSESPADAHWFQCKPNNCFHVHDHQRLHDDPKPPPPPLPRTRSFGIVYSRKRRRRRLPEPKEDTRFA
IVFTRKRPKVAPFQHHAPNDLATIPCSSSREFASRTGFFDSHFLTLVDCIPTNKADAAMLIVLVDSSCSG
SSQHFLRLLLSVLRWMRSCRRGKVRNLASFLSSDAVATALALRGLHFVQLQCRRDCALSQRALVQCGWCE
LRGAKDSEPLLSVNFLAVPSYFQILHLLIALESMYLPAVIRTRMHLVGGAEEIYPRTLLEEDSESLSTGD
TDPAVDLCSNKLCSVAQDYVPLEEIAGVVVHGLRLKKHQRKRSSMRHPLSRQRLAARFPDKVVATNQTDV
ARQTEADAPPSVSPELPLEPVKPKAALEISLDLLENMDDSDVSTPIGSNGKQKRSSLKSPIERMNERLAL
AEVRQNIDSVHCRANLLIIQPDRCWREEGAEVMLEPSESNEWCIAVKIHGVNRISLKPSEQRFYVVVNRFT
HAYILAVDDGLKIEFSDKWDWLLFKELQIEGRERNSQGKMIPIPGVNEVSDDMGVIGTYPFSRPVPDYIR
MADDEVGRALSRDSVYDLDSEDEQWLTQLNHSDSDRKSAHLNHISYEDFEKMITTFEKDAFNNPEGTSDL
DQILSRYPTLEKDHNVLAVHEYWINKRYKKGVPLLRILQGATLRRGQLSQRSIKKKRSFKRQRSQAGRGK
PDICLQDANGAEEEALRRVVEAERAATQAGETAVRLRSRAQRLMAKAELVAYKSIMALRIAEAARISDSS
RDLVLTTLD

Epc-N
Coiled coil

Epc-N SUBFAMILY II

>RnorvegicusXP_342433
MVIPVPEAESNVNYYNRLYKGEFKQPKQFIHIQPFNLDNEQPDYDMDSEDETLLNRLNRKMEIKPLQFEI
MIDRLEKASSNQLVTLQEAKLLLNEDDYLIKAVYDYWVRKRKNCRGPSLIPQIKQEKRDGSTNNDPYVAF
RRRTEKMQTRKNRKNDEASYEKMLKLRREFSRAITILEMIKRREKTKRELLHLTLEVVEKRYHLGDYGGE
ILNEVKVNRSEKELYASPATLHNGNHHKVQECKTKHPHHLSLKEEASDVVRQKKKHPKKPKAEAVLAPPQ
PTPETVPVINKSDIKQYDFQSSDEDEFPQVPSPASEPEEENDPDGPCAFRRRAGCQYYAPRLDQANNHPC
ESSELADLDKLRYKHCLTTLTVPRRCIGFARRRIGRGGRVIMDRISTEHDPVLKQIDPEMLNGLSSSSQT
IDFSSNFSRTNASSKPCENRLSLSEILSNIRSCRLQCFQPRLLNVQDIDSEECTSRKPGQTVSSKRVSAA
SVALLNTSKNGISVTGGITEEQFQTHQQQLAQMQRQQLAQLQQKQQSQHSSQQTHPKAQGSSTSDCMSKT
LDSASAHFAASAVVSAPVPSRSEGSKEQNTGHNNMNGVVQPSGPSKTLYSTNMALSSSPGISAVQLVRTV
GHTTTNHLIPALCTSSPQTLPMNNSCLTNAVHLNNVSVVSPVNVHINTRTSAPSPTALKLATVAASMDRV
PKVTPSSAISSIARENHEPERLGLNGIAETTVAMEVT

Epc-N
EpcC 7.5e-160

>MmusculusAAH48785
NVNYYNRLYKGEFKQPKQFIHIQPFNLDNEQPDYDMDSEDETLLNRLNRKMEIKPLQFEIMIDRLEKASS
NQLVTLQEAKLLLNEDDYLIKAVYDYWVRKRKNCRGPSLIPQIKQEKRDGSTNNDPYVAFRRRTEKMQTR
KNRKNDEASYEKMLKLRREFSRAITILEMIKRREKTKRELLHLTLEVVEKRYHLGDYGGEILNEVKVNRS
EKELYASPATLHNGNHHKVQECKTKHPHHLSLKEEASDVVRQKKKYPKKPKAEAGIAPQQPTPETLPVIN
KSDIKQYDFQSSDEDEFPQVPSPASEAEEENDPDGSCAFRRRAGCQYYAPRLDQANNHMCENSELADLDK
LRYKHCLTTLTVPRRCIGFARRRIGRGGRVIMDRISTEHDPVLKQIDPEMLNGFSSSSQTIDFSSNFSRT
NASSKPCENRLSLSEILSNIRSCRLQCFQPRLLNVQDIDSEECTSRKPGQTVSSKRVSAASVALLNTSKN
GISVTGGITEEQFQTHQQQLVQMQRQQLAQLHQKQQSQHSSQQTHPKAQGSSTSDCMSKTLDSASAHFAA
SAVVSAPVPSRSEGSKEQNTGHNNMNGVVQPSGPSKTLYSTNMALSSSPGISAVQLVRTVGHTTTNHLIP
ALCTSSPQTLPMNNSCLTNAVHLNNVSVVSPVNVHINTRTSAPSPTALKLATVAASMDRVPKVTPSSAIS
SIARENHEPERLGLNGLAETTVAMEVT

Epc-N
EpcC 3.8e-157

>CfamiliarisXP_533350
MVIPVPEAESNVNYYNRLYKGEFKQPKQFIHIQPFNLDNEQPDYDMDSEDETLLNRLNRKMEIKPLQFEI
MIDRLEKASSNQLVTLQEAKLLLNEDDYLIKAVYDYWVRKRKNCRGPSLIPQIKQEKRDGSTNNDPYVAF
RRRTEKMQTRKNRKNDEASYEKMLKLRREFSRAITILEMIKRREKTKRELLHLTLEVVEKRYHLGDYGGE
ILNEVKINRSDKELYATPATLHNGNHHKVQECKTKVNILWGEAHMHPHHLSLKEEASDVVRQKKKYPKKP
KAEALITSQQPTPETLPVINKSDIKQYDFHSSDEDEFPQVPSPVSEPEEENDPDGPCAFRRRAGCQYYAP
RLDQANHSFENSELADLDKLRYRHCLTTLTVPRRCIGFARRRIGRGGRVIMDRISTEHDPVLKQIDPEML
NGFSSSSQTIDFSSNFTRTNASNKHCENRLSLSEILSNIRSCRLQCFQPRLLNLQDSDSEECTSRKSGQT
VNNKRVSAASVALLNTSKNGISVTGGITEEQFQTHQQQLVQMQRQQLAQLQQKQQSQHSSQQTHPKAQGS
STSDCMSKTLDSASAHFAASAVVSAPVPSRGEVAKEQNTGHNNINGVVQPSGTSKTLYSTNMALSSSPGI
SAVQLVRTVGHTTTNHLIPALCTSSPQTLPMNNSCLTNAVHLNNVSVVSPVNVHINTRTSAPSPTALKLA
TVAASMDRVPKVTPSSAISSIARENHEPERLGLNGIAETTVAMEVT

Epc-N
EpcC 3.1e-163

>AmelliferaXP_397232
MSKLSFRARALDASKPMPIYMAEELPDLPDYSAINRSLPQMPSGMEKEEECEHHLQRAICTGLIIPTPEV
TDLTDVEAYDKIYPADYKLPRQLIHMQPFAMEQDIPDYDMDSEDEKWVAIQSRKMDLTPLQFEEMMDRLE
KSSGQTVVTLNEAKALLKEDDDLIIAVFDYWLNKRLKTQHPLLLTVKTEHRFGSAANNPYLAFRRKTEKM
QTRKNRKNDETSYEKMLKLRRDLSRAVTLFEMVKRREKTKREHLHLTIEVYEKRYQAQDFNGQILAEVSA
LKTPRPAFAPLFTNQFGVHQNWANKVCSKDEVVPRKEKRQYKKRKHKTDSRGGSLEDNGVRSGTGSGGGR
GSRPSVLGGGLDPLISSDEDGPLPSHSHSQPSVPSDRDDEDIADEGQFAFRRNRNSTYLPPVSGGFGNWP
WCDKNEGGMADKKYRFALTSISKPVPRCIGFARRRIGRGGRVILDRCSTDMDDMWSSLDFTIHQPKREPV
DSDTTAMSTTSVKKEWLHFRPKTPPVSVHSPSEESSDTDSDIEPILTQSKPLPYPFPPEATSICVEVEPD
RAGDEPPFTSEFNIADYFSPDTLSDFDLAVASITSSACISGSDNNSNDTRTDELGSSHFVVTPLPSSLFA

QPATQQSRPQCSGSGFSVVNTSTSSIVPSSFSCTINQPTTSNAVSTQCANAAIETQSNQSKQQQHRQLPN
NSSPVSILSKSLTSPTNNRNGGSSGSGSAGVRVFSASTTTSGTITNFGNGHQNGPLSHINNSNNLSNSTH
VVNNQSTIVLPQKHPPSNLLPGLIAQSKLASLARQQQQTQSQASTQQIPTSGEITLNSNSENMDMKVDGV
ESATCDGKPQQQQLVRANKTNSLAMEVT

Epc-N

>PtroglodytesXP_526096
MTEVITAFKEFLPLTAFNLDNEQPDYDMDSEDETLLNRLNRKMEIKPLQFEIMIDRLEKASSNQLVTLQE
AKLLLNEDDYLIKAVYDYWVRKRKNCRGPSLIPQIKQEKRDGSTNNDPYVAFRRRTEKMQTRKNRKNDEA
SYEKMLKLRREFSRAITILEMIKRREKTKRELLHLTLEVVEKRYHLGDYGGEILNEVKINRSEKELYATP
ATLHNGNHHKVQECKTKVNIVWEEACMDEEASDVVRQKKKYPKKPKAEALITSQQPTPETLPVINKSDIK
QYDFHSSDEDEFPQVLSPVSEPEEENDPDGPCAFRRRAGCQYYAPRLDQANHSCENSELADLDKLRYRHC
LTTLTVPRRCIGFARRRIGRGGRVIMDRISTEHDPVLKQIDPEMLNSFSSSSQTIDFSSNFSRTNASSKH
CENRLSLSEILSNIRSCRLQCFQPRLLNLQDSDSEECTSRKPGQTVNNKRVSAASVALLNTSKNGISVTG
GITEEQFQTHQQQLVQMQRQQLAQLQQKQQSQHSSQQTHPKAQGSSTSDCMSKTLDSASAHFAASAVVSA
PVPSRSEVTKEQNTGHNNINGVVQPSGTSKTLYSTNMALSSSPGISAVQLVRTVGHTTTNHLIPALCTSS
PQTLPMNNSCLTNAVHLNNVSVVSPVNVHINTRTSAPSPTALKLATVAASMDRVPKVTPSSAISSIAREN
HEPERLGLNGIAETTVAMEVT

Epc-N
EpcC 2.7e-163

>HsapiensAAK60500
MEKEEESEHHLQRAISAQQVFREKKESMVIPVPEAESNVNYYNRLYKGEFKQPKQFIHIQPFNLDNEQPD
YDMDSEDETLLNRLNRKMEIKPLQFEIMIDRLEKASSNQLVTLQEAKLLLNEDDYLIKAVYDYWVRKRKN
CRGPSLIPQIKQEKRDGSTNNDPYVAFRRRTEKMQTRKNRKNDEASYEKMLKLRREFSRAITILEMIKRR
EKTKRELLHLTLEVVEKRYHLGDYGGEILNEVKISRSEKELYATPATLHNGNHHKVQECKTKHPHHLSLK
EEASDVVRQKKKYPKKPKAEALITSQQPTPETLPVINKSDIKQYDFHSSDEDEFPQVLSPVSEPEEENDP
DGPCAFRRRAGCQYYAPRLDQANHSCENSELADLDKLRYRHCLTTLTVPRRCIGFARRRIGRGGRVIMDR
ISTEHDPVLKQIDPEMLNSFSSSSQTIDFSSNFSRTNASSKHCENRLSLSEILSNIRSCRLQCFQPRLLN
LQDSDSEECTSRKPGQTVNNKRVSAASVALLNTSKNGISVTGGITEEQFQTHQQQLVQMQRQQLAQLQQK
QQSQHSSQQTHPKAQGSSTSDCMSKTLDSASAHFAASAVVSAPVPSRSEVAKEQNTGHNNINGVVQPSGT
SKTLYSTNMALSSSPGISAVQLVRTVGHTTTNHLIPALCTSSPQTLPMNNSCLTNAVHLNNVSVVSPVNV
HINTRTSAPSPTALKLATVAASMDRVPKVTPSSAISSIARENHEPERLGLNGIAETTVAMEVT

Epc-N
EpcC 5.6e-164

>DmelanogasterCAH70455
MEHHLQRAISAQQVYGEKRDNMVIPVPEAESNIAYYESIYPGEFKMPKQLIHIQPFSLDAEQPDYDLDSE
DEVFVNKLKKKMDICPLQFEEMIDRLEKGSGQQPVSLQEAKLLLKEDDELIREVYEYWIKKRKNCRGPSL
IPSVKQEKRDGSSTNDPYVAFRRRTEKMQTRKNRKNDEASYEKMLKLRRDLSRAVTILEMIKRREKSKRE
LLHLTLEIMEKRYNLGDYNGEIMSEVMAQRQPMKPTYAIPIIPITNSSQFKHQEAMDVKEFKVNKQDKAD
LIRPKRKYEKKPKVLPSSAAATPQQTSPAALPVFNAKDLNQYDFPSSDEEPLSQVLSGSSEAEEDNDPDG
PFAFRRKAGCQYYAPHLDQTGNWPWTSPKDGGLGDVRYRYCLTTLTVPQRCIGFARRRVGRGGRVLLDRA
HSDYDSVFHHLDLEMLSSPQHSPVNQFANTSETNTSDKSFSKDLSQILVNIKSCRWRHFRPRTPSLHDSD
NDELSCRKLYRSINRTGTAQPGTQTCSTSTQSKSSSGSAHFAFTAEQYQQHQQQLALMQKQQLAQIQQQQ
ANSNSSTNTSQGFVSKTLDSASAQFAASALVTSEQLMGFKMKDDVVLGIGVNGVLPASGVYKGLHLSSTT
PTALVHTSPSTAGSALLQPSNITQTSSSHSALSHQVTAANSATTQVLIGNNIRLTVPSSVATVNSIAPIN
ARHIPRTLSAVPSSALKLAAAANCQVSKVPSSSSVDSVPRENHESEKPALNNIADNTVAMEVT

Epc-N
EpcC 1.1e-159

>DmelanogasterAAC64271
MSKLSFRARHLDPSKQMPIYLAEELPDLPEYSAINRAVPQMPSGMEKEEESEHHLQRAICTGLIIPTPEV
LQTDQPFYDAYYPPDYKMPRQMIHMQPLGLDTEVPDYDMDSADEDWLSQQQRLELTELKFEQMMDRLEKS

SGQTVVTLNEAKSLLNQDDETSISVYDYWLNKRLKMQHPLILTVKTESRPGASSNNPYLAFRRRTEKMQT
RKNRKNDEASYEKMLKLRRDLQRATTILEMVRRREETKRDHLKMTVNIFEKRVEMRDFNGAVYSELNSQY
KNTRPAYNPLYTNQYSQGAAAVGTAGAILAALPTGLLPGGAGAAGNANVFGSASQYLNSSNLTMDAISGS
GNGNSSRKEKRPYKKRKHKLPRDKQQHQQQQTQQHQQQQYLPASGGAGVSPAHHLPHHLHHLSRQQSASP
AANDSIADSEEEDYFGAGAQNGNKLGSESEEETPFAFRRRSSCVYLPTRQRDGRYPWDSADEDMAPSGAC
SDAKYRYTLTSLNYPRPRCIGFARRRLGRGGRILLDRATTNFDDFWSQLDYTVMESVTVNKCRDKIKPGD
PDQLCKPSSPPTVVDLVPAGKPVDGEHPVPELIKEEPSSDTEKPVAATESGRESKERELEEKFENDVDDE
FVASDDENVSRLGIYSSAVTLPQSCADLRQKRRRLRRKKQQLRELNAAKRLKRSSEAVATAEDVQLEDED
TKPDLRPLPRAYLQRLALILGQKLKVEEDDNTEVSENVAPELLEMNDPEQLQPPRANHQQHHRSNHNNNN
NTVLNNNNSSSSNVMNKDVSISEKINVIKQEAEELVGGKCEDQPVASTSAAAAAARAAEAAAAAEAASST
TTLAAPNPGPSLEEVAKTIKREMIDADDSNEPLSSIRTAAVMLSTQTSVSELMDDEAEDDVNLAQLSSII
RHTAVKKELEAQQHQQQVQHEQMQAARKMEEDEAAPCLNQLPDVIRLRNIRNSQVHARPKDLFVQAPVDE
EFNADYMGGLSPTSSQRLDICNELLSEIRRDWLHFRPKTPTDELSDSQDCEVGKTCDSHAVDWTQGTPIS
VELNRLGKREAEESDSSSYFLSSGFKYRDLESDAQLLQTVQAQDYARGSSKSPNCSGSGSGSALEFNLSG
GDSLNDINNLLGCDDEDDNHEHMLDNILQECAMDDPKALNQATNFWNGILDGEAAVDEVEMADQLLDCID
EKKSKVGAADTSKRAGRNRKARLIQAPVGSSAFTVCPTAETLKERELFFSQAPPVEVPPKEKTPAEVELP
EVVVKETANLPVKVEAVEKPQSEVLPVAPQPQQLQPQAITLPTTQLINIPAQISTQKQHQQPQVTQLLNQ
FANAGPQIIARTEYGGGAAVGDIVTITPHTGSSPLPTLTAQQQLQHQLAQAQLRNVTVQQRQATPQNPNQ
NQNPQQQLLFQMQRDPSRSLTPLQAAGAGATGNHLIYTTSCGEIVTAGAAASASQKVTYAIQKAGVSSGG
ATTASIGPNTVITLSNVKVQNDDISSGGSGNSGSSVNIINTASGQQLAVHSIAGMQQQGQQQVQQVTTST
PLLVATPTRNNVQQQQQQLVQQQPIVWRQVSGTNTAVATTAVLSTTVDSSPKAVTNQILWTSRATQKRQL
NGPENTDINKLLLNRKLPRKQQIQQQHQQIQLTKQQLQQLVQHEELDDLVATSTGNSGDQMETGDGLQQQ
QQQQQQQQQLQKLSKVIKMSQFPLLVSDVSLQQQQSVSGQAQQQQLKPNSAAAIAANHNYSLHPASTAII
TAQATPQQANKIFLTSSNSGGGGSAGNFTIATASELQAAAAGQKLHYKELPNSNLKLNFVSSAGTGEQTQ
QVGATTVLLNKSQQQQQQQQHQKLKTLATTGNSQTGQGDKRVLYTSLLNVKPLQKNNSGQMQMQLGPGGL
QQVLRGRLNNSAIITRTLPLGTTVNSTGGAVGPTTTTIRQLVTTNANNVSSQDGNLLSAKEVGKALTVEQ
VIASANNGGVVLPTSNNNNNNNGSGAGGNGGPLNSGVNATGAGGGGSTGAGVGGAGVTSTINR

Epc-N

>XtropicalisAAH75105
MSKLSFRARALDAAKPLPIYRGKDMPDLNDCVSINRAVPQMPTGMEKEEESEHHLQRAISAQQVFREKKE
SMVIPVPEAESNVHYYSRLYKGEFKQPKQFIHIQPFNLDYEQPDYDMDSEDETLLNRLNRKMEIKPLQFE
IMIDRLEKASSNQLVTLQEAKLLLNEDDYLIKSVYDYWVRKRKNCRGPSLIPQVKQEKRDGSTNNDPYVA
FRRRTEKMQTRKNRKNDEVSYEKMLKLRREFSRAITILEMIKRREKTKRELLHLTLEVVEKRYNLGDFGG
EILNEIKIPKVEKEIHVIPPSLHNGNHHKVPECKVKNTHHPSVKDEVLEIVRLKKKYPKKPKAETLVTPQ
PQATSEPLAVINKSDIKQYDFQSSDDDEFPQVPSPLSELEEENDPDGSYAFRRRTGCQYYAPRLDQMNDT
PETTDLSGLARHRYRHCLTTLTVPRRCIGFARKRLGRGGRVIMDRLSTEHDSILKQIDPEMLSGFSSSSH
VAQPPSSPSRTNASDRHCENRLSLPEILSNIKSCRLQCFQPRLLHTQDSDREECTSRLGQAVNVKRVSAA
LLNTSKNGITVTGGITEEQFQTHQQQLVQMQRQQLAQLQQKQQSQQSLQQSHPKAQGSAKSDCMSKTLDS
ASALFAASAVVNSPTPGRSEVNKDQNAGHSNLNGVVQPSGTAKTLYSTNMALSSSPGISTVQLVRTVGHP
TTNHLIPTLCTSNPQTLTMGNSCLANTVHLNNVSVVSPVNVHINTRTSAPSPTALKLATVAASMDRVPKV
TPSSAISSIARENHEPERLGLNGIADTTVAMEVT

Epc-N
EpcC 4.3e-142

>GgallusXP_418573
MDIAGAVAVMAGLWLGVPWAACVLLQPVALLSAERWFPSAAVSIGVVGRTADTVMFKHRTRLLHPGFLLG
NRIRFSCESANDKILKFPLQKGALCVLLEVPDVSTGKAVREDAQDCESGGCSAAFIMSGDCMKQSCFLTA
PLGERSCCSPSALELSDLMEHHLQRAISAQQVYGEKRDNMVIPVPEAESNIAYYESIYPGEFKMPKQLIH
IQPFSLDAEQPDYDMDSEDEVFVNKLKKRMDISPLQFEEMIDRLEKGSGQQPVSLQEAKLLLKEDDELIR
EVYEYWIKKRKNCRGPSLIPAVKQEKRDGSSTNDPYVAFRRRTEKMQTRKNRKNDEASYEKMLKLRRDLS
RAVTILEMIKRREKSKRELLHLTLEIIEKRYNLGDYSGEIVSEVMAQRQPMKPTYAIPIIPVTNSSPFKH
QETMELKEYKVNKQDKSDVIRPKRKYEKKPKVLPSSAAATPQQTSPAALPVFNAKDLNQYDFPSSDEEPL
SQVLSGSSEAEEENDPDGPFAFRRKAGCQYYAPHLDQPGNWPWSSPKEGRLGDVRYRYCLTTLTVPQRCI
GFARRRVGRGGRVLLDRAHSDYDNTFHQLDLEMLSSSQHSSISQFANTSETNTSDKSFSKDLSQILVNIK
SCRWRHFRPRTPSLHDSDNDELSLFFFQALQGYEMLCLRRLQLRVFVCLCELSLLRESETSLGLVHQTLN

HSNGSKSLQYSEFTVLPEYHIRLCLEEMLTIMQSKDVIEWLNWRKKMICNYILDDMCLSHFNDDRKNMLL
TDDDQLMITNENDIQSPYCCSPLSFDRSERFSVVPWLSQKEDEKASQPAGGLLGSSGLCLQVHTGSTALV
PSAVLGISPDPATAVCYGKLVRKGLCVGYHCFSCIWKQEMVFSFDRNSFSEAFTAEQYQQHQQQLALMQK
QQLAQIHQQQANSNSSANTSQGFVSKTLDSVSAQFAASALVTSEQLMGFKMKDDVVLGIGVNGILQASGV
YKGLHLSSTTPTALVHTSSSSAAGSALLQPSNITQTSSSHSALSHQASAANSATTQVLIGNNIRLTVPSS
VATVNSITTLNARHIPRTLSAVPSSALKLAAATNCQVPKVPASSSVDAVPRENHETEKPALNNIADNTVA
MEVT

Epc-N
EpcC 3.7e-142

>XlaevisAAH80994
MSKLSFRARALDATKPLPIFRGNDMPDLNDCVSINRAVPQMPTGMEKEEESEHHLQRAISAQQVFREKKE
CMVIPVPEAESNVHYYSRLYKGEFKQPKQFIHITPFNLDYEQPDYDMDSEDETLLNRLNRKMEIKPLQFE
IMIDRLEKASSNQLVSLQEAKLLLNEDDYLIKSVYDYWVRKRKNCRGPCLIPQVKQEKRDGSTNNDPYVA
FRRRTEKMQTRKNRKNDEASYEKMLKLRREFSRAITILEMIKRREKTKRELLHLTLEVVEKRYNLGDFGG
EILNEIKIPKADKEIYAIPPSLHNGNHHKVPECKVKNTHHLNVKDEVLEIVRLKKKYPKKPKADILVTPL
PQANSEPLAVINRSDIKQYDFQSSDDDEFPQVPSPLSELEEENDPDGSYAFRRRAGCQYYAPHLDQMNDT
PETTDLSGLARHRNRHCLTTLTVPRRCIGFARKRLGRGGRVIMDRLSTEHDSVLKQIDPEMLSGFSSSSH
IAQPPSSPSRTNASDRHCENRLSLPEILSNIKSCRLQCFQPRLLHTQDSDREECTSRLGQAVNLKRVSSS
LLNTSKNGITVTGGITEEQFQTHQQQLVQMQKQQLAQLQQKQQSQQSLQQSHPKAQGSGSSDCMSKTLDS
ASALFAASAVVNSPAPVRSEVNKDQNAGHSNLNGVVQPSGTAKTLYSTNMALSSSPGISTVQLVRTVGHP
TTNHLIPTLCTSNPQTLTMGNSCLANTVHLNNVSVVSPVNVHLNTRTSAPSPTALKLATVAASMDRVPKV
TPSSAMSSIARENHEPERLGLNGITDTTVAMEVT

Epc-N
EpcC 1.6e-138

>DrerioAAH48890
MSKLSFRARALDAAKPLPIYRNKDLPDLTDCVSINRAVPQMPTGMEKEEESEHHLQRAISAQQVFREKKE
SMVIPVPEAESNITYYDRLYKGEFRIPKQLIHIQPLGLDNELPDYDMDSEDETLLNRLNRKMELKPVQFE
TMMDRLEKASTNQLVTLQEAKLLLNEDDYLLKSVYDYWVRKRKNCRGPSLIPQIKQEKRDGSTNSDAYVA
FRRRTEKMQTRKNRKNDEASYEKMLKLRREFSRTISILEMIKKREKSKRELLHLTLEVFEKRYQIGDFTG
EILNEVTVPLAEKTVYPAPISLPISSRHKTENKIKSHKIGPKHLHPFPVKPEPHFDFVRSHKKYNKRPKL
DTFRQTGWPERIQTINKADIKQYDFHSSGEEDYPLSPASEPDEENDPDGVFAFRRKAGCSYHAPVVDQSC
SPHWQQTGQDQLWQRNCLTALSVPRRCIAVACRRVGRGGRVVLDRTSSDLDHSLGHLDPDMLYPSAGSCV
PDLPVHTNTNSHVSQRSLTQLLGDIQACRWKFFRPRPLQDNSRAEDKKGTPPVEKGGGTTLSNSVSGGFT
EEQFQSHQQQLVHMHKQLQQQELQQNSTAAEAHLHLHTLDSAGAHFAASAVINTPNNENRPQIASVNGVL
PNSGSFRQVKVNRSAPSGGGGAGGESGSLVRSPQLAVPQSLPHGHGHLSTVSAVSPAHTHHTARLCAPSP
SALKLASVASSLDRVPKVTPTSAIDIARENHEPERLALNGLSETTVAMEVT

Epc-N
EpcC 1.3e-25

>CfamiliarisXP_535156
MGTEQGARQRQPRASLDVKIIAVKVRPKWHPAPTVSGEVSAAPEMSKLSFRARALDASKPLPVFRCEDLP
DLHEYASINRAVPQMPTGMEKEEESGKKRPDLRRYRPRFREGVLPGRYRTKEHHLQRAISAQQVYGEKRD
NMVIPVPEAESNIAYYESIYPGEFKMPKQLIHIQPFSLDAEQPDYDLDSEDEVFVNKLKKKMDICPLQFE
EMIDRLEKGSGQQPVSLQEAKLLLKEDDELIREVYEYWIKKRKNCRGPSLIPSVKQEKRDGSSTNDPYVA
FRRRTEKMQTRKNRKNDEASYEKMLKLRRDLSRAVTILEMIKRREKSKRELLHLTLEIMEKRYNLGDYNG
EIVSEVMAQRQPMKPTYAIPIIPITNSSQFKHQEAMDVKEFKANKQDKADLIRPKRKYEKKPKVLPSSAA
ATPQQTSPAALPVFNAKDLNQYDFPSSDEEPLSQVLSGSSSEAEEENDPDGPFAFRRKAGCQYYAPHLDQT
GNWPWTSPKDGGLGDVRYRYCLTTLTVPQRCIGFTRRRVGRGGRVLLDRAHSDYDSMFRHLDLEMLSSPQ
HSPVNQFANTSETNTSDKSFSKDLSQILVNIKSCRWRHFRPRTPTLHDSDNDELSCRKLYRSINRTGTAQ
PGTQTCSTSTQSKSSSGSAHFAFTAEQYQQHQQQLALMQKQQLAQIQQQQANSNSSTTTAQNLASNQQKS
GFRLNLHHSHSIQGLERALQGFVSKTLDSASAQFAASALVTSEQLMGFKMKDDVVLGIGVNGVLPASGVY
KGLHLSSTTPTALVHTSPSTAGSTLLQPSNMTQTSGSHSALSHQVTAANSATAQVLIGNNIRLTVPSSVA
TVNSIAPINARHIPRTLSAVPSSALKLAAAANCQVSKVASSSAVDSVPRENHESEKPALNNIADNTVAME

VT

Epc-N
EpcC 3.2e-177

>HsapiensBAC03857
MVIPVPEAESNIAYYESIYPGEFKMPKQLIHIQPFSLDAEQPDYDLDSEDEVFVNKLKKKMDICPLQFEE
MIDRLEKGSGQQPVSLQEAKLLLKEDDELIREVYEYWIKKRKNCRGPSLIPSVKQEKRDGSSTNDPYVAF
RRRTEKMQTRKNRKNDEASYEKMLKLRRDLSRAVTILEMIKRREKSKRELLHLTLEIMEKRYNLGDYNGE
IMSEVMAQRQPMKPTYAIPIIPITNSSQFKHQEAMDVKEFKVNKQDKADLIRPKRKYEKKPKVLPSSAAA
TPQQTSPAALPVFNAKDLNQYDFPSSDEEPLSQVLSGSSEAEEDNDPDGPFAFRRKAGCQYYAPHLDQTG
NWPWTSPKDGGLGDVRYRYCLTTLTVPQRCIGFARRRVGRGGRVLLDRAHSDYDSVFHHLDLEMLSSPQH
SPVNQFANTSETNTSDKSFSKDLSQILVNIKSCRWRHFRPRTPSLHDSDNDELSCRKLYRSINRTGTAQP
GTQTCSTSTQSKSSSGSAHFAFTAEQYQQHQQQLALMQKQQLAQIQQQQANSNSSTNTSQGFVSKTLDSA
SAQFAASALVTSEQLMGFKMKDDVVLGIGVNGVLPASGVYKGLHLSSTTPTALVHTSPSTAGSALLQPSN
ITQTSSSHSALSHQVTAANSATTQVLIGNNIRLTVPSSVATVNSIAPINARHIPRTLSAVPSSALKLAAA
ANCQVSKVPSSSSVDSVPRENHESEKPALNSIADNTVAMEVT

Epc-N
EpcC 1.1e-159

>HsapiensAAH36529
MSKLSFRARALDASKPLPVFRCEDLPDLHEYASINRAVPQMPTGMEKEEESEHHLQRAISAQQVYGEKRD
NMVIPVPEAESNIAYYESIYPGEFKMPKQLIHIQPFSLDAEQPDYDLDSEDEVFVNKLKKKMDICPLQFE
EMIDRLEKGSGQQPVSLQEAKLLLKEDDELIREVYEYWIKKRKNCRGPSLIPSVKQEKRDGSSTNDPYVA
FRRRTEKMQTRKNRKNDEASYEKMLKLRRDLSRAVTILEMIKRREKSKRELLHLTLEIMEKRYNLGDYNG
EIMSEVMAQRQPMKPTYAIPIIPITNSSQFKHQEAMDVKEFKVNKQDKADLIRPKRKYEKKPKVLPSSAA
ATPQQTSPAALPVFNAKDLNQYDFPSSDEEPLSQVLSGSSEAEEDNDPDGPFAFRRKAGCQYYAPHLDQT
GNWPWTSPKDGGLGDVRYRYCLTTLTVPQRCIGFARRRVGRGGRVLLDRAHSDYDSVFHHLDLEMLSSPQ
HSPVNQFANTSETNTSDKSFSKDLSQILVNIKSCRWRHFRPRTPSLHDSDNDELSCRKLYRSINRTGTAQ
PGTQTCSTSTQSKSSSGSAHFAFTAEQYQQHQQQLALMQKQQLAQIQQQQANSNSSTNTSQGFVSKTLDS
ASAQFAASALVTSEQLMGFKMKDDVVLGIGVNGVLPASGVYKGLHLSSTTPTALVHTSPSTAGSALLQPS
NITQTSSSHSALSHQVTAANSATTQVLIGNNIRLTVPSSVATVNSIAPINARHIPRTLSVVPSSALKLAA
AANCQVSKVPSSSSVDSVPRENHESEKPALNNIADNTVAMEVT

Epc-N
EpcC 1.9e-159

>TnigroviridisCAG05437
MAVRIRKIPFDLVPVQEHHLQRAISAQQVYGEKRENMVIPVPEAESNITYYESLYPGEFKMPKQLIHIQP
FSLDTELPDYDLDSEDETFVNKLKKKMEISALQFEEMIDRLEKGSGQQLVSLQEAKLLLKEDDELIKEVF
DYWSRKRKICKSSLLIPVVKQEKRDGSSTSDPYVAFRRRTEKMQTRKNRKNDEVSYEKMLKLRRDLSRAV
TILEMIKRREKSKRELLHLTLEIVEKRNTMPDYGCEVIAEALAQRALVKPVYTFPIIPLSNSNQYRHQDH
LDMKDYKKPEKTEAIRTKRKYEKKPKIPPLSAPQHSGPSIFSLKDLNQYDFPSSDEEPFSQIHSGSSEAE
EENDPDGCYAFRRKAGCQYYASRQDQDGNWPWVAPSDGGLGDVRFRYCLTSLNVPRRCIGLARRRVGRGG
RILLDRAHADVDGVCQGLNSDIESAPLASPLPSSLLYGPNTSTSETNTSDPTTCSHHSSLLFTRSPFSKT
PLDLSQILLNIKSYRWRHFRPRTITHHPLGEGGLSLRGFRDFSRTVSGLTRTLSGGTNTVNRTGPAPTPV
HVLTAEQYQQHQEQLVLMQKQLEQSQLQANNPTTTITTPNNTQVSGDDDDDDDDDQVVRPAIVSKMLDQA
SAQFAASALVTTDQLLTFKSKEDLVVTGGLNGVVTGAGAYKSLHLLSTPTTHHQVNQSTNTTTSAVPTFL
QPSSNSTSPSPAMVVPSSLSSTPSAHAAAALGILGCGTTNNSTPSSTQVLIGNNICLSVPTATSLAGRHI
PRTLGSVPSSALKLSATANLQMPKVSGASSVDLGSRDNHDEDKPALNSIADNTVAMEVT

Epc-N
EpcC 2.8e-45

>RnorvegicusXP_225457
MSKLSFRARALDASKPLPVFRCEDLPDLHEYASINRAVPQMPTGMEKEEESEHHLQRAISAQQVYGEKRD
NMVIPVPEAESNIAYYESIYPGEFKMPKQLIHIQPFSLDAEQPDYDLDSEDEVFVNKLKKKMDICPVQFE

EMIDRLEKGSGQQPVSLQEAKLLLKEDDELIREVYEYWIKKRKTCRGPSLIPSVKQEKRDGSSTNDPYVA
FRRRTEKMQTRKNRKNDEASYEKMLKLRRDLSRAVTILEMIKRREKSKRELLHLTLEIMEKRYNLGDYSG
EIMSEVMAQRQPVKPTYAIPIIPITNSSQFKHQDVTDAKEFKVNKQDKADLIRPKRKYEKKPKVLPSSAA
APQQPSPAALPGFSAKDLNQYDFPSSDEEPLSQVLSGSSEAEEENDPDGPFAFRRKAGCQYYAPHLDQTG
NWPWTSPKDGGLGDVRYRYCLTTLTVPQRCLGFARRRVGRGGRVLLDRAHSDYDSMFHHLDLDMLSSPQP
SPVNQFANTSETNTSDRPSSKDLSQILVDIKSCRWRHFRPRTPSLPDSDSGELSSRKLHRSISRAGPAQP
GAHTCSASTQSRSSSGSTHCAFTAEQYQQHQQQLALMQQQQLAQTQQQQANSSSSTAAPQNLASNQQTRG
SGLNVQGLERTLQGFVSKTLDSASAQFAASALMTSEQLMGFKMKDDVVLGIGVNGVLPASGVYKGLHLSS
TTPTALVHTSPSTAGSTLLQPSNITQTSGHSALSHQVTAANSATTQVLFGNNIRLTVPSSVPTVNSIAP
INARHIPRTLSAVPPSALKLAAAANCQVSKVPSSSSVDSVPRENHESEKPALNNIADNTVAMEVT

Epc-N
EpcC 8.7e-171

>HsapiensAAK60501
MEHHLQRAISAQQVYGEKRDNMVIPVPEAESNIAYYESIYPGEFKMPKQLIHIQPFSLDAEQPDYDLDSE
DEVFVNKLKKKMDICPLQFEEMIDRLEKGSGQQPVGLQEAKLLLKEDDELIREVYEYWIKKRKNCRGPSL
IPSVKQEKRDGSSTNDPYVAFRRRTEKMQTRKNRKNDEASYEKMLKLRRDLSRAVTILEMIKRRERSKRE
LLHLTLEIMEKRYNLGDYNGEIMSEVMAQRQPMKPTYAIPIIPITNSSQFKHQEAMDVKEFKVNKQDKAD
LIRPKRKYEKKPKVLPSSAAATPQQTSPAALPVFNAKDLNQYDFPSSDEEPLSQVLSGSSEAEEDNDPDG
PFAFRRKAGCQYYAPHLDQTGNWPWTSPKDGGLGDVRYRYCLTTLTVPQRCIGFARRRVGRGGRVLLDRA
HSDYDSVFHHLDLEMLSSPQHSPVNQFANTSETNTSDKSFSKDLSQILVNIKSCRWRHFRPRTPSLHDSD
NDELSCRKLYRSINRTGTAQPGTQTCSTSTQSKSSSGSAHFAFTAEQYQQHQQQLALMQKQQLAQIQQQQ
ANSNSSTNTSQGFVSKTLDSASAQFAASALVTSEQLMGFKMKDDVVLGIGVNGVLPASGVYKGLHLSSTT
PTALVHTSPSTAGSALLQPSNITQTSSSHSALSHQVTAANSATTQVLIGNNIRLTVPSSVATVNSIAPIN
ARHIPRTLSAVPSSALKLAAAANCQVSKVPSSSSVDSVPRENHESEKPALNNIADNTVAMEVT

Epc-N
EpcC 8e-161

>MmusculusAAC64272
MEHHLQRAISAQQVYGEKRDNMVIPVPEAESNIAYYESIYPGEFRMPKQLIHIQPFSLDAEQPDYDLDSE
DEVFVNKLKKKMDICPLQFEEMIDRLEKGSGQQPVSLQEAKLLLKEDDELIREVYEYWIKKRKTCRGSSL
IPLVKQEKRDGSSTNDPYVAFRRRTEKMQTRKNRKNDEASYEKMLKLRRDLSRAVTILEMIKRREKSKRE
LLHLTLEIMEKRYNLGDYSGEIMSEVMAQRQPVKPTYAIPIIPITNSSQFKHQDATDSKEFKVNKQDKAD
LIRPKRKYEKKPKVLPPSAAAPQQQSPAALPGFSAKDLNQYDFPSSDEEPLSQVLSGSSEAEEENDPDGP
FAFRRKAGCQYYAPHLDQTGNWPWTSPKDGGLGDVRYRYCLTTLTVPQRCLGFARRRVGRGGRVVLDRAH
SDYDSMFHHLDLDMLSSPQPSPVNQFANTSEPNTSDRSSSKDLSQILVDIKSCRWRHFRPRTPSLPDSDS
GELSSRKLHRSISRAGAAQPGAHTCSTSTQNRSSSGSAHCAFTAEQYQQHQQQLALMQQQQLAQTQQQQ
ANSSSSAAAQQGFVSKTLDSASAQFAASALMTSEQLLGFKVKDDVVLGLGVNGVLPASGVYKGLHLSSTT
PTALVHTSPSTAGSTLLQPSNITQTSGHSSLSHQVTAASSATTQVLFGNNIRLTVPSSVPTVNSVTPIN
ARHIPRTLSAVPPSALKLAAAANCQVSKVPSSSSVDSVPRENHESEKPALNNIADNTVAMEVT

Epc-N
EpcC 2.4e-144

>MmusculusNP_081773
MSKLSFRARALDASKPLPVFRCEDLPDLHEYASINRAVPQMPTGMEKEEESEHHLQRAISAQQVYGEKRD
NMVIPVPEAESNIAYYESIYPGEFRMPKQLIHIQPFSLDAEQPDYDLDSEDEVFVNKLKKKMDICPLQFE
EMIDRLEKGSGQQPVSLQEAKLLLKEDDELIREVYEYWIKKRKTCRGSSLIPLVKQEKRDGSSTNDPYVA
FRRRTEKMQTRKNRKNDEASYEKMLKLRRDLSRAVTILEMIKRREKSKRELLHLTLEIMEKRYNLGDYSG
EIMSEVMAQRQPVKPTYAIPIIPITNSSQFKHQDATDSKEFKVNKQDKADLIRPKRKYEKKPKVLPPSAA
APQQQSPAALPGFSAKDLNQYDFPSSDEEPLSQVLSGSSEAEEENDPDGPFAFRRKAGCQYYAPHLDQTG
NWPWTSPKDGGLGDVRYRYCLTTLTVPQRCLGFARRRVGRGGRVVLDRAHSDYDSMFHHLDLDMLSSPQP
SPVNQFANTSEPNTSDRSSSKDLSQILVDIKSCRWRHFRPRTPSLPDSDSGELSSRKLHRSISRAGAAQP
GAHTCSTSTQNRSSSGSAHCAFTAEQYQQHQQQLALMQQQQLAQTQQQQANSSSSAAAQQGFVSKTLDS
ASAQFAASALMTSEQLLGFKVKDDVVLGLGVNGVLPASGVYKGLHLSSTTPTALVHTSPSTAGSTLLQPS
NITQTSGHSSLSHQVTAASSATTQVLFGNNIRLTVPSSVPTVNSVTPINARHIPRTLSAVPPSALKLAA

AANCQVSKVPSSSSVDSVPRENHESEKPALNNIADNTVAMEVT

Epc-N
EpcC 2.4e-144

>ZmaysAAM13423
MSRLSFRPRPLDIHKKLPILKSVREFEDEEPGIAPLSSARAGVLLRHSGADFTASGANNATEGEGNQALS
KKNAQEIPTPQFDGVDTYERDYTRTFAQPTSYIRGRGARAEIGEFVEYDLDNEDEDWLEEFNSERKNINP
EKLEALLFKLEVLDHRARERAGAITPTFIGPVPVLLQLDAAMEALQYLSVRNAVFQAVYNYWKSKRERWQ
KPILRRLQPPPPVNDTNPYNVFRPREKAHRLHTRRMQRRENNIQSFEKLRLVRRNLEQAKALMDALIKRE
ETKREAMECQVNLQRVQMKYKHEAQLVEDGTTLSGFQQTSSRFESSDDDVDSDDTATEQTYIRPAVFHPR
FSANKLSVIPPLRIKRERELKRRPQQNGCVFKRDPEEPVFLFTRPLDPEKLVAAGIKPPPDPPIENGATS
PPFRCRGRIGRGGRIIFDRWNPLLRTPIDQETYYVPYGHRPPSPEG

Epc-N

>OsativaBAD03206
MSRLSFRPRPLDIHKKLPIVKSARELEDDETTLALRAAPPVLRHSQPEPAADGEAHPTSSKKNVQEIPTP
QYDDVDTYERDYTRTFAQPTSYIRARGARAEIGEFVEYDLDNEDEDWLEDYNNERKNLNPEKLEVLLFKL
ETLDHKARERAGIITPTFLGPIPVILQLDSAMEALQYLSVRYAVFQAVYNYWKSKRERWQKPILRRLQPP
PPVNDTNPYNVFRPREKAYRLHTRRMQRRENSVQSFDKLRVVRRNLEQAKALMGALIKREERKRETMECE
VHLRRIQMRYKHEAQLIDDGIALSGLQQAGSSEDDYADSDDTANEQPYVRSVAFHPRFPDNKLSAVPPLR
LKRERELKRRPHQNGWLFKRVPEMRDPEEPVMLFTRPIDPDKLKMAGIRPPLDPPIDSGTTAPPFRWQAR
IGRGGRIIFDRWNPFLQVPVGQETNHRPSMPEG

Epc-N

>DpseudoobscuraEAL25124
MSKLSFRARHLDPSKQMPIYLAEELPDLPEYSAINRAVPQMPSGMEKEEESEHHLQRAICTGLIIPTPEV
LQTDQPFYDTYYPPDYKMPRQMIHMQPLGLDTEVPDYDMDKADMEWLSQQERLELTELKFEQLMDLLEKS
SGQTVVTLNEAKSLLNQDDETSISLYDYWLNKRLKMQHPLILTVKTESRPGASSNNPYLAFRRRTEKMQT
RKNRKNDEASYEKMLKLRRDLQRATTILEMVRRREETKREQLKTTVNIFEKRVELRDFNGAVYSELNAQY
KSTRPAYNSLYTNQYSQGAAAAVGTAGAILAALPPGLLASGGAAGNTNVYASPSQYLNTSTMDTIHSGSG
NGNGNGSSSRKEKRPYKKRKHKLPRDRSGASPAHHLPHHLHNLSRQQSASPAANDSILDSEDDDYLNGGL
NGNQLGSESEDEAPFAFRRKPSCIYLPTRHRNGRWPYDSADEETETSRSTGPYSDPKYRYTLTSINYPSP
RCIGFARRRVGRGGRILLDRATTNFDDVWSQLDYTVMESVVKSNNAQRRNNQLSKPELLPLAKPCSPPTV
VDLVVPTSKHIDSVDATSLPPDAIVKSEPTSHSVDKPIAGGSAGDSLLPLPLDKEVDAEVLDLDGEEDDL
VTDDENVSRLSFYSSAVTLSQSSHDMRLKRRRLRHKKQQMRELNAAKRLKRSSDAAATGGTGGEPAAGGE
EGPFTAPLCPLPRAYLQRLVLALGQKLKQEEAMEVCENLAPQEEKSSAAEELQQPAPPRANNQHHHRSSS
SNMNKDVSISEKINVIKLEAEGNEDVGREQPVASTLSMLGPSMEDVAKTIKRELIDADDSNEPLSIIRTA
TVAATVLQASPDPELMDDEAEDDVNLAQLSSIIRHTAVKKELEAGYGYPQELKEETVCLNQLPDVIRLQN
LRNAQTCSKPRDLYIDATAEDMDVDYLGGLSPTSSQRLDICNELLSEIRRDWLHFRPKTPTDELSDSNDG
EGKPCGDSGVHAVDWTQDTPISVELNRLGKQEEIESDTSSYFLSNAFKYTDLDSDAQLIQTAYAQDYARG
TSKSPNCSGSGSGSGSALEFNLSAGDSLNDINNLLGDDEDDNHEHMLDNILQECGMDDPKALNQATSFWN
GILDGEAAVDEVEIADQLLDCIDEKKPKVGGEASKRAGRLRKPRPVNVPVGSSLFTVCPTQESLKNREVF
FSQEVVTPKEEPQPATETETAPIINIPAQIIPQKPQMAQIQTQTQQLQLQSQTQAQVQAQTAPQQVTQLL
NQFANASPQIIARTEYGGGAAVGDIVTITPHTGSSPLPTLSAQQQLQHQLAQAQLRNVTVQQRQGTPQQV
QVQQQQQQPTQQLLFQMQRDGIGSPVITTQSIVTSSRSLTPLNAAGAGAAGNHMIYTTGSGEIITAAAAA
AAQKVTYAIQKSSGSGGGATTTSIGPNTVITLSNVKLQSDDNCSAGGAGSGSSVNIINTASGQQLAVHSI
AGSTTTTPLIVTTPTRNNVVQQHSQQQLVQQQPIVWRQVSGTNTAVATTAVLSTTADTSPAAVGNKILWT
SRGTQKRQLNGPENTDINKLLLNRKFSQRKVIGQHPGGGQQIQQQAQQQIQLTKQQLQQLVQHEDDLVTA
TTTINSGDQMDTGESLQQQHHQHQQLQKLSKVIKMSPFPLLVSDLNLPNSAAAIAANHNYSLQPATAIIS
SQAAPQQANKIFLTSSNSGGGGNFTNFTASELQAAAAAVAASNAGGQKLHYKELTNSNLKLNFVSSTGEA
LASQAPQQVGATTVVLNKAQQQQVHHQKLKTVAAANAQQVQQGDKRVLYTSLLNVKPLQKNNSGQMQMQL
GPGGLQQVLRGRVNSSIITRTLPLGTTVNSSAGGGAGGAPATIRQLVTTNTNNVGSSDGALLSAKDVGKS
LTVEQVMVSANNGGVVMPTSNNNNNNGSG

Epc-N

>OsativaBAD29358
MSRLSFRPRPLDIHKKLPILKSAREFEDDDPTAAAVAVARAGVLLRQSAPELTAATTATEGEGNPTPTKK
NIQEIPTPQFDAVDTYERDYTRTFAQPTCYIRGRGARAEIGEFVEYDLDNEDEDWLEDFNNERKNLNPEK
LEVLLFKLEILDHKARERAGAITPTFIGPVPVLLQLDAAMEALQYLSVRYGVFQAVYSYWKDKRERWQKP
ILRRLQPPPPVNDTNPYNVFRPREKAHRLHTRRMQRRENNIQSFEKLRMVRRNLDQAKALMDALVKREET
KREAMECEVNLRRIQMKYKHEAQLVDEGTALSGFQQVSSRFGSSEDDYADSDDTTTEQPYIRPPVFRPRF
ADHKLSVIPTLRIKRERELKRRPQQNGWVFKRDPEEPVLLFTRPLDPEKLLAAGIKPPPDPPIENGATMP
PFRCRGRIGRGGRIIFDRWNPLLQTPIGQETSYYVPYSRRPPSPES

Epc-N

>AthalianaBAC42110
MSRLSFRPRPLDIHKKLPILKSFKDFEDDETPTSTTRNSQLLRIASVEVDNEVAPVPSKKPASEIPTPQF
VIVDTYERDYSPTFGQPASYLRARGARSELGEFVEYDLDNEDEDWLYEFDKDKKELPPEKLEIIIFKLEV
LDHKTRERAGVITPTLGSPVPVLLQFDAASDVLQVLSINYGTFQAIFNYWKEKRKRWQKPILRRLQPPPP
VNDTNPYNVFRPREKVHRLHTRRMQRRENNVQSFEKLRQVRRNLGQAQSILEALIKREEKKRDVMDSEVS
LQRIQLQYRHETELLEDSLAMPGFQPTTTSYKFGSSDDELMDSDDHTSTRVRTRPAVIPSSRFTNLNLNA
SHPGGIKQEVRRRQSQHGWLHRLDPNEPVMLFTKPLVPDKLAAAGIVPPTPDSSPGQPPSRFQGRIGRGG
RIIFDRWNPLMQSHINCGNSYYIAPNHRSTNFD

Epc-N

>NcrassaEAA31357
MATRKVRYKKLSVKTQLAVLREDQIEASEYESLTSENQIATGVEQAEENEYHLQAVLKGAGVAADQEIPV
PPPQQSELDYDQFYPQKVAKTSTYIRFSQTVEECISCLYDMTEDDETFLKSYNMKLTPSARLSEDDFERI
MDVYEDMAANITPFSAIDQTVPSYQEMLRGLEPLDSTKVMVHAKQIYEYWKSRREISKNRPLNPTLKFET
HAESDELDPYVCFRRREIRQTRKTRARDVQSADKLKRLRKELEEGRQLILAAHNRELLKADMLKVERAIF
DQRAIIKEQKLRLGIRTGDEDLVNQKPQKRKAPEAPSAQRPPPPPQIRMPVRPDGRPAESDLVQLSDRLA
EKNAELIIEIEKKIQNHIDWNKNYVDLTGKPLSPVQGPRQDLGFRPAKTQYLMTPPASASSGSMDEPTPM
DLDKPKPNPPPPVKFRGVAQDEQSLAHPPSYRRRIGRLNRLWIDRRGLPSPARDLSEEQSDRWKYDQSSD
DEDDAPVYMLDPFDTKALRYRASIPLQTVTRPPPPVINRQFIPPGAVPQQLAQSSFAPGQPQPQSQPQPN
QSQSLPLPQPQQPVAQPQPQPQPQAQPVS

Epc-N

>PyoeliiyoeliiEAA22977
MQSLLENSELKDEKKKKKRRDIAIPRFKICENDDYKLTKFEKPTHYIRYELYRDQVTGIRLSDGCIIHYD
LLKEDEIFLESLNSYMNIHVSNDDFCKLMDKFEKLTGYSENKEEINLKDALNAASELKINYKSNIIKDIH
TYWKAKRKKLGRPLLRMFWNNAQNILSHYSVFRSRVKEKMTLRKHKKKNGEVIVKMQELIEDFKRLDRIL
RKMKQRDEKKLLLLQLNSILFDQRKNEIKDKTYVCPMWDYFKDYKIDKIYKKFKKDKHYKNLHANNNHVH
HHHNHNHNSIHSRKINKKNDTIKDSSFSEHMYSSRKINPNEYKNVVLIKRRGRNNRLWIDRKYINNNEMD
NVNDLNYYCDLSYVINDHNDDPPSNLKKNYNDNMDNDPASTKKHIPIKLDIKSTDLKYLDSNFFSNTNKN
TLTTSYGDKIINGGVDGENQGINMIEKINETKKEKKRRKRKKENNNEDMSFDNKTTNFPPLETYKNSLGL
VKRENMPIFVDEFSKDMGQTRRDNRDGYICNIQNNGNDYGVSDMIENNEITLDNNYVYEKNKLDEMINEN
KRAMEAIRGGSQNIGGLYDEYYDNNVEMGNKIKKVPTSVNNINSVNEGSNINEVEKNNNLFYPNMRHNNK
TIFQFDDIINPHINRNDYLFCLIKKEMILLKTLRKILHKILK

Epc-N

>PbergheiCAH98686
MTKSAGNHGKAAKGKGLDINKKFLIIKSSEDIKKLIKNDNPTNEEISEMQSLLENSELKDEKKKKKKRDI
AIPRFKICENDEYKLTKFEKPTHYIRYELYRDQVTGIKLNDGCIIHYDLLKEDEIFLESLNSYMNIHVSN
DDFCKLMDKFEKLTGYSDNKEEINLKDALNAASELKINYKSNIIKDIXAYWKAKRKKLGRPLLRMFWNNA
QNILSHYSVFRSRVKEKMTLRKHKKKNGEVIVKMQELIEDFKRLDRILRKMKQRDEKKLLLLQLNSILFD
QRKNEIKDKTYVCPMWDYFKDYKIDKIYKKFKKDKHYKNLHTNNHMHHHHNHHYHSIHSRKINKKNDTLN
NFSFSEHMYNPRTMNPNEYQNVVLIKRRGRNNRLWIDRKYINNNEMNNVNNLNYYCDLSYVINDHIDSSS
NLKKNYNGNMGNDPSSTKKHIPIKLDIKSTDLKYLDSNFFSNSNKNTITTTYADKISNGGVDGENQGINM

IEKINETKKEKKRRKRKKENNNEDTFSGNKTTNFPPLETYKDSSGLVKRENLPILIDEFSKDVGQSHHDN
RDEYICNIQNNGNDYAVSDMIKNNEITLDNNYVYEKNKLDEMINENKRAMEAIRGGPQSIGGLYYEYYDN
NVEMGSKIKKVPTSVNNINSVNEGSNINEVEKNNHLFYPNMRHNNKTIFQFDDIINPHLNRNDYLFCLSK
YASVQKRILFYLENILNFDTFNFKSKKGDDSAQNPSKNTSQNSQINIPPANNDIVKNAGETKCD

Epc-N

>ChominisEAL37343
MSQTSTRNARPKTLDPQKKLQVVFSISQLEKALEEGNDKLEPDEVRNIIDEEKQRIQEVINQMRAKEKEK
SSQKQSTGNDSSVSSSTSKDKDNSNTNSHAGSATSSGGSSCAGGLGGSSGSLSGAAASSILVPPVEICPL
NVPKLIAFQRPEHYIRFPVHKEFVSGIRLEDGTVVHYNMVKIDEEFLQSLSEHMKVGVSESDFIKMIDFM
EKSTGRGSEISFDEALQICRERGLSSIKNQQALLIYKYWRMRRHKLGKPLLRHFWPITSPHDSSPYACFR
PRVREKMTLRRPRRNNKETLEKLEKLLDDFRKVEKSFRKLRQRDEKKLLLAELDTCIFDQRRHEITDPFY
RCPIWDRLKNYKQQKRQHKKEMRGVSQLSIGTGAGGSIAQLFTGGGVMGLTAGSPLLIQGGDFSSSFNFG
DLSMFIKKLSSEIGNLDMLLKYSWENETSLLSYYYYGSLQLTSSIFNVFRTYAGKQDHTEAIKNGENTDQ
SFSVMSDGNKGEVIMAQTPGRSSNIESAKLNEDCQRSQENQAQPGPSPAFCRIAIPNYIQSIYKNGVDLA
RLEPLQSLRTPCRPHRLVRRRGRGGRIWFDRHLLFQDNFEPHFTDPDKLDLASNCGGSNQFIISSYFRKT
ASMCDLIDELCTEGNFDPFGNSSNGNNTQGISVSISSSSNNTGIHHSSMNDLPKSTIRLSGTMNNPNSNL
GAASMPINPVIGVNNTNSAAGFSTGNQNPTNRSIPRNASYLDAEMTC

Epc-N

>MgriseaEAA55275
MATRKVRYKKLNVKTPLPVLLFSEIDSNEYEALKNEQIQTGVESAEENEYHLQAVLSTTGVAADKEIPVT
QTQESNINYDELYPTKFVEPTNYIRFSQTVEECIGCPYDMTEDDEAFLAKYNKDLAAGTAALSEDDFEKI
MFVFEDTASVQAPYAAVDNTVVSYDHMVPPLTELELPRLMLHAKAIYEHWKSQRQAKKGPLHPSLKFETH
QESDDLDPFVCFRRREVRQTRKTRARDTQIADKLKRLRRELEEGRELIALAHGREVMKREMLNVDRAIFE
QRCNVKKVKIKLGIKTDDEDLYNTKPQRKKPAEAQPVQQRAAAGPARVAPRSDTRASDVDLGLLSELKAQ
REEEMRVDIETKIENHKKWNEKYVDLTNSSLVDKKPETEQEPDPAKWRPVKVQYLMTPPNSASAEDLEEP
TPMELDAPAPAPVRRFHGLSADDEDYSPDPMEYRRRVGRLNRLWVDRRPVKKQLVDVVPTRFPVVSDRWK
YDCDSSDDEDSVYEVDPFDLRALKFRATIPLGSAAPRRIEGSAAAPVTNGTARPALPWSGGGQPPSQQPQ
QQQQQPQPQPQLQMQQSA

Epc-N

>CparvumEAK87670
MSQTSTRNARPKTLDPQKKLQVVFSISQLEKALEESNDKLEPDEVRSIIDEEKQRIQEVINQMRAEEKEK
SSQRQSTGNDSSVSSSTSKDKDNSNTNTHAGSATSSGGSSCAGGLGGSSGSLSGATASSILVPPVEICPL
NVPKLIAFQRPEHYIRFPVHKEFVSGIRLEDGTVVHYNMVKVDEEFLQSLSEHMKVGVSESDFIKMIDFM
EKSTGRGSEISFDEALQICRERGLSSIKNQQALLIYKYWRMRRHKLGKPLLRHFWPITSPHDSSPYACFR
PRVREKMTLRRPRRNNKETLEKLEKLLDDFRKVEKSFRKLRQRDEKKLLLAELDTCIFDQRRHEITDPFY
RCPIWDRLKNYKQQKRQHKKEMRGVSQLSIGTGAGGSIAQLFTGGGVMGLTAGSPLFIQGGDFSSSFNPG
DLSMFIKKLSSEIGNLDMLLKYSWENETSLLSYYYYGSLQLTSSIFNVFRTYAGKQDHTEAIKNGENTDQ
SFSVMSDGNKGEVIMPQTPGHSNIEPAKLNEDCQQSQENQAQPGPSPAFCRIAIPNYIQSIYKNGVDLAR
LEPLQSLRTPCRPHRLVRRRGRGGRIWFDRHLLFQDNFEPYFTDPDKLDLASNCGGSNQFIISSYFRKTA
SMCDLIDELCTEGNFDPFGNSSNGNNTQGISVSISSSSNNTGIHHSSMNDLPKSTIRLSGAMNNPNSNLG
AASMPINPVIGVNNTNSAAGFSTGNQNPTNRSIPRNASYLDAEMTC

Epc-N

>AnidulansEAA57907
MTRYAGLGRTRPKKLTPKAPIPIYREHQIDDLEEEIQNGLQQVETGVEKAEESEYHLQVAINAVASGRVV
NEAHIPTPETVLSNLQYDELYPPVFSQPATYIRFSSTVEDCCGCPYNMTDEDDVFLKIMNEKRDPADRCT
EDQFEEVMNFFEETVRLKQPYAAVGSAPVLSFAEMQESMDATVEDYVRRLAKDVYDHWKTRRLNNGNQSL
LPSLKFETGAETDDTDPYVCFRRREVRQVRKTRGRDAQSADKLRRLRKELEDARQLVALVRQRELARKEM
LATERILFLQRAEVKDMKRKLNIKDDDEDLINQKPKKKPIEAPPMQRPAAAQLRMPPKPGAQAAEDLQLL
EDVQAEKENEIIRDIKANIAKHMKWNEGYVDFTRAPLSPSPERTVDISFRPAITTQLPTPPSSDSSENTP
DLALDMSGTVSYRDKLDEHALIMSEDANKMPSFRRRIGRGGRLLIDRRNFASRCRVELDPWKADRFKYDQ

EDSDEDLDYEMDQYDISLMQNRAIMLAKARDQAHAQAQAAQVRRLQAEQAALNNLNSGQTSGQTMGSNPG
PGAIAPTPET

Epc-N

>GzeaeEAA76124
MSSRKVRVKKLNVKTTLPVLREDQIDPNEYEALTTDNQIATGVEQAEENEYHLQTILKEAGTSNDQEIPV
PPPQESDINYDDLYPVPFHKPSSYIRFSQTVEECITCLYDMTTEDDEFLKQYNSKPPATGVLSEDDFEHI
MEVFEDTAAEQTPFAAVDNTVAAYDMMLPGLTHLNQSVSTDVLQHAKPVYEYWKSRRQEAGNKPLHPSLK
FETHQETDDTDPFVCFRRREARQTRKTRARDNKIAETLKKLRRELEDGRQLVLLAYEREMVKREVMSMDR
AIFEERARLKDMKLRLGIKGEDEDLVNQKPQKRKPVEPPVVRQPTGAHLRQPVRSDGRTLDADLVLLSDK
LAEKETELRLDIEMKVQNHRKWNQNHIDLTREPLSPVKEQGTEVKFRQAKTQYLMTPPASTSSEMEVDEI
APDAMQLDKRESSVFQFTAGSGEQSKGSQPAFRRRIGRLNRLWIDRRGMVTPPRELGEDRSDRWKYDSDS
DDEEPPVYEVDPFDTRALKFRATIPLNPYMFRGRPAVPPEAVAAAQAQAGNRVLPSPAAAAAHAHAQAHA
QAAAAAHLAAQAQAKAQAVAQQQAQAAQEQSGGPLGKVDKPGGHITRRVYYLVADEGIARSFGAGGRFSG
LNVGQIACLMARVE

Epc-N

>PchabaudiCAH77616
MTISAGAHGKAAKGKGLDIHKKILIIKSRDDIKKLIKNDNPTNEEIDEMQHLLENSELKDEKKKKKKRDI
AIPRFKICENDDYKLTKFEKPTHYIRYELYRDQVTGIKLNDGCIIHYDLLKEDEIFLESLNSYMNIHVSS
DDFCKLIDKFEKLTGYSDSKEEINLKDALNAASELKINYKSNIIKDIHTYWKAKRKKLGRPLLRMFWNNA
QNILSHYSVFRSRAKEKMTLRKHKKKNGEIIVKMQELIEDFKRLDRILRKMKQRDEKKLLLLQLNSILFD
QRKNEIKDKTYVCPMWDYFKDYKIDKIYKKFKKEKHYKNLHANNHMHYHHHHHHSILSRKISKQNDTLK
SSSFSEHMYNPRKSNHNEYKNVVLIKRRGRNNRLWIDRKSINNNEMNNMSDLNYYCDLSYVINDHINPSS
KFKKNYNDTIGNDVSSTKKHIPIKLDIKSTDLK

Epc-N

>SjaponicumAAW27112
MSKVSFRARQIDFNKPLPILKHGSELFLEISENALVNRGVPQIPSGMEKEEENEHHFLEVIQALQLRTDS
DVKIPVPEIIDKENDYKRVYPDGFCLPKQLLHIRTIVFSEEEPIEYDMDSEDEEWFRKSDLGITPEKFES
MIDRLERGCGQKVMNLEEAKYLLQDQPSLVIAVYDYWLNKRVQSRQPLLYAVRQERRDGGSNTDPYVAFR
RRSEKMQTRKTERVMNSRTKRC

Epc-N

>HsapiensCAH18228
MVIPVPEAESNIAYYESIYPGEFKMPKQLIHIQPFSLDAEQPDYDLDSEDEVFVNKLKKKMDICPLQFEE
MIDRLEKGSGQQPVSLQEAKLLLKEDDELIREVYEYWIKKRKNCRGPSLIPSVKQEKRDGSSTNDPYVAF
RRRTEKMQTRKNRKNDEASYEKMLKLRRDLSRAVTILEMIKRREKSKRELLHLTLEIMEKR

Epc-N

>AthalianaAAG10815
MSRLSFRPRPLDIHKKLPILKSFKDFEDEDNPSSITRNSQLLRISAVEVVDNEVQPPVPSKKLISEIPTP
QYLVVDTYERDYSRTFNQPASYLRARGARAELGEFVEYDLDNDDDDWLYEYNKETMILSPEMLEIVIFKL
EVLDHKARERAGVITPTLGLPVPVLLQPDAAGEALKYLSIKYGVFHAIYSYWKNKREIWQKPILRRLQPP
PPVNDTNPYNVFRPREKAHRLHARRMQQRENNAQSFEKLRQVRRNLDQAKTILEALIKREEKKRDFMASE
VSLQRIQLKYKNETELLEDSLALAGFPLSTAYRFGSSEDEFMDSDDPTTTQTCTRPSFTPHRRFTDSNLA
RAQAGSIKQEARRLGWLHKLNPNEPVMLFTKALVPDKLAAAGIIPPSDARSGRARFQGRMGRGGRIVFDR
WNPLNQAHINCGNTLYIAP

Epc-N

>PfalciparumCAD52313
MTKGGSNHKNNKIKPIDINKKLLIIKCNDDIRKLINKESPSVDEINEIKNLLENNEVKIEKKKRNIVIPR

FKICEDNNINDDEKRKDNINNDAEKNKSLSDDNNNNNNNNNNYDYDNDDEKKYSITKFEKPSHYIRYELY
KDQVTGIKLSDGSIIHYDLLKEDEIFLEGLNSYLNIHVNDESFSKLIDKFEKLTGNSDNKEEINFKEALK
AASDLKINYKSNVIKDIYTYWKNKRKKLGRPLLRMFWNNSQNSLPHYSVFRPRVKEKMTLRKHKKKNSEI
IIKMQELIQDFRRLDRILRKIKQRDEKKLLLLQLNAILFDQRINEIQDKTYVCPMWNYFKDYKIEKIYKK
FKKDKYYKNSYHHHYNNNSNNNKYYYDEKEYENYNFDANEDGYVNHNYMDEYMNDNMDEYLNNNIDAHSH
YNYRDSIYNRKINKKLKHYIIHPEQKNVLKINPSEYKNVVLIKRRGRSNRMWVDRKYVDENNINNHEINY
CDLSYAFNDFVEASLTLKKNYEEDVSNYLSGRTRIPIKLDIRKRDLKNVGDFNTSLFCNNQIHDDSLFNG
MYDELNGERKRKRRKKKEQENLNVDENYINSNILLDNMGDQFALQANNNIHLDKTKASLNDNNNNNIIIC
NNNLDIIQKNSTTNVNVDSSNMNTTNIVTCDRTSITNDHDANNTDIKLNYFINKGNKINMNEPDYNTNLL
FHKNNTEENMLNQYIQNKNNLYNINNINNINSNIKINEDNTKMVSHNILSLEHQNLKNQDFYEKYVSTNI
TNNYNNNNNNNIPYPLVSRNDKYAPCIFSKEQNNKTIFHFEDMIDPLMNKDNYLFCLSKYVYVQKKILFY
LEHLLNFDNYNFKSKKKELPNNKDMNINLKINTNDNINNDSLNNDNINKDNINCDDINNKKNDDLINNTL
SELNGTCLKSKVSHKSLKHKTAKDNNIK

Epc-N

>CelegansNP_499642
MATTSKAFRARALDSNRSMTVYWGHELPDLSECSVGNRAVTQMPSGMEKEEEQEKHLQEAIAAQQASTSG
IQLNHVIPTPKVDRVEDQRYHSTYHNKNKMHRSKYIKVHAWQALERDEPEYDYDTEDEAWLSDHTHIDPR
VLEKIFDTVESHSSETQIASEDSVINLHKSLDSSIVYEIYEYWLSKRTSAATTSGCVGVGGLIPRVRTEC
RKDGQGVINPYVAFRRRAEKMQTRKNRKNDEDSYEKILKLVHDMSKAQQLFDMTARREKQKLALIDMESE
ILAKRMEMSDFGGSPSSFNEITEKIRAAATLEVVKPPLAEINGSDEVKKRKKPRRKIADKDLISKAWLKK
NAESWNRPPSLFGQHSGNVPTVTTKPVRESLANGRFAFKRRRGCVYRAALTVYNVPTAPATVPPVQTQAA
VASSSSSKSTDMVPSNMKFFETFVRDSQDSVSRSLGFVRRRMGRGGRVVFDRMPRNRDDNDERTSTDPWA
EYCVADSSRTFRARNSSLGTEEETDDLSPKSLYFARSNRFAFNDDETEREWTSRCQQSSWRDTEVDDELK
KRETTSEKFTETTTNGSTKTHTESDDSEVERMEVDDQVDEAQITVSSSKDDGMNGNDKNEDEEDDDDDMD
VDEHQTVVGVHQHQQQQHHQQKVRHQMNGGGGGGGVVKLKPPLQELSPPLSGNGRADRAEPTPVPAKMCG
TVSDSDDWREPSGSPSESNSSTEWGGYTPQEQHAVVVANAVAVAFKEKLMNGVDDDDDQQPSPARGARDH
SIKEFFDVNGNTAGTEKVHDAVDNRSII

Epc-N

>SpombeCAB52878
MSSVSKNARAYRQRKVGIKTVMPIYFERDIPDFDEEASLQRTVPLVESGVEKEEEEEKHLQQVINEAHEA
IVRGSEKKLIIPTREAKNIGIIDKYYKKNFILPKTLIRFSLTVEECTNPEYCMDEHDTEYFLKLKQAQPS
LSKFSELDFEIVMQTFEEEINQNQPFLSMDTSQILPLSELITSFELKDVLYLKPLASQVYPYWRERRISK
GGLPIMAKAQVGDDKDDDDPYVCFRRREIRQARKTRRSDAQSYDRLRRLRQSMETSLQLLEQVYKREQKK
LQALEDDYAIFQKRCLVKKLKRTLNIKDSDELLINPKRRPIEVKPAAPVPTPAPPVKTSPHPASYRPQPT
RNVEVRPLLMLDDVQSAQITQFQIRLQQRLTKKEQLDRNWVDLLETPSTVIHTNYPDSFYRNIIPYYSGK
ETKQSHNQLSIPSSTPSTPLSDNGPTYSTPHSSLSNFNTCDSLSFSSNNSLYGYSTLLHPRNPICVRQRI
GRGGRLMLDRTRALPVHRLSKPKSRVEDRWLFDIPFDADDTIILDDESDASIMFRASLLNDDMGTQS

Epc-N

>CbriggsaeCAE72836
MATASKAFRARAFDSNRSMTVYWGHELPDLSECSVGNRAVTQMPSGMEKEEEMESHLQDAILAQQASTSG
VQVNHVIPTPKVDRVGDDRYHSTYHNRNQKRRDKYIKVHAWQALERDEPEYDYDTEDEEWLRDHQHIEPR
ILEKVFDTVENHSSETVVASEESVINFHKTLDSSIVYEVYEYWVNKRLTAASTSGCVGVGGLIPRVRTEC
RKDGQGAVNPYVAFRRRAEKMQTRKNRKNDEDSYEKILKLMHDMSKAQQLFDMTARREKQKLALLDVESE
LLTKRMELSDFGATSVFGEITEKLRATSETSKPEQNGPEEVPKKKKRIRKKVGDKDLLSKAWLRKNAECW
NRPPSLFGHNGAVPTVTTKPAREGAPDGKFAFRRRRNCGYRAPIIFTPTVIQAVPATPSASSATAATKPA
EVTPPQSSRFYETFLPVANGAMRSIGLVRRRCGRGGRVLFDRMPRNADEERLSTDPWAEYCVADNSRTFR
ARQTSLGTEEESDDQSPRSLYFARSDRCRFNDDEREREWASRYEPTDWKDIAEDEPSPPNVKKFNGTIAS
TSSHSEPVKLEEKGDNDDNSEVERMDVDGQEDEAQITVTSTKDEDVPEEEEEDGDDRKNEDDMDVDQEKK
VKKPEQSNTIQVEKFRHIMTLKPLQEHSPPLHSHSGNGRAGREEPTPLPAKMSATVSETEDWREPSGSPS
ESNSSTEWGMMPYIGQQEHQTAAAVVIAYQQKKKLMNVVVEEDESVDDEDQHHQETKDRDHAIKEFVDVN
GNNAGKPRGENVHDAVDVV

Epc-N

>TnigroviridisCAF97756
MSKLSFRARALDASKPLPVFRCEDLPDLHEYASINRAVPQMPTGMEKEEESVCFHGLKLWKYAVWRYFFI
FKRSEASLIFSAQAFTKWPPFFPTETRRISFSLDTEQPDYDLDSDDDMFVNKLKKKMEISQLQFEEMIDR
LEKGSGQQLVSLPEAKLLLKEDDELIKEVFDYWSRKRKNSKANSLIPTVKQEKRDGSSTNDPYVAFRRRT
EKMQTRKNRKNDEASYEKMLKLRRDLSRAVTILEMIKRREKSKRELLHLTLEVFEKRNVMADFGGEVMAE
VLAEQALVRPQIIPLVPLTNQYRHQDHMDHKDYKSKPDKMEVPRQKRKYEKKQKVLPLSSGAPHHQGPVV
FNAKDLNQYDFPSSDDEPFSQVAAAPTPPSPPQWRVLALTGMGSNLECLCSQLHSGSSEAEEENDPDGVY
AFRRKAGCQYYAPRQDCVGSWPWCGPGEDGLAEARFRYSLTTLTVPRRCLGMARRRVGRGGRVLLDRAHT
DYDNIFHGLDPEVLHPAHPSPPRPLTPPPTTASRSPATDKPASTSETNTSDRRCSSLDPSLCSPSSTDLS
HILLSIKSCRWRHFRPRTLPLHELDNAHPIFRKLSRGLKKRALASVAGGQPYKRPARAVPAPAPVVGVYK
GLHLASTASPSPAATAPPIAAPTPVSLHPCTTTSPPPPPVPTTALPTPPTPLSPPPPPLRSCWETTASVW
AFPPANACHSADAKHPRVRIRLKAGPRRHGQLSETQGGHCPAAGHRPQGEPRARQASTEQSIREHNGHGG
HVASARHDASPASFFFLPFFLFLSFNFRCYASLGNCDIKWQNERSKAGLSPQQFGT

Epc-N

>DdiscoideumEAL63510
MSRNGGNNTENTITTRSQRISFRPRPIDISKPLPIIRTAIDDDESTVSRLVPIIPTGMEAAEEGETHLQD
IIQASVKMRHDQLPEIPIPVVNVVAGYDQSSNPQPFSIGSTYILYHEKTEDEWDQVCEYDADDDDEEFVQ
QLNQTAMNSTNFPPTKKPILSVDKFEQIIDRFEKDFYYYGQCDQLKSEQICKGLRPQFAHMIHQHWLSKR
KQLTNNTLIRRLLKPPDREDPSPFKAFRTREIDNNKLKKARKNDAASLLKMTQLREEIEKTRIILEMIKK
REKLKKDHLNLVSQLYNKLIELAQIRQEEDDLIPNSTSSTTSTNINNISSLSSSSSSSSHKKKHKDKSS
SSSSSSSTVSINNNNNNNSSSNSNNNNNNNNNNNNNNNNNNNNNNTISNSNNSSSSSLISSSGHSNLQSI
LLNQKNPTGPVFTESHGMPIPNSKARQEGYRHFVVKPGEFPTPSLEKQYGEHSRSEKSKPPSITSDTNTT
SSSTSSSSSNNNEIIMIDGDNTNILTEKDKQINQQQKQKQQQQQLQPPQPLTKEEEIEREKRKFQMDYFN
SISIPTSKDYYQRLGDNYEDEKPASQITAGISGSSISTDSESDVESDFEYNQRIAPIPPTRPPTNGKGVP
IYPSLSQGYIFVNHNYPPVLGRVRLGLYGRTYIDYIPYNNDNSNDDDDDDDVDNNINNDNNNNNKNSSGN
NNNNNRSRGKGKNNRSGGKSKPTSISNPSLVAPQSYQFIDVQPLQRLNHNQQPTQPNSSQIITQNIENTN
NNNNNNNNKNNNNNDILSNIPLCSISSSIITTDSNHLLNIKDKGDSNLSENLNNNNNNNNDNNNDNDKNN
NNNNSDSFTLAENKINNTNLDKIDSVANSTEINNTNTNTNNNNNSNSNNKNDDNDNNNNIINNNTINSDN
DTNSEEGNNNNNNNNNNNNNNNNNNNNNNNNNNNNNNSNSNLKRKFQETINTNGTNGVTNKNINVGNNGPT
KTAKILDFEGFEVIKNGNEESNNNNNNNNNNNNNNNNNNNNKNNNNNNNNNNNNNNNNKNNNNNNNNNNN
SKKTPSKKNSTTQTPPPLNPNSSIEKYFTKKQVPSS

Epc-N

>KlactisCAG98909
MPAPAVDSSRFRHRKISVKQRLRIYKSHEIKDLEQEDVSAISSQHQQRELMEIETGVEKNEEKEEHLYKI
LQSNQLRENKKDLFIPTPDASKTWDEFDRFYQGEFKCPTSYIQFSAQLEDCCGTLYNMDEEDEIFLADLN
KSLADSVEPLTEDEFELIMANFESSIKDRQPFLSMDPESILSFADLKPTMLKNDVGDSGVKKELAKEIGM
PEDEPFLTMFDNKRPLGKREKNMETLIELFGEKIHDHWKQRKISRHGCDIFPQLKSERNNDKDDNDPYVC
FRRRELRQPRKTRRIDVQNSQKLRLLCQQLEYTKDLALTVAKRERAVLEVLENEKFVFQARAQLKTMKRK
LGIDADNEDLYSAKKQKLVSSVRTIKQQQQLLLQKQLQIQQQQQQQLQQQQAAITSDSSVKRAKSSKSSK
LHKEDSGLYADEKGSEPKKKGPKTGSNKNKEQSLSSAQEIGAQSPVANVSNVQQQQKQASSQVYVKLPNS
KIPDIVLEDVGKLLHSKEKSTRKFVEDRMRKRKQEDGDIFFNLTDDPYNPVFNLSIPDNVSPQDAPFSSV
AGSKFEVKTSYYSPNLQNYITGTANDIKVFNKEGEAVENNEYKKLEFFNPFDNEIHTHSREFPIAFRRRR
GRFNMEYIDQRKTDHNINDMLLQFIDLDGIQKQELDNDVINVYDSKLDDLSRSYYHWKYDSNYNIYGSKF
SDEPAKLNQISNDTQVVRFGTMLGSKAYEQLRDATIKYRQEQINKRKKLNSLQQQQMLQKGQQPINNAPH
SQSSSPPSHQDTRKNPGSTPNQSSPPKKHVTPNAAA

Epc-N

>AgossypiiAAS53891
MPTPSAQLDQGIISSNGGTSGVSASSTRFRHRKISVKQKLRVYKASDLKDLDQDELQQRELQEIETGVEK
NEEREVHLHKILQKNQLQLQDLYIPTPDASRVWKEFDEMYQGRFTTPASYIQFSVQLEDCCGPAYNMDER
DEAYLAELNGGESEALTEDEFELLMTNFESAIRERQPFLAMDPESLLMYEDLKPTMLKNDIGDAGLKTEL

AAELQLGDQPFVTKFDSPATLRTRNMVELIEKYGAEVYEYWKKRKVEVAGGSIIPSLKAERSTDKDDNDP
YFCFRRREVRQTRKTRRVDTQNSQKLRLLYQQLQYTKELALLVAKREKMSMDMLLRDREIFQLRCDIKTV
KRDLGIKGEDELLISQKRRKLVSNVITNKKYVSTQADAAALRRLRVAKVKDKKLLSKQLSSTDLKRQQSQ
LQKLDQQQRQQQQQQQPQINGAQCQQDGSGVSHVYVKLPTSKIPDIVLEDVDKVLSMKERNTKRFVEEKM
KKRREEDGDIFFNLTDDPYNPVFEITIPQNISPTNAPFSSIVSSNFEISRSYYTPNLQNCITGNTNSVLA
YNKEGEIVESQKYKKIEFYSPFEEKNDSHTREIPVRFRRRLGRYGVEYIDRKDVSRNPSDLLGEFMDFSL
IAEQEQSSDAVNVYDSQLDELFRLHDKWKYDSDHNAYGIKFSDEPSRLNQISNETQVIRFGTMLGTKSYE

Epc-N

>ScerevisiaeBAA09214
MPTPSNAIEINDGSHKSGRSTRRSGSRSAHDDGLDSFSKGDSGAGASAGSSNSRFRHRKISVKQHLKIYL
PNDLKHLDKDELQQREVVEIETGVEKNEEKEVHLHRILQMGSGHTKHKDYIPTPDASMTWNEYDKFYTGS
FQETTSYIKFSATVEDCCGTNYNMDERDETFLNEQVNKGSSDILTEDEFEILCSSFEHAIHERQPFLSMD
PESILSFEELKPTLIKSDMADFNLRNQLNHEINSHKTHFITQFDPVSQMNTRPLIQLIEKFGSKIYDYWR
ERKIEVNGYEIFPQLKFERPGEKEEIDPYVCFRRREVRHPRKTRRIDILNSQRLRALHQELKNAKDLALL
VAKRENVSLNWINDELKIFDQRVKIKNLKRSLNISGEDDDLINHKRKRPTIVTVEQREAELRKAELKRAA
AAAAAAKAKNNKRNNQLEDKSSRLTKQQQQQLLQQQQQQQQNALKTENGKQLANASSSSTSQPITSHVYV
KLPSSKIPDIVLEDVDALLNSKEKNARKFVQEKMEKRKIEDADVFFNLTDDPFNPVFDMSLPKNFSTSNV
PFASIASSKFQIDRSFYSSHLPEYLKGISDDIRIYDSNGRSRNKDNYNLDTKRIKKTELYDPFQENLEIH
SREYPIKFRKRVGRSNIKYVDRMPNFTTSSTKSACSLMDFVDFDSIEKEQYSREGSNDTDSINVYDSKYD
EFVRLYDKWKYDSPQNEYGIKFSDEPARLNQISNDTQVIRFGTMLGTKSYEQLREATIKYRRDYITRLKQ
KHIQHLQQQQQQQQQQQQQAQQQKQKSQNNNSNSSNSLKKLNDSLINSEAKQNSSITQKNSS

Epc-N

>YlipolyticaCAG83058
MAKAAKAAGSSARFRQRKISVKQTLAVLKQSDIPDLEEEQQRELQQIETGVEKGEEEEHHLQAAINSSIA
QSTGAKVEKIYIPTPDASQVWKEYDRFYSSSFHEPASYIRTSVTVEETSGCLYNMDDEDAEFLKTCKPPI
SEDDFEEVMHRFEVTISEKQPFVSIDVSNLLSFEEMAQHIEDGIRQVQEDPTSPEYILAQLQSSLGITVN
GTKGKNEGKAFLATFKKIGAVIYPHWRARKVERKGQSIVPHLKFEDHEKDDSDPYVCFRRRELRQVRKTR
RTDVLSIERLRRMQAEMETAKQLVEMVATREFTRKAALKAEWDVFEDRCAIKTLKRELGIKGEDEDLVAQ
KKRKVEPKKEEKAEKASTPVRGGKAAGSAASAQAAAAQAAAAGSGSPSVSSTHVPPNVSIPPSKIPNMDL
ITIAQVVRDKDEAIAKAVREKLRLRADADRDWHNLTNSGYIPYCEYLNAEVSSSGEPPVPQYSSINEMAY
FEKHNASHRYTTKSDFNKDLASMVGNKPFADAQVYGAIVGDDGELRLSDATSTSSPVDRVIPRSSFMSMR
KRVGRGGRMWMDRRGLQRNTVLKPSSLAKNSLDDTAESEADEVAMERLADRQKYDRETEPTRQMSSYDKD
PSQLNGISSDTQSIRFGSMLLSKAYENYREVFQQRQQQLMMLQQQILQQQQQQQMRNRQQSHPPGDPGAG
LGGGQGAGGGAGGSRNNSPAPGTNGPQSKMHNAAPMGYNKQGMTPSQHQQYQQMQQQQQQQQQQQQQRKM
GVAPMNAASAAAAMAAQPRRSSGSPDGQRFNGLPNGGAMANGVLPNGMSQRMMPGGDMKQKSELAKVDA

Epc-N

>CalbicansEAK99771
MAAAPPPPAKNQGKAKQHVTGARFRQRKISVKQPLTIYKQRDLPTLDSNELEPSQVHHLNSNASSSSTQQ
PRDLHAVETGVDKNEEEEVHLQQVINAAQKALLGSKKEEKSSDMYIPTPDASRIWPEAHKYYKDQKFKQP
ETYIKFSATVEDTVGVEYNMDEVDEKFYRETLCKYYPKKKNKSDENNRKCTELEFETICDKLEKTIEARQ
PFLSMDPSNILSYEELSSYIVDQFKSAVKTSNPYIVTNGGNLEYISTTALKERLSKEIKYEPFVTIFDKN
QMSTSAVRPIPKLFELFGRPVYDHWKERKIERKGKTIQPTLKFEDPNSNEKENDNDPYICFRRREFRQAR
KTRRADTIGAERIRSMQKSLHRARDLIMSVSEREILKLDNFQAEHELFKARCATKACKRELNIKGDEYLF
FPHKKKKIVRTEDEEREKKREKKKQDQELALKQQQALQQQQQQPPQPPQQAPSKQDGTSTSQPYVKLPPA
KVPDMDLVTVSLVLKEKNETIKRAVLEKLRKRKEHDKGFINLTDDPYQPFFDISTNRAEELSHIPYSSIA
ATHYHQFNTSNYMNDQLKKLLEEKKPLPGVKTFLGSNGELVPSKAFPHLSSLLEEKYKATSGYIERLLQS
VETQDFSSYTNGFKDVEPKETNEPVMAFPQRIRRRVGRAGRVFLDHQQEYPQPNFQQDTDRVGGIPDVYC
KEDAIKRLQSKWKFDTEYKTTEPFSLDPSKLNGISPSTQSIRFGSMLLNRTRK

Epc-N

>CneoformansAAW47131
MVAPGRMVTSSRRIGRVTNKTKLIIYRGSDKVDTSAAETVLWDQEAGGAGKDSNKHQHIGATGVESGELL
EHHLQAALSSASLLHSSNKPSSPKSVKEAPAAALNYHIPTPDATGLVSDTVFSQLYQRTKYVEPYNFIRF
SDTVEESSCGWGGLGYCMDDADERWLNDFNSKAEGSSGDVKSDKEQGRGMRVKGKDREKEKGDAPAPLVI
SEDMFEYIMGVFEKYTEENAPMLHTDLSLLPPFSAVENMFSTPISPAFLPSNEIPKELGDLKACARMARN
VYPHWKSRREQRQGKSILPQLNYDETNDNDPYVCFRRRDIRATRKTRRTDNFSIEQFQKLQFELRSAHAL
ADRVLTREREKKSLYEAEKELWEARWKFFETKRRWPSLGMTSDEEHKITGRPTIVPPIQIPSLSGQTPLT
SGQSSSHMRKRTDKDREERAQRERYDAQRNAERSGILSGRSNAPDALKERLQALQQKTEEMLARKKEQDA
HWDDSIDSPYQPLPPSNSVHAFRSLFVLDPCRAQCKDSETGNEILHPESFRIRRGRGGIVRLDRRTSIYS
HRRGIQPTSPSEYPTWLFPDIAPRRSEKKRPRSIDEVEEEMQEQSPKVMRKDLNETWRYDVDRGGAVGVG
MGLEEDYDRVIIDDLEAKYIRHRISLLQESDCAKLRPDNYILDQTREALDAAADAKPPPAPIFQKPPAPQ
PNPQLLAAHLQQQQMLAQQQQMEQFQRFQLMAQQQAMAQAQAQAQAQAQAQAQAQAQAQVQAQGQGHPQA
HLQTHPQGVPQPNGVNSPMPNGQQMLPPSDGVKQLKLPPHAVARLGAAMANANANANGGLHVLQQQQQQH
AQTSQQ

Epc-N

>E. uhligi AAF13352
KLDKGLSKTRHVTRPRTVDHNRKLLVSIEKQVSERDIIVPRVPNIETPVVYYYDKYFMPNIRPYVRPGHY
HRYPDISTIDYDTVEEYEATDEDQEYYATLQSKYTHPAFTLNAFERCIEAFERDTEKGQVIPQERAVCLI
KENPRFDSWMRPEPIEPLLEEIYQYWRGRRDEDKKPLLRRFWKMEGIEDMHLKLVFQPRNSNRERMRLRN
SRKNDSESWEKMKNLHLQFTCLLQITNSVCHREQLKKYSLIGRFRSFQCERADKGIALEDKPSFESSVKA
FLDDKLLDESRIFRNSIQIQEPTPIIPIRPKVGSAEPRAPAPQPIKRLHPDRKPLLTFEIAKLCLLVIAE
GERQNTWPIAVAKDYSAEARDGIGEGEIRRRKKFRDRARFGRGGRFLQLDRYLDSDYDHTWGRYQDQQGK
WHTPGYEDIEQIYEDDDTEDIKRLAITIQQSLKQWVKQKKNA

Epc-N

>U. maydis EAK82052
MARSAAPVPAATTSSRVRNKKLSYRQKLCVQRGSHLLNAATSVVAGPEPEFEGQEHTQDSNLGVDANEIS
EHHLQAALSSNQLATAEGNSTTETTKAAYSIPVPDAHGTLSNTEFSKLYPSGVYSDPVTYIRWSDTVEDC
IKGPSYNMDEDDQDWLETRNAKAQEALTTAIRNTKTGPLAGSAGKGKAKEKLREEAIEKLVAENQGSYRL
LSEEQFEMIMTVFEQVTSEQVPFLHLDVTKIPTCEELLSYFEPSSSISALAQPDLPPLSWEKTVSASLGS
SSQARRPPVAGAASSGSSSIPTTSTEWSPRNPYKNLTDLKNLATVVYPHWKSRREEREGRAVTPYLNFDE
TNDNDPYVCFRRREVKQIRKTRKTDALHIEKMIRLRAELEHAVALTELVAQREKTKRASLRQRRECWENV
KDLMDIKRQWGIVGPQQGLEDEELISGERRDPTQASSGPVKKKRKVEEAATTIKLAGRKSRGGEADGTSG
GAAAAAAGGVSGMGSAILERVHAVQAYIERECLRKADSDLGWEEGSDAAFQPIPAPAPLRAFRPIHAESS
DEPLPFSPLATRAGRPPSFRRRVGRGGRVFLDRRLPVPSPVPTSLSDWPRLGRTKTDLPSVEARDLRSTQ
PAYSSAPISSTLPDLGLANPPPSNRKALTGPFAFSPDLRPQLLASTTHPAVQSILNQGNDAFCGPFARSG
PLAAASSTNSLEEISGSQHSDASSTSSDSSDRSRLSGTSVGVVSTQATEVGDIDMLGLEESHDKRETEQQ
GDALDSDGSDDTLDPERELQLWTRLEEQWRYDDELGRFAGLGLTALGGMEDDDEAVLDDFDQKFMRYRMT
LLEEADLLKLSTDLTNLQQAQAAAEAPAPKPAGYVEQPATSSSSSAANTSAKASGAPAAVAATATLPGIP
SAANLPLPSAARTAAATGGGGSQSALQAQQLQAQQQQLQMAILQQQQQQQQQQQQQQQQQAAAAAQAQAQ
AQAAAQTQAQQAAILQQQQNQQHQKQQQLQQQNQALSQTQAPQPQTQQVSGQQQLPQPFCGQIMSLSHSN
NQQAQLAAFAAAQQQLQAAQQAAAQQQQQQQQQQQQQQQQQQQQRRMSGVSQQQPQQQQIGLPVQMQMQM
PIQMQMQLQMQMAAAAAQGQTHLLGQSMPFDATSIQAQMQQAQQHLARSMPQQNMSLSSPMQAAAFTVPN
GAAPFGMNLSLNMNGNAGSPPMQHARVSSQPQQQIARTSASPVSTAANGQNTVRTNPSPVMQPGLIMPQQ
QAQQVPQQQHNQTGALNLNLSLAAAQMKSGTAALQQFQALQQQAQMMQGQQGNATSAQLMAMKAALAQNA
NLNLRLPPNRALQIAQQAAQAAFGAQQQQQQQQQQQQQQQQQQQQQHQQQQMSGNASNQGSPAMSRASTA
GSPRFAANGIAGAMAGSSPAQNGTSATTNATNSPSPQISAASRAAIAAATNAANAKMMAAQSPVSYHASP
VQANLNLHAVQQQQLKNAAAAAAVAGGAAAGSPHLATRGSPQTQQG

Epc-N

>D. hansenii XP_460100
MAIHPSKGTGGSKQSNSGARFRQRKISVKQPLTIYKQSDLPTLNASNDLEPSQIHHLNLNANQQQRDIHA
IETGVDKNEEDEVHLQQVINAAQKVLLGSQNEDGDKKKDDSDKKTDALVYIPTPDASRIWTDASKYYNDK
SFREPETYIKFSATVEDTVGVEFNMDEIDEEFLKNKLWKNYPKVKSPKVKSEKLDDTNKENDNARKCSEV

EFEIICDKLEKIIEEKQPFLSMDPSNILSFKELSAYIIEEFNNSNKDKPYVQLGSNLKYISTTALKEKLS
KELSFEPFVTLFDKSLLDQTSTNIVRPIPKLLELFGEPVYEHWKYRKIERKGKQIHPALKFEDPSANEKD
NDNDPYICFRRREFRQARKTRRADTLGAERIRLLQKSMHRARDLVMSVCRRELIKLENWETDHAIFKLRS
DAKNLKRVVGVKGDDFLFYPHKRKKIIKVKEEDEDKESSKIKRDKRSRFDSSREGSATSMPGSATIGTNA
INKDRLANGQVHHTQEASSSSQPYVKLPPSKIPDMGLVTVSLVLKEKNETIKRAVLEKLRKRKEQDKDFI
NVTDDPYQPFFNIATNTKFKNNELKHIPYSSIAATSFHEINTTNYISEKLKNLLEEGKKPLPGTKTFRGS
NGELIPSKPFPHLLALIQDRIDNSQFNSVSYIAQLLSNIENNNFSAYNNGYGQQQQHQHQETNRDKTKLS
DPIFRLRKRVGRFNRNFVDRRGLMKRPNDVIDDFLKFDDEGVNDDCDQMDVDSELISKNNVPNVYDSRVD
EIKRLDSRWQFDNDLTEYDKGLQSPFSLDPSRLNCISDDTQSIRFGSMLLSKSYDLLRDSVHQRQQALVQ
QARMRTLQQQQRNNKQQAAGQSSGSSSASLGSNTNSNSSISGQADQGQTNLTNSGITRQGGANVNGSQTS
TTNNTRSSVSGGSMNPKLPTQSLQRSNTNSPLLASQPQGYSQQQKFNKIPPTSQSQSQSPTHAAGQLQTS
KMYNKHGSNITPSNLKGPKFTPANNNQIGGSLPNRK

Epc-N

>P. vivax CAB96695
MAGDHQDPGYISYKDYVNAKHEFKRTMNNRYPDNERFEKIINKINNESHKWNLKNKTFTTLHNVLANDLA
FYGGMKHHYCRFINYWLNKEVQNINNHFDKSYFPIFQKFSDEFSIIKTNKNDQRCNNYIFNLSHKTINIM
DILYGLYDEYDKIKSHREDSNYGSCDTFLSWASNHNYAIDKYYENTNLYQKFEEIKKLIDNLKESSSSSC
IKSIYLHKPQVVKLREEEEARREAAEKQKRALEEAIREREEAEKRQHEQELKAEKELLQRQANNTVGDTA
SGEGTFQHTDDRSDLRAPIISELDQNAQRFGYPERSESSRGLLLDNGHTREGQFIYTNEDTRQQGEGVYE
QPGTDKTRSGMFGGSSGFPRYITEVFGSVDPVPVVGVSGGMGALFLLFRYTPLGTFFRGGRGRAHRIPRS
FNGQFLGAFPDINEYNGGYIGYGPMDIPYGAE

Epc-N

Epc-N SUBFAMILY III

>PtroglodytesXP_525628
MLGKSCNCGLWAGPLLLGERCCGLRSLLVWSERGLVCGGRDAGAQEGSSGCLSACYCCSLPGPGDSLRPG
CVVLDTLERGNHYQMRRKGRCHRGSAARHPSSPCSVKHSPTRETLTYAQAQRMVEIEIEGRLHRISIFDP
LEIILEDDLTAQEMSECNSNKENSERPPVCLRTKRHKNNRVKKKNEALPSAHGTPASASALPEPKVRIVE
YSPPSAPRRPPVYYKFIEKSAEELDNEVEYDMDEEDYAWLEIVNEKRKGDCVPAVSQSMFEFLMDRFEKE
SHCENQKQGEQQSLIDEDAVCCICMDGECQNSNVILFCDMCNLAVHQECYGVPYIPEGQWLCRHCLQSRA
RPADCVLCPNKGGAFKKTDDDRWGHVVCALWIPEVGFANTVFIEPIDGVRNIPPARWKLTCYLCKQKGVG
ACIQCHKANCYTAFHVTCAQKAGLYMKMEPVKELTGGGTTFSVRKTAYCDVHTPPGCTRRPLNIYGDVEM
KNGVCRKESSVKTVRSTSKVRKKAKKAKKAVAEPCAVLPTVMVLACEVLGASVYSGRHLVSRMVLACEVL
GASVYSGRHLVSRMVLACEVLGVSVYSGRHLVSRMVLACEVLGASVYSGQYLVSRVVLVCEVLAGHEVTR
SSPAQPAEEEPCGAVIRAQRPSAPADALSSLWLPSVGCPWRAVLRGQAFGVLSMQTALGVVGRIVLALNR
IANQVAIQRKKQFVERAHSYWLLKRLSRNGAPLLRRLQSSLQSQRSSQQPPGLPERTARFGGLLRGRVRK
WLSGGKLWGLEALVSAPAMLGKGREWRHWSRPPRCWGKEESGGIGLGPCDVGEWKRGFFGRKLRVQILGL
ISGLDRTPDKPQRSCEGQIPWKVGMDVAKQGTSSCGNHHFDSHKLGHEGQNPCSSRTRGPVGRGHGDPGS
WGWPGAWWSQAREGGGLQRRGDPLEPASWAPSASSLPVRPHVLLCAEYVCKERTFPQPGQSSSSGRGGSF
AKTPGKEQKELPGLEPQQSSRRGEPGNLPLCERAVGRDCASAFSWPGMATAWPQGEQGRQLSFIKGEQRF
VCMLVASHCVSCSRGPPFLSWERILQLSQCLSCGMLGAAAHIVLAVLRVWPLPVPRTCWRHWAQWQELGA
VARRPGVGHSGSDFLLITLVWMLAGLRLAACAVWRTLLSTEKGAGAVNGNGTLAVGKRVSGWRRCAGQGS
SRSLHSQLSWRWVLEGPPFCPGPAFVGLSGSVKAPPPPACGGLCPLWRVEPVHRGQPPAHLGLAVPSVPS
IPSVSSVPSVPVLSWGSLVRRENDEEMKAAKEKLKYWQRLRHDLERARLLIELLRKREKLKREQVGLGLS
RVECTTGHGAWVGRWQLLQRVKVEQVAMELRLTPLTVLLRSVLDQLQDKDPARIFAQPVSLKEVGVCGAR
LSCRMCDCGFVLPFDRETCDWLEYIPCVPDYLDHIKHPMDFATMRKRLEAQGYKNLHEFEEDFDLIIDNC
MKYNARDTVFYRAAVRLRDQGGVVLRQARREVDSIGLEEASGMHLPERPAAAPRRPFSWEDVDRLLDPAN
RAHLGLEEQLRELLDMLDLTCAMKSSGSRSKRAKLLKKEIALLRNKLSQQHSQPPPTGPGLEGFEEDGAA
LGPEAGEEVDLDTLRLHEADSRVRPSCHNIGSWFSAFLPFPCTSKSEQQTVEIRHRIVVQFSFKTKTFIS
YGRQLWALEVHVWRSPCRARALSTWSTVALRKGRMRVPRGDKSPPKLEPSDALPLPSNSETNSEPPTLKP
VELNPEQSKLFKRVTFDNESHSACTQSALVSGRPPEPTRASSGDVPAAAASAVAEPASDVNRRTSVLFCK
SKSSFRGWRLFCSQGKGRGAHAETPRWRRSPQESAWTQPPLVKDSDLVFQAPRKFCDESGQGAVHWVLEV
GSVQLWASPLHAELGVRLTLHFLGVSRLSAAEVAPPDRALPQLWAPTVDSFAGWRPRPQQPLRLVHQMSL
VEEHVSPPGHCLTLEWSVLQASRGDSGVEQSYCRPSVSVAHGFCSCDGPAPESGPDSWLPSSATARSPAV
NRHLKRPSTRHRFGDPCCKSLCARAGACESVGVSLSTRRRCGDPCRESPCARAGACIVTERVPARWALGL
SLTVPSGIVTEQVLSRHLVWALGLSLTIPAALSRSGSQQDGPWVEKCTKTALLFTMVKVKLKTGDSGLSF
ATAWGPQGSGFQGAGSAHLLHTAKLRMSRRSRGSSLGAGGRGGLVSRAVGLCSHGSMPSGVSAATDQGLV
FLHLWPGGFVVSTWVEGWEPETRGDYGPSVRPAGLRLPRWALGLLFPVGRGGVSAAVAFSLSEDPLGTSR
DSCRCAVDDLPCVLIPFLAPLHAVVHTELGPSWLLSGLCMAWLCWCRHSCGCSRARALRLAPLGSLVKAA
LVIVFPQVAIARPTSLTNGFGGARSEQEPGGGLGRKATPRRRCASESSISSSNSPLCDSRFTSSRPAHPQ
ACLAVGVSGRMRGQLGGGQLTGRSLSEAEGRGGAPDSLCSSSRSFNAPKCGRGKPALVRRHTLEDRSELI
SCIENGNYAKAARIAAAAQGTDCRVGALNFRPLGEGEAPGFGVAQPSLWKAAMLRGGIRTEIPQIIDPKM
PRVPGHHNGVTIPAPPLDVLKIGEHMQTKSDEKLFLVLFFDNKRSWQWLPKSKMVPLGIDETIDKLKMME
GRNSSIRKAVRIAFDRAMNHLSRVHGEPTSDLSDID

Canonical Epc-N
PHD 6.3e-10
Bromodomain 1.5e-17
PWWP 0.0002

>CfamiliarisXP_538319
MGLLSVVLAWPRGPVVRHGSQYQMRRKGRCHRGAAARHPSSPCSTKHSPTRETLTYAQAQRMVEIEIEGR
LHRISIFDPLEIILEDDLTAQELSECNSNKENSERPPVCLRTKRHKNNRVKKKHEALPSAHGPLASASAL
PEPKVRIVEYSPPSAPRRPPVYYKFIEKSAEELDNEVEYDMDEEDYAWLEIVNEKRKGDCVSAVSQSVFE
FLMDRFEKESYCENQKQGEQQSLIDEDAVCCICMDGECQNSNVILFCDMCNLAVHQECYGVPYIPEGQWL
CRHCLQSRARPADCVLCPNKGGAFKKTDDDRWGHVVCALWIPEVGFANTVFIEPIDGVRNIPPARWKLTC
YLCKQKGVGACIQCHKANCYTAFHVTCAQKAGLYMKMEPVKELTGGATTFSVRKTAYCDVHTPPGCTRRP
LNIYGEVEMKNGVCRKDSSVKAVRSTSKVRKKAKKAKKTLSEPCAALPPVCAPYIPPQRLNRIANQVAIQ
RKKQFVERAHSYWLLKRLSRNGAPLLRRLQSSLQSQRSTQQRENDEEIQAAKEKLKYWQRLRHDLERARL
LIELLRKREKLKREQRAVVSVPTECRIWQVKVEQMALELRLTPLTVLLRSVLDQLQEKDPARIFAQPVSL
KEVSVHPLGTGLPGGLGNNCMKYNAKDTVFYRAAVRLRDQGGVVLRQARRQADSIGFEEASGMHLPERPA
AAPRRPFSWDDVDRLLNPANRAHMVLEEQLRELLDKLDLTCAMKSSGSRSKRAKLLKKEIAVLRNKLSQQ

HSQPPPAESGIGSFEEEGAQLGQETGEEEPTLASSGDAPAAAASAVAEPASDLGTKTFLSVVLPRLETLL
QPRKRSRSTCGDSEVEEGSPGKRLDTARGGSAQVLDGCPLPLPELAAAGRDASSARPDPVGARPKLHPGG
APGIPLAGLTNGFGGARSEQELGGTPGRRAAPRRRCASESSISSSSSPLCDSSFSAPKCGRGKPALVRRH
TLEDRSELISCIENGNYAKAARIAAEVGQNSMWISTDAAASVLEPLKVVWAKCSGYPSYPALVSREHLLR
DMHAGVRMLGKLLRKPPQIIDPKMPRVPGHHNGVTIPAPPLDVLKIGEHMQTKADEKLFLVLFFDNKRSW
QWLPKSKMVPLGMDETIDKLKMMEGRNSSIRKAVRVAFDRAMSHLSRVHGEPSSDLSDID

Canonical Epc-N
PHD 6.3e-10
Bromodomain 0.00015
PWWP 5e-15

>CfamiliarisXP_541782 (misannotation?)
MSLSGASERSVPATKIEITVSCRNLLDLDTFSKSDPMVVLYTQSRASQEWREFGRTEVIDNTLNPDFVRK
FVLDYFFEEKQNLRFDVYNVDSKTNISKPDFLGQAFLALGEVIGGQGSRVERPLTGVPGKKCGTILLTAE
ELSNCRDIATMQLCANKLDKKDFFGKSDPFLVFYRSNEDGTFTICHKTEVVKNTLNPVWQPFSIPVRALC
NGDYDRTVKIDVYDWDRDGSHDFIGEFTTSYRELSKAQNQFTVYEVLNPRKKCKKKKYVNSGTVTLLSFS
VDSEFTFVDYIKGGTQLNFTVAIDFTASNGNPLQPTSLHYMSPYQLSAYAMALKAVGEIIQDYDSDKLFP
AYGFGAKLPPEGRISHQFPLNNNDEDPNCAGIDGVLESYFQSLRTVQLYGPTYFAPVINQVARAAAKISD
GSQYYVLLIITDGVISDMTQTKEAIVSASSLPMSIIIVGVGPAMFEAMEELDGDDVRVSSRGRYAERDIV
QPLFSLLGPTLQMSPPILRKLRPVEWFLLRQTRPTPTQNSEPFLRLQPASLWLLALHPVPPRGSSPASPP
FSETKSGPTAAARPAEGSADWPQTARLLRMPNTRFSLQKLNSKMEVLERPCGVPRGSSEFQRMASRGSAA
GRGSVAKVQLDSAEAPMSGPEARPPSGFRNGRRPTELRHQGAGGTGSGRGQRGNPGEPGAERGAAGSRGG
EFEFWFPPLVPLPPSPSGTSAGGSVLLWEPGRQSRRAHQGPIIEREELACSSDWFVFKDTAAGLEKLGQY
LTKLGFLGAGAICCHYSPRNTGEEEEGKALRTVVELNVPRLCDSMGVDFDVKTFCHNLRATKPPYECPVE
TCRKVYKSYSGIEYHLYHYDHDNPPPPQQTPLRKHKKKGRQSRPANKQSPSPSEVSQSPGREVMSYAQAQ
RMVEVDLHGRVHRISIFDNLDVVSEDEEAPEEAPENGSNKENTETPAATPKSGKHKNKEKRKDSNHHHHH
NASVSTTPKLPEVVYRELEQDTPDAPPRPTSYYRYIEKSAEELDEEVEYDMDEEDYIWLDIMNERRKTEG
VSPIPQEIFEYLMDRLEKESYFESHNKGDPNALVDEDAVCCICNDGECQNSNVILFCDMCNLAVHQECYG
VPYIPEGQWLCRRCLQSPSRAVDCALCPNKGGAFKQTDDGRWAHVVCALWIPEVCFANTVFLEPIDSIEH
IPPARWKLTCYICKQRGSGACIQCHKANCYTAFHVTCAQQAGLYMKMEPVRETGANGTSFSVRKTAYCDI
HTPPGSARRLPALSHSEGEEDEDEEEDEGKSWSSEKVKKAKAKSRIKMKKARKILAEKRAAAPVVSVPCI
PPHRLSKITNRLTIQRKSQFMQRLHSYWTLKRQSRNGVPLLRRLQTHLQSQRSCDQVGRDSEDKNWALKE
QLKSWQRLRHDLERARLLVELIRKREKLKRETIKVQQIAMEMQLTPFLILLRKTLEQLQEKDTGNIFSEP
VPLSEVTELDEVPDYLDHIKKPMDFFTMKQNLEAYRYLNFDDFEEDFNLIVSNCLKYNAKDTIFYRAAVR
LREQGGAVLRQARRQAEKMGIDFETGMHIPHSLAGDEAPQHTEDAAEEERLVLLENQKHLPVEEQLKLLL
ERLDEVNASKQSVGRSRRAKMIKKEMTALRRKLAHQRETGRDGPDRHGPTSRSSLTPHPAACDKDGQTDS
AAEESSSQETSKGLGPNMSSTPAHEVGRRTSVLFSKKNPKTAGPPKRPGRPPKNRESQMTPSHGGSPVGP
PQLPIMGSLRQRKRGRSPRPSSSSDSDSDKSTEDPPMDLPANGFSGGNQPVKKSFLVYRNDCSLPRSSSD
SESSSSSSSSAASDRTSTTPSKQGRGKPSFSRGTFPEDSSEDTSGTENEAYSVGTGRGVGHSSKYPHPRP
GMLGAQCQGLASPLAADPPPLSHSCEVVRKSLGRGAGWLSEDEDSPLDALDLVWAKCRGYPSYPALIIDP
KMPREGMFHHGVPIPVPPLEVLKLGEQMTQEAREHLYLVLFFDNKRTWQWLPRTKLVPLGVNQDLDKEKM
LEGRKSNIRKSVQIAYHRALQHRSKVQGEQSSETSDSD

C2 1.1e-11
C2 8e-18
Copine 8.5e-93
Canonical Epc-N
PHD 5.4e-12
Bromodomain 9.3e-31
AT hooks
PWWP 4.6e-40

>HsapiensAAH47508
MRRKGRCHRGSAARHPSSPCSVKHSPTRETLTYAQAQGMVEIEIEGRLHRISIFDPLEIILEDDLTAQEM
SECNSNKENSERPPVCLRTKRHKNNRVKKKNEALPSAHGTPASASALPEPKVRIVEYSPPSAPRRPPVYY
KFIEKSAEELDNEVEYDMDEEDYAWLEIVNEKRKGDCVPAVSQSMFEFLMDRFEKESHCENQKQGEQQSL
IDEDAVCCICMDGECQNSNVILFCDMCNLAVHQECYGVPYIPEGQWLCRHCLQSRARPADCVLCHNKGGA

FKKTDDDRWGHVVCALWIPEVGFANTVFIEPIDGVRNIPPARWKLTCYLCKQKGVGACIQCHKANCYTAF
HVTCAQKAGLYMKMEPVKELTGGGTTFSVRKTAYCDVHTPPGCTRRPLNIYGDVEMKNGVCRKESSVKTV
RSTSKVRKKAKKAKKALAEPCAVLPTVCAPYIPPQRLNRIANQVAIQRKKQFVERAHSYWLLKRLSRNGA
PLLRRLQSSLQSQRSSQQRENDEEMKAAKEKLKYWQRLRHDLERARLLIELLRKREKLKREQVKVEQVAM
ELRLTPLTVLLRSVLDQLQDKDPARIFAQPVSLKEVPDYLDHIKHPMDFATMRKRLEAQGYKNLHEFEED
FDLIIDNCMKYNARDTVFYRAAVRLRDQGGVVLRQARREVDSIGLEEASGMHLPERPAAAPRRPFSWEDV
DRLLDPANRAHLGLEEQLRELLDMLDLTCAMKSSGSRSKRAKLLKKEIALLRNKLSQQHSQPLPTGPGLE
GFEEDGAALGPEAGEEGDKSPPKLEPSDALPLPSNSETNSEPPTLKPVELNPEQSKLFKRVTFDNESHSA
CTQSALVSGRPPEPTRASSGDVPAAAASAVAEPASDVNRRTSVLFCKSKSVSPPKSAKNTETQPTSPQLG
TKTFLSVVLPRLETLLQPRKRSRSTCGDSEVEEESPGKRLDAGLTNGFGGARSEQEPGGGLGRKATPRRR
CASESSISSSNSPLCDSSFNAPKCGRGKPALVRRHTLEDRSELISCIENGNYAKAARIAAEVGQSSMWIS
TDAASVLEPLKVVWAKCSGYPSYPALIIDPKMPRVPGHHNGVTIPAPPLDVLKIGEHMQTKSDEKLFLV
LFFDNKRSWQWLPKSKMVPLGIDETIDKLKMMEGRNSSIRKAVRIAFDRAMNHLSRVHGEPTSDLSDID

Canonical Epc-N
PHD 6.3e-10
Bromodomain 5.4e-32
PWWP 1.1e-19

>MmusculusXP_128275
MFGGGSGGSALSAGGEAGESGRRGPEAVRPGGRGSRRDREGRADVRRREPARPGTPEIVERGLVSPEQAR
SSATTPWMSAGNAEKRARVRGFLGPLGEKQLSCALNLQKTQLLMVPCSCNHCQMRRKGRCHRGSAARHPS
SPCSIKHSPTRETLTYAQAQRMVEIEIEGRLHRISIFDPLEIILEDDLTAQEMSECNSNKENSERPPVCL
RTKRHKNNRVKKKNEVLPSTHGTPASASALPEPKVRIVEYSPPSAPRRPPVYYKFIEKSAEELDNEVEYD
MDEEDYAWLEIINEKRKGDCVSAVSQNMFEFLMDRFEKESYCENQKQGEQQSLIDEDAVCCICMDGECQN
SNVILFCDMCNLAVHQECYGVPYIPEGQWLCRHCLQSRARPADCVLCPNKGGAFKKTDDDRWGHVVCALW
IPEVGFANTVFIEPIDGVRNIPPARWKLTCYLCKQKGVGACIQCHKANCYTAFHVTCAQKAGLYMKMEPV
KELTGGSATFSVRKTAYCDVHTPPGCTRRPLNIYGDVEMKNGVCRKESSVKTVRSTSKVRKKAKKAKKTL
AEPCAVLPTVCAPYIPPQRLNRIANQVAIQRKKQFVERAHSYWLLKRLSRNGAPLLRRLQSSLQSQRNTQ
QRENDEEMKAAKEKLKYWQRLRHDLERARLLIELLRKREKLKREQVKVEQMAMELRLTPLTVLLRSVLEQ
LQEKDPAKIFAQPVSLKEVPDYLDHIKHPMDFATMRKRLEAQGYKNLHAFEEDFNLIVDNCMKYNAKDTV
FYRAAVRLRDQGGVVLRQARREVESIGLEEASGMHLPERPIAAPRRPFSWEEVDRLLDPANRAHMSLEEQ
LRELLDKLDLTCSMKSSGSRSKRAKLLKKEIALLRNKLSQQHSQAPPTGAGTGGFEDEAAPLAPDTAEEG
ANSPPKLEPSDALPLPSNSETNSEPPTLNPVELHPEQSKLFKRVTFDNESHSTCTQSALVSGHPPEPTLA
SSGDVPAAAASAVAEPSSDVNRRTSVLFCKSKSVSPPKSAKNTETQPTSPQLGTKTFLSVVLPRLETLLQ
PRKRSRSTCGDSEVEEESPGKRLDTGLTNGFGGARSEQEPGGGPGRKAAPRRRCASESSICSSNSPLCDS
SFSTPKCGRGKPALVRRHTLEDRSELISCIENGNYAKAARIAAEVGQSNMWISTDAAASVLEPLKVVWAK
CSGYPSYPALIIDPKMPRVPGHHNGVTIPAPPLDVLKIGEHMQTKSEEKLFLVLFFDNKRSWQWLPKSKM
VPLGVDETIDKLKMMEGRNSSIRKAVRIAFDRAMNHLSRVHGEPASDLSDID

Canonical Epc-N
PHD 6.3e-10
Bromodomain 2.2e-32
PWWP 1e-19

>PtroglodytesXP_516261
MSPDSRWPASWGNSVSFGAARASGRRGALFHLLHFHKGETPPHPESGLRALPGPSPIPPAPAPYFLQPPE
LQGQMQPPFSLGPPHGLSGLSSRLPRLCLCCRVPRGDCRFAVLLWEPFTRDPAWKEESLFVLRCDSMGVD
FDVKTFCHNLRATKPPYECPVETCRKVYKSYSGIEYHLYHYDHDNPPPPQQTPLRKHKKKGRQSRPANKQ
SPSPSEVSQSPGREVMSYAQAQRMVEVDLHGRVHRISIFDNLDVVSEDEEAPEEAPENGSNKENTETPAA
TPKSGKHKNKEKRKDSNHHHHHNVSASTTPKLPEVVYRELEQDTPDAPPRPTSYYRYIEKSAEELDEEVE
YDMDEEDYIWLDIMNDRRKTEGVSPIPQEIFEYLMDRLEKESYFESHNKGDPNALVDEDAVCCICNDGEC
QNSNVILFCDMCNLAVHQECYGVPYIPEGQWLCRRCLQSPSRAVDCALCPNKGGAFKQTDDGRWAHVVCA
LWIPEVCFANTVFLEPIDSIEHIPPARWKLTCYICKQRGSGACIQCHKANCYTAFHVTCAQQAGLYMKME
PVRETGANGTSFSVRKTAYCDIHTPPGSARRLPALSHSEGEEDEDEEEDEGKGWSSEKVKKAKAKSRIKM
KKARKILAEKRAAAPVVSVPCIPPHRLSKITNRLTIQRKSQFMQRLHSYWTLKRQSRNGVPLLRRLQTHL
QSQRNCDQVGRDSEDKNWALKEQLKSWQRLRHDLERARLLVELIRKREKLKRETFCWKQNVTGAYEQNPQ
LCDIGWERRRKHLPVEEQLKLLLERLDEVNASKQSVGRSRRAKMIKKEMTALRRKLAHQRETGRDGPERH

GPSSRGSLTPHPAACDKDGQTDSAAEESSSQETSKGLGPNMSSTPAHEVGRRTSVLFSKKNPKTAGPPKR
PGRPPKNRESQMTPSHGGSPVGPPQLPIMSSLRQRKRGRSPRPSSSSDSDSDKSTEDPPMDLPANGFSGG
NQPVKKSFLVYRNDCSLPRSSSDSESSSSSSSSAASDRTSTTPSKQGRGKPSFSRGTFPEDSSEDTSGTE
NEAYSVGTGRGVGHSSKFPCPRPGMLGTQCQGLASPPAADPPPLPHSCEVVRKSLGRGAGWLSEDEDSPL
DALDLVWAKCRGYPSYPALIIDPKMPREGMFHHGVPIPVPPLEVLKLGEQMTQEAREHLYLVLFFDNKRT
WQWLPRTKLVPLGVNQDLDKEKMLEGRKSNIRKSVQIAYHRALQHRSKVQGEQSSETSDSD

Canonical Epc-N
PHD 5.4e-12
AT hooks
PWWP 4.6e-40

>GgallusXP_415977
MLAAVSSCFARDQPSGWCLIGKNELEDTDAACCTEFPEISDASSLDLDGLSTTLTSALLLPPFTVCCRLN
TPALRRSGAASASPARSPGPRIANPSPEAQRCRFPPRDAGTRSNWFEEEERNWDKNCERNLIRSVRCWKH
YQMRRKGRCHRVSTTRHSSSPCSIKHSPTRETLTYAQAQRMVEIEIEGRLHRISIFDPLEIILEDDLTAQ
EMSECNSNKENSERPPVCLRSKRHKNNKVKKKNEALPSSHGVPATTAPLPEPKVRIVEYSPPSAPRRPPV
YYKFIEKSAEELDNEVEYDMDEEDYAWLEIINEKRKSDGVSVVSQNMFEFLMDRFEKESYCENQKQGDHQ
SLIDEDAVCCICMDGECQNSNVILFCDMCNLAVHQECYGVPYIPEGQWLCRHCLQSRSRPVDCVLCPNKG
GAFKKTDDDRWGHVVCALWIPEVGFANTVFIEPIDGVRNIPPARWKLTCYLCKQKGVGACIQCHKANCYT
AFHVTCAQKAGLYMKMEPVKELTGSGTTFSVKKTAYCDVHTPPGCIRRPLNIYGEAEIKNGVCRKEGSVR
TARSTSKVRKKTKKAKKTVVEPCTVMPTVSAPYIPPQRLNKIMNQVAIQRKKQFVERVHSYWLLKRLSRN
GVPLLRRLQSSLQSQRNTQQREDDEEMQALKEKLKYWQRLRHDLERARLLIELIRKREKLKREQVKIEQV
AMELQLTPFTVLLRSVLDQLQEKDSARIFAQPVNLKEVPDYLDHIKHPMDFSTMRKRLDAQGYKNLSEFE
EDFNLIIDNCMKYNAKDTIFYRAAVRLRDQGGVVLRQARRDAEGIGYDNETGMHLPERPKLESPQPFSWE
DVDRLLNPANRVHMSLEEQLRELLEKLDLTCAMKSSGSRSKRAKLLKKEISSIRNKLSQQHNQAPQIESG
IGSFEEESAALDQDGEEEGDKSPPKLEPSDALPLPSNLETNSEPPTLKPVELNPEQSKLYKRVKFDNELY
STCIQKEINGHTPEHLLDSNLNESTVSAVAESSTDVNRRTSILFCKSKSISPQKSAKNTETQPTSPQLGT
KTFLSVVLPRLETLLHPRKRSRSACGDSEVDDESPVKRLDTGLSNGFGDGSKERALDDAAGRKVEPRRRC
ASESSISSSNSLLCNTSFSLPKCGKGKPALIRRHTLEDRSELISCIENGNYAKAARIAAEVGHSSMWIST
DAATSVLEPLKVVWAKCSGYPSYPALIIDPKMPRVAGHHNGVTIPVPPLDVLKIGEQMQTKSDEKLFLVL
FFDNKRSWQWLPKSKMVPLGIDETIDKLKMMEGRNSSIRKAVRIAFDRAMNHLSRVHGEPVSDFSDID

Canonical Epc-N
PHD 6.3e-10
Bromodomain 2.1e-34
PWWP 5.8e-20

>XlaevisAAH73421
MRRKGRWHRVPAPRSSTSPCSIKHSPTRETLTYAQAQRMVEIEIEGRLHRISIFDPLEIVSEDDLTAQEI
SECNSNKENSEQPPQICLRSKRHKASKEKKKSEVLPAAHGHTTTSVLPEPKVRVIECSPPSAPHRSPSYY
KFQDKSSEELDKEVEYDMDEEDYAWLEIINEKRKSEGCSAVSQDIFEFLMDRFEKESYCENQKQGDQQSL
IDEDAVCCICMDGECQNSNVILFCDMCNLAVHQECYGVPYIPEGQWLCRHCLQSRNIPIDCVLCPNKGGA
FKKTDDDRWGHVVCALWIPEVGFANTAFIEPIDGVRNIPPARWKLTCYLCKQKGVGACIQCHKANCYTAF
HVTCAQKAGLYMKMEPVKEVTGSTTTFSVKKTAYCDAHMPPGCVRRPLNIYEEPESKNGICRKSSEKNIR
STSKARRKAKKAKQDLNETCNVLPTVTVPDIPPQRVNKIVNQVSIPRKKQFIERVHSYWMLKRLSRNGIP
LLRRLQSSLQSQRNVQESDDDEEVQALKKKLKYLQRLRHDLERARLLIELIRKREKLKREQVKVEQVAME
LKLTPLTVLLRSLLEQLQEKDPARIFAHPVNLSEVPDYLDHIKHPMDFSTMKKRLEDQRYRNLNEFEEDF
NLIIENCMKYNAKDTIFYRAAVRLRDHGGVLLRQARREAYVIGFDEETGMHLPEQPKIEPPPQFSWEDVD
KLLNPNNRAHMALEEQLKELLEKLDLTFAMKSSGSRSKRLKLLKREIANVRQMLSQQHVQPPQIESGIGS
FEEDGSILNDGEEEVLPRLETLLQPRKRSRSTCGDSDVDGESPVKRVDTGLSNGYGEGDKEKEFTITPGR
KLEPRRRCASESSISSNNSILCNSSFGLPKCGKGKPALIRRHTIEDRSELISCIENGNYARAARIAAEVG
ETNMWVAASTAAFLEPLKVVWAKCSGYPSYPALIIDPKMPRVGCHHNGVAIPVPPMDVLKTGDQMQTRAE
EKLFLVLFFDNKRSWQWLPKSKMVPLGVDETIDKLKMMEGRNSSIRKAVRVAYDRALNHLNRVQGESVSD
FSDID

Canonical Epc-N
PHD 6.3e-10

Bromodomain 3.8e-35
PWWP 1.4e-21

>MmusculusNP_084454
MGVDFDVKTFCHNLRATKPPYECPVETCRKVYKSYSGIEYHLYHYDHDSPPPPQQTPLRKHKKKGRQSRP
ANKQSPSPSEVSQSPGREVMSYAQAQRMVEVDLHGRVHRISIFDNLDVVSEDEEAPEEAPENGSNKENTE
TPAATPKSGKHKNKEKRKDSNHHHHSAPASAAPKLPEVVYRELEQDTPDAPPRPTSYYRYIEKSAEELDE
EVEYDMDEEDYIWLDIMNERRKTEGVSPIPQEIFEYLMDRLEKESYFESHNKGDPNALVDEDAVCCICND
GECQNSNVILFCDMCNLAVHQECYGVPYIPEGQWLCRRCLQSPSRAVDCALCPNKGGAFKQTDDGRWAHV
VCALWIPEVCFANTVFLEPIDSIEHIPPARWKLTCYICKQRGSGACIQCHKANCYTAFHVTCAQQAGLYM
KMEPVRETGANGTSFSVRKTAYCDIHTPPGSARRLPALSHSEGEEEDEEEDEGKSWSSEKVKKAKAKSR
IKMKKARKILAEKRAAAPVVSVPCIPPHRLSKITNRLTIQRKSQFMQRLHSYWTLKRQSRNGVPLLRRLQ
THLQSQRNCEQVGRDSDDKNWALKEQLKSWQRLRHDLERARLLVELIRKREKLKRETIKIQQIAMEMQLT
PFLILLRKTLEQLQEKDTGNIFSEPVPLSEVPDYLDHIKKPMDFFTMKQNLEAYRYLNFDDFEEDFNLIV
SNCLKYNAKDTIFYRAAVRLREQGGAVLRQARRQAEKMGIDFETGMHIPHNLAGDEVSHHTEDVEEERLV
LLENQKHLPVEEQLKLLLERLDEVNASKQSVGRSRRAKMIKKEMTALRRKLAHQRETGRDGPERHGPSGR
GNLTPHPAACDKDGQTDSAAEESSSQETSKGLGPNMSSTPAHEVGRRTSVLFSKKNPKTAGPPKRPGRPP
KNRESQMTPSHGGSPVGPPQLPIMGSLRQRKRGRSPRPSSSSDSDSDKSTEDPPMDLPANGFSSGNQPVK
KSFLVYRNDCNLPRSSSDSESSSSSSSSAASDRTSTTPSKQGRGKPSFSRGTFPEDSSEDTSGTENEAYS
VGTGRGVGHSSKYPHPKSGVLGTQFQGLASPPAADPPPLSRSCEVVRKSLGRGAGWLSEDEDSPLDALDL
VWAKCRGYPSYPALIIDPKMPREGMFHHGVPIPVPPLEVLKLGEQMTQEAREHLYLVLFFDNKRTWQWLP
RTKLVPLGVNQDLDKEKMLEGRKSNIRKSVQIAYHRALQHRSKVQGEQSSETSDSD

Canonical Epc-N
PHD 5.4e-12
Bromodomain 3.9e-35
AT hook
PWWP 4.6e-40

>RnorvegicusXP_235552
MRRKGRCHRGSAARHPSSPCSIKHSPTRETLTYAQAQRMVEIEIEGRLHRISIFDPLEIILEDDLTAQEM
SECNSNKENSERPPVCLRTKRHKNNRVKKKNEVLPSTHGTPASASALPEPKVRIVEYSPPSAPRRPPVYY
KFIEKSAEELDNEVEYDMDEEDYAWLEIINEKRKGDCVSAVSQNMFEFLMDRFEKESYCENQKQGEHQSL
IDEDAVCCICMDGECQNSNVILFCDMCNLAVHQECYGVPYIPEGQWLCRHCLQSRARPADCVLCPNKGGA
FKKTDDDRWGHVVCALWIPEVGFANTVFIEPIDGVRNIPPARWKLTCYLCKQKGVGACIQCHKANCYTAF
HVTCAQKAGLYMKMEPVKELTGGSTTFSVRKTAYCDVHTPPGCTRRPLNIYGDVEMKNGVCRKESSVKTV
RSTSKVRKKAKKAKKALAEPCAVLPTVCAPYIPPQRLNRIANQVAIQRKKQFVERAHSYWLLKRLSRNGA
PLLRRLQSSLQSQRNTQQRENDEEMKAAKEKLKYWQRLRHDLERARLLIELLRKREKLKREQVKVEQMAM
ELRLTPLTVLLRSVLEQLQEKDPAKIFAQPVSLKEVPDYLDHIKHPMDFATMRKRLEAQGYKNLHAFEED
FNLIVDNCMKYNAKDTVFYRAAVRLRDQGGVVLRQARREVDSIGLEEASGMHLPERPIAAPRRPFSWEEV
DRLLDPANRAHMSLEEQLRELLDKLDLTCSMKSSGSRSKRAKLLKKEIALLRNKLSQQHSQTPSIGAGTG
GFEDDAAPLAPDTGEEVLPRLETLLQPRKRSRSTCGDSEVEEESPGKRLDTGLTNGFGGTRSEQEPGSGP
GRKAAPRRRCASESSICSSNSPLCDSSFSTPKCGRGKPALVRRHTLEDRSELISCIENGNYAKAARIAAE
VGQNSMWISTDAAASVLEPLKVVWAKCSGYPSYPALIIDPKMPRVPGHHNGVTIPAPPLDVLKIGEHMQT
KSEEKLFLVLFFDNKRSWQWLPKSKMVPLGVDETIDKLKMMEGRNSSIRKAVRIAFDRAMNHLSRVHGEP
ASDLSDID

Canonical Epc-N
PHD 6.3e-10
Bromodomain 2.2e-32
PWWP 1e-19

>HsapiensAAF19605
MGVDFDVKTFCHNLRATKPPYECPVETCRKVYKSYSGIEYHLYHYDHDNPPPPQQTPLRKHKKKGRQSRP
ANKQSPSPSEVSQSPGREVMSYAQAQRMVEVDLHGRVHRISIFDNLDVVSEDEEAPEEAPENGSNKENTE
TPAATPKSGKHKNKEKRKDSNHHHHHNVSASTTPKLPEVVYRELEQDTPDAPPRPTSYYRYIEKSAEELD
EEVEYDMDEEDYIWLDIMNERRKTEGVSPIPQEIFEYLMDRLEKESYFESHNKGDPNALVDEDAVCCICN
DGECQNSNVILFCDMCNLAVHQECYGVPYIPEGQWLCRRCLQSPSRAVDCALCPNKGGAFKQTDDGRWAH

VVCALWIPEVCFANTVFLEPIDSIEHIPPARWKLTCYICKQRGSGACIQCHKANCYTAFHVTCAQQAGLY
MKMEPVRETGANGTSFSVRKTAYCDIHTPPGSARRLPALSHSEGEEDEDEEEDEGKGWSSEKVKKAKAKS
RIKMKKARKILAEKRAAAPVVSVPCIPPHRLSKITNRLTIQRKSQFMQRLHSYWTLKRQSRNGVPLLRRL
QTHLQSQRNCDQVGRDSEDKNWALKEQLKSWQRLRHDLERARLLVELIRKREKLKRETIKVQQIAMEMQL
TPFLILLRKTLEQLQEKDTGNIFSEPVPLSEVPDYLDHIKKPMDFFTMKQNLEAYRYLNFDDFEEDFNLI
VSNCLKYNAKDTIFYRAAVRLREQGGAVLRQARRQAEKMGIDFETGMHIPHSLAGDEATHHTEDAAEEER
LVLLENQKHLPVEEQLKLLLERLDEVNASKQSVGRSRRAKMIKKEMTALRRKLAHQRETGRDGPERHGPS
SRGSLTPHPAACDKDGQTDSAAEESSSQETSKGLGPNMSSTPAHEVGRRTSVLFSKKNPKTAGPPKRPGR
PPKNRESQMTPSHGGSPVGPPQLPIMSSLRQRKRGRSPRPSSSSDSDSDKSTEDPPMDLPANGFSGGNQP
VKKSFLVYRNDCSLPRSSSDSESSSSSSSSAASDRTSTTPSKQGRGKPSFSRGTFPEDSSEDTSGTENEA
YSVGTGRGVGHSMVRKSLGRGAGWLSEDEDSPLDALDLVWAKCRGYPSYPALIIDPKMPREGMFHHGVPI
PVPPLEVLKLGEQMTQEAREHLYLVLFFDNKRTWQWLPRTKLVPLGVNQDLDKEKMLEGRKSNIRKSVQI
AYHRALQHRSKVQGEQSSETSDSD

Canonical Epc-N
PHD 5.4e-12
Bromodomain 3.9e-35
AT hook
PWWP 4.6e-40

>Rnorvegicus XP_342736
MGVDFDVKTFCHNLRATKPPYECPVETCRKVYKSYSGIEYHLYHYDHDSPPPPQQTPLRKHKKKGRQSRP
ANKQSPSPSEVSQSPGREVMSYAQAQRMVEVDLHGRVHRISIFDNLDVVSEDEEAPEEAPENGSNKENTE
TPAATPKSGKHKNKEKRKDSNHHHHSAPASAAPKLPEVVYRELDQDTPDAPPRPTSYYRYIEKSAEELDE
EVEYDMDEEDYIWLDIMNERRKTEGVSPIPQEIFEYLMDRLEKESYFESHNKGDPNALVDEDAVCCICND
GECQNSNVILFCDMCNLAVHQECYGVPYIPEGQWLCRRCLQSPSRAVDCALCPNKGGAFKQTDDGRWAHV
VCALWIPEVCFANTVFLEPIDSIEHIPPARWKLTCYICKQRGSGACIQCHKANCYTAFHVTCAQQAGLYM
KMEPVRETGANGTSFSVRKTAYCDIHTPPGSARRLPALSHSEGEEEEDEEEDEGKSWSSEKVKKAKAKSR
IKMKKARKILAEKRAAAPVVSVPCIPPHRLSKITNRLTIQRKSQFMQRLHSYWTLKRQSRNGVPLLRRLQ
THLQSQRNCDQVGRDSEDKNWALKEQLKSWQRLRHDLERARLLVELIRKREKLKRETIKIQQIAMEMQLT
PFLILLRKTLEQLQEKDTGNIFSEPVPLSEVTELDEVPDYLDHIKKPMDFFTMKQNLEAYRYLNFDDFEE
DFNLIVSNCLKYNAKDTIFYRAAVRLREQGGAVLRQARRQAEKMGIDFETGMHIPHNLAGDEVSHHTEDV
EEERLVLLENQKHLPVEEQLKLLLERLDEVNASKQSVGRSRRAKMIKKEMTALRRKLAHQRETGRDGPER
HGPSGRGNLTPHPAACDKDGQTDSAAEESSSQETSKGLGPNMSSTPAHEVGRRTSVLFSKKNPKTAGPPK
RPGRPPKNRESQMTPSHGGSPVGPPQLPIMGSLRQRKRGRSPRPSSSSDSDSDKSTEDPPMDLPANGFSS
GNQPVKKSFLVYRNDCNLPRSSSDSESSSSSSSSAASDRTSTTPSKQGRGKPSFSRGTFPEDSSEDTSGT
ENEAYSVGTGRGVGHSMVRKSLGRGAGWLSEDEDSPLDALDLVWAKCRGYPSYPALIIDPKMPREGMFHH
GVPIPVPPLEVLKLGEQMTQEAREHLYLVLFFDNKRTWQWLPRTKLVPLGVNQDLDKEKMLEGRKSNIRK
SVQIAYHRALQHRSKVQGEQSSETSDSD

Canonical Epc-N
PHD 5.4e-12
Bromodomain 9.3e-31
AT hook
PWWP 4.6e-40

>GgallusCAH65210
MGVDFDVKTFCHNLRATKPPYECPVGSCRKIYKSYSGIEYHLYHYDHDNPPPPQQPPLRKHKKKGRQGRA
GAADKQSPNPSEASQSPGREVMTYAQAQRMVEVDLHGRVHRISIFDNLDVVSEDEEAAEEEAPENGGSKEN
GESQAAAAAAAAPKSAKQKSKEKRKDSNHHHHHNASAGAAPKLPEVVYRELEQDTPDAPPRPTSYYRYIE
KSAEELDEEVEYDMDEEDYIWLDIMNERRKTEGVSPIPQEIFEYLMDRLEKESYFESHNKGDPNALVDED
AVCCICNDGECQNSNVILFCDMCNLAVHQECYGVPYIPEGQWLCRRCLQSPSRAVDCALCPNKGGAFKQT
DDGRWAHVVCALWIPEVCFANTVFLEPIDSIEHIPPARWKLTCYICKQRGSGACIQCHKANCYTAFHVTC
AQQAGLYMKMEPVRETGANGTSFSVRKTAYCDIHTPPGSVRRLPALSHSEGEEEDEEEEEEGKSWSSEKV
KKAKAKSRIKMKKARKILAEKRAAAPVVSVPCIPPHRLSKITNRLTIQRKSQFMQRLHSYWTLKRQSRNG
VPLLRRLQTHLQSQRNCDQRDTEDKNWALKEQLKSWQRLRHDLERARLLVELIRKREKLKRETIKVQQVA
LEMQLTPFLILLRKTLEHLQEKDTGNIFSEPVPLSEVPDYLDHIKKPMDFQTMKQNLEAYRYLNFDDFEE

DFNLIINNCLKYNAKDTIFYRAAVRLREQGGVVLRQARRQAEKMGIDFETGMHFPHCVTVEEAQVQDIED
EDMRLLLSENQKHLPLEEQLKILLERLDEVNAGKQSIGRSRRAKMIKKEITVLRRKLAHPRDLGRDGLER
HSSSARGVLQSHNPCEKDLQTDSAAEESSSQETGKGLGPNSSSTPAHEVGRRTSVLFSKKNPKTAGPPKR
PGRPPKNRDSQIAPGHGNSPIGPPQLPIMGSSQRQRKRGRSPRPSSSSDSDSDKSTEDAPMDLPANGFSS
GSQPVKKSFLVYRNDCNLPRSSSDSESSSSSSSSAASDRTSTTPSKQGRGKPSFSRVNFPEDSSEDTSGT
ENESYSVGAGRGVGHSMVRKGIGRGAGWLSEDEDSSLDALDLVWAKCRGYPSYPALIIDPKMPREGMFHH
GVPIPVPPLEVLKLGEQMTQEAREHLYLVLFFDNKRTWQWLPRTKLVPLGVNQDLDKEKMLEGRKSNIRK
SVQIAYHRAMQHRNKVQGEQSSDSSESD

Canonical Epc-N
PHD 5.4e-12
Bromodomain 7.7e-35
AT hook
PWWP 4.8e-40

>DrerioAAH44418
MGLDFDVKTFCHNLRATKPPYECPVESCRKVYKSYSGIEYHLYHYDHENPPPPPQGTPQKKRKGRPPRGS
SDGPDSSTGPGATSGQNTAPSSPAERSERSHSPVRETMTYAEAQRMVEMEIRGRVHRVSIFQNLDVVSEE
DSGAEEPPGATGSTAAACNGGGESGGGDRMDTANGEKGAQKTGKHKSKEKKKDSGAHHHINTSGPAVKLP
EVVFRELDQDRPDAPPRPMSYYRYIDKSAEELDEEVEYDIDEEDYIWLDIMNEKRRSDGVAPIPQEVFEY
LMDRLEKESYFESHNKGDPSALIDEDAVCCICNDGECQNSNVILFCDMCNLAVHQECYGVPYIPEGQWLC
RRCPQSPSRAVDCALCPNKGGAFKQTDDSRWAHVVCALWIPEVCFANTVFLEPIDSIEHIPPARWKLTCY
ICKQRGSGACIQCHKANCYTAFHVTCAQQAGLYMKMEPVRETGANGTSFSVRKTAYCDIHTPPGSARPLG
GVGGVSADSSRSEAEPEEEEEPPAAEDDGKGWSSERAKRAKAKSRLKMKRARKILAERRAAAPVLSVPCI
PPHKLSKITSNLTVQRKSQFMQRLHSYWTLKRQSRNGVPLLRRLQTHLQSQRNTEPLPVTRDGEEKHSVL
KEQLKAWQRLRHDLERARLLVELIRKREKLKRETIKVQEMAMEMQLTPLLVLLRRTLEQLQERDTNNFFT
EPVPLSEVPDYLDHIERPMDFHKMWKCVESHRYLSFEAFEGDFLQIVNNCLKYNAKDTVFYRAALRLREA
GGVVLRQARRQAERIGFDYETGLHLHREPSPENTHTHQRDRERERVYIEGELLSESWRRLPVDEQLQILQ
SRYEEAASGKHSIGQQRRAKALKKEMTALRRKLAHPRDTLLLDTHSHTHILSKDGVCVV

Canonical Epc-N
PHD 1.4e-10
Bromodomain 5.2e-24

>MmusculusBAC31528
MGVDFDVKTFCHNLRATKPPYECPVETCRKVYKSYSGIEYHLYHYDHDSPPPPQQTPLRKHKKKGRQSRP
ANKQSPSPSEVSQSPGREVMSYAQAQRMVEVDLHGRVHRISIFDNLDVVSEDEEAPEEAPENGSNKENTE
TPAATPKSGKHKNKEKRKDSNHHHHSAPASAAPKLPEVVYRELEQDTPDAPPRPTSYYRYIEKSAEELDE
EVEYDMDEEDYIWLDIMNERRKTEGVSPIPQEIFEYLMDRLEKESYFESHNKGDPNALVDEDAVCCICND
GECQNSNVILFCDMCNLAVHQECYGVPYIPEGQWLCRRCLQSPSRAVDCALCPNKGGAFKQTDDGRWAHV
VCALWIPEVCFANTVFLEPIDSIEHIPPARWKLTCYICKQRGSGACIQCHKANCYTAFHVTCAQQAGLYM
KMEPVRETGANGTSFSVRKTAYCDIHTPPGSARRLPALSHSEGEEEEDEEEDEGKSWSSEKVKKAKAKSR
IKMKKARKILAEKRAAAPVVSVPCIPPHRLSKITNRLTIQRKSQFMQRLHSYWTLKRQSRNGVPLLRRLQ
THLQSQRNCEQVGRDSDDKNWALKEQLKSWQRLRHDLERARLLVELIRKREKLKRETIKIQQIAMEMQLT
PFLILLRKTLEQLQEKDTGNIFSEPVPLSEVPDYLDHIKKPMDFFTMKQNLEAYRYLNFDDFEEDFNLIV
SNCLKYNAKDTIFYRAAVRLREQGGAVLRQARRQAEKMGIDFETGMHIPHNLAGDEVSHHTEDVEEERLV
LLENQKHLPVEEQLKLLLERLDEVNASKQSVGRSRR

Canonical Epc-N
PHD 5.4e-12
Bromodomain 3.9e-35

>TnigroviridisCAG11339
MGLDFDVKTFCHNLRATKPPYECPVETCRKVYKSYSGIEYHLYHYDHDNPTPAQTVPQKKRKGRPPRASL
AGSGDTDDGGNGGGGPGHGGNTPGSPNRSEHSHSPGRETMTYAQAQRMVEVEIQGRVHRISIFDNIDVVS
EEDSDAEDLPPSGTGGTGGGVCNGSDGGGSELGGSGKDRPDIPSSNGGKSTPKTSKHKSKEKKKDGSYHH
GAQSGPAVKLPEAVFRELDQERPDAPPRPSSYYRYIDKSVEELDEEVEYDIDEEDYIWLKIMNDKRKRDG
VTQIPQEVFEYLMDRLEKESYFESHNKADPSTLIDEDAVCCICNDGECQNSNVILFCDMCNLAVHQECYG

VPYIPEGQWLCRRCLQSPSRAVDCALCPNKGGAFKQTDDARWAHVVCALWIPEVCFANTVFLEPIDSIEH
IPPARWKLTCYICKQRGSGACIQCHKANCYTAFHVTCAQQAGLYMKMEPVRETGANGTSFSVRKTAYCDI
HTPPGSARPLGGVGGASIGSSHSEGELEDDDEGSIGHDDDSKGWSSERAKRAKAKSRLKMKRARKILAER
RAAAPVVSVPCIPPHRCVDAETYPNPQSDLSVVRMCVFTCLLFLSRLSKITSNLTVPRKSQFMQRLHSYW
TLKRQSRNGVPLLRRLQTHLQSQRHIDPLPPQPPDGEEKQSALKEQLKAWQRLRHDLERARLLVELIRKR
EKLKRETIKVQQMALEMQLTPFLVLLRSTLEQLQERDTNNFFTEPVPLAEVPDYLDHIDTPMDFQTMWNQ
LESHRYLTFEAFEADFGLIVNNCLKYNAKDTVFYRAALRLREMGGSVIRTARRQAERIGFDYEAGLHLPR
ELSPETRRDQERDREHLLLPENRRRLPLEEQLHYLQARLDEVSSGKHSIGQSRRAKALRKEISVIKRKLA
HQREGVSNMGSRDSVGGASCLPHHASSVGHLDEGGESSSQEIGGKELSVSSSALAPEVGRRTSVLFSKKN
QKMAGPPKRPGRPPKNRDAGYSGGGISQSPIGPPQLPLLATARQRKRPRSPHSSSTSDSDSDNEDLLPGL
PSNGFDGGNQPVTESFRVYRNEPRLPRSSSDSESTTSSSSSAASDRTSTTPSKQGRGKASFSRSAFQEDS
SEETSGTENDSYSVGGSRGVSHLVRGRARSGCWMSSDDYSSLEALDLVWAKCRGYPSYPALIIDPKMPRE
GVFHRGVPIPVPPLDVLKLGEQMTQEAREHLFLVLFFDNKRTWQWLPRSKLVPLGVDQELDKEKMLEGRK
SNIRKSVQVAYHRAMQHRSKVQGDPSSETSDSD

AT hook
Canonical Epc-N
PHD 5.4e-12
Bromodomain 1.3e-26
AT hook
PWWP 4.8e-40

>CbriggsaeCAE60017
MGRGRGSGGGGASSSSGGIPLSEYQNIDPLDEESLNDRTQTDQGLFDGKVVMLNYKSKASKTESMEARAK
KWAAKSPEEKVSKFNKIFHPQSTKKWPPTMKEPPVESVAFFVHSTKGKFGMPLKKFPEQKYDHQRIVVKK
AENPVDYSIDEHDITWLMRMNAHQASQGRTMLTVATFEHWVDRLEKNSVWKPKEHHKLRDEHGEELEDVC
NICLDPDTSNCNQIVYCDRCNLTVHQDCYGIPFIPEGCLECRRCVVSPAKRVSCVLCPSRTGAFKQVDHK
RWVHVLSNFPLFQPPKIPPYLQCSESKCTASFHVTCARNHGLIMRIVELDNGFVKRFVWCPRHRPVETEA
DKEQHERMLRNQKRENERMLPQISMPTLTNGIISQIQSERPFAYFREIVLFWYQKRQNRLGAPLLKTWTP
PESLIIDLFKTPEGRRRGSSGVRATTTTTILLDSEELEKKKEEFEMGIQKAMEMFAAVMDEEKDKIEMSK
SSLKMLELGFKTNEMYCAETLENIKRKDTVKVFEKPVQLPIYTTIIKNPISFEEMTKKAAEFKYNSITQL
QADIALMLTNCSIFNKKNPYYYKYGITFKRQTTPMLEAAARAEAARSERRNDKELMTEILNGFLNANSEA
GGAEDVEEDSGAPGGSGGSGGAGKRGRRRRSPGKVKDVKEEIKMEEEPPASALPSKRGRKRAIQETLDSE
EAPSTSSEPSGAPDMLNAASTFFKPPQTRLFRGAGFPGIHKKFGASICETPKTSGPQKQTKMTAFLTRGV
TFEDEKGGGDKDGDPKQKTKNKKDNNEKSDCQQQHILFQSITTPSNSTKLFPNSFTSILASFTGIPTIPR
PVTRSSTTSTNSGDSAATSSETPKIQKSKFRISSSALQSPLPDPKRVGIRAMDSEDDDDEEEILIQPIQK
TLSPEELKTEQLRCAENESKSKFAHNQLVIVEGRAAKVIDYHLAHLSDIHLEQRQSMLKKRREVLAELPS
SAPIYVEFFQKSNILENFQWVPSEKVELLDLNNCAQKGKITGLKAAKECIFHFFFSKNDRFVFPENSIIH
FLVPVFLTKKNQ

Canonical Epc-N
PHD 1.6e-06
Bromodomain 9.6e-14

>CelegansAAB03164
MGRGRGVAIQETIEECRDTISDRIQYNLGLNESKVVLMDIVTGPNQNVSLQERTKRWMAVSVDARMTKFK
KNFYPTNQKNSKNISKDLDPPIQRVDAHIVTPVAGKCGMPLQKFPEFKHDHEKIKIERDAKYVDYSVDEF
DMSWMSIMNAKRTKLGLEIFSVAIYEHWVDRLEKMCIWKPKEFHKLKDENGEELDDVCNICLDGDTSNCN
QIVYCDRCNLSVHQDCYGIPFIPEGCLECRRCGISPAGRVNCVLCPSTTGAFKQVDQKRWVHVLCVIWVD
ETHFGNTIFMENVQNVEKALHDRRALSCLLCKNRQNARMGACIQCSETKCTASFHVTCARDSGLVMRINE
TEDGQVNRFVWCPKHAPPLTDADREMRQLMLRNARRENERKGPMISMPTLMKSMISTICVERPFSDYSEI
IYFWYEKRLNRLGAPLLKNFTQGASKSRRLLPKSTICGQLKNVETCEMKKQVNAVKESLASGLEIFDMIV
RREERKKDMLNSYIRMFERGFKPTELLCQEVIEALKTIDAGKVFAEPVELVGYTDIIENPICLKDMSEKA
ASGKYSTVAALSADVQLMLSNCATFNKGNRVYIKYGNTYRKDSTPILEIAEKEEVERLALKTDEKFMTQL
LNGVMVEYNGWAQSRNEVAKEIPPPTPSRGRGTGRRRQNPFLDVQELDTDDSKDSSALSEIPGSSKKSSR
KRGIQDTKMEEDEEIKPSTSGTNAVASVPLLSSSRESKNKSKSSDADVSSPKSSWLGSPSTSQNLRRRVQ
GFSGNESSPKVHKKLSTDNPSNSNLRQTTLTNFFGTNPKTQQQVTFADMTATPSGSGNKNVSQRSLFDTP
STSKASSFTSLSSTRPSTRSTSIIPTINKKNAFRMSSASIQSPLPTTKKIGVRAMATDDEEEDIVIQPPP

KEMTTQELEAEKLKSAENEAMSKFAHNQLVIVDGRAAKVIESRLAHLASDIHHEQRQSMMKKRREVLSEI
PQAAVIYVEFFQKSNTLENFQWVTPDKVELLDLNNIGQRSPKIPGLKAAKEWHQKVLNGEDV

Canonical Epc-N
PHD 1.4e-06
Bromodomain 4.6e-20

>OdioicaAAS21398
MEDNIKEEADFTLAKQYKGRDLNPDIDVQSYINKIDAESAVYPYVCPKCGRPYKDEVMLEVHLDSFDHNN
PPDDYDPCDLHWKPKKRVERLKEEFERESRDSGSDTNGPSTPKNTRTPGTRGFGSHKRKRNTWSRKRKAN
FRRGSDVSDTPRTKTRVRVDEHGRITTENLTWSEAQRLVEFEDDNTNKIYRVSIYEDVEVVKSDGSELPA
LHKGARVPVPDTVVTEMEVPKLQIRKISSEYIDYKAQPTENTIEYEMDEEDHVMLEHINDVRESDGLKPL
SCETLEKFIDAIEKESMWDTGNKPRTSTRDIEDEDVVCCVCNDGECTNTNAILFCDLCNLAVHQECYGVP
YIPEGQWLCRRCQFSPSRPVDCVLCPSLNGAFKQTHDNRWCHVVCALWVPEVSFQNPVFLEPIDGFSKVP
KARWNLKCYICKKAGACIQCNKNACYTAFHVTCAQQAGLYMEMKVQKSDGINGITSNKVTQTAFCHNHTP
KDWSPRPLKSSEKLPKAANRADQFQAKVDETREAVEGRNSSRSVNIPYIPSDKLGVLRAILSDNVTSKVS
PLFKQLLTFWNEKSMVCNGVHLEQLRVWQRLRHDLERARLLVEQIKKRERLKAHEIQKQIQLMRLRINPW
SYFLRDIIDKLKELDESQIFYHPVTDAIAPLYSKIITSPMAFATMERKVTNSEYKCFAAFEYDVNLILRN
CMHYNGNSTIFYKLAKDMETKAQPMLRDARKLAKKYNTVTGQHLQPGDKFDQKEAEVEHIRGVLGDLRKC
LRKAQKSPSSKHQKKTVQKLTADIKIFERRLRTMGVEEFETSSPKKAKLEENKNEEEDDEFKNEPDTETE
EPQEETVEDGQAVDDETKDEVESADDDVKEEPDESPPVENGETTPLTEVHENGQELKDPEEPTLKKELAN
GTVKEPAENELSQFIEEDSNDSQEEIKSSNKKIDLRNFQPLDTGYPWYPARIINPELTTGEPLLLGDEEI
PQPSVDIIKAGNRLNLEGDSQHHLVLFFDQKRTWMWLPSNKIALLGKDNKFDNTKLREGKTNAQKKSISR
AFKCALEPLSPD

C2H2 0.00089
Canonical Epc-N
PHD 1e-13
Bromodomain 7.8e-20
PWWP 8.9e-06

>UmaydisEAK86867
MPGIPSKPNNIPLASSLPKVSFRKVPPEEALLFRIPPGFIDAQALPFGYNDGSEYDKPDHYIRYVEPIEG
DLKKQVEYDMDEQDQEWLDAFNYERRKEGLDSISYEIFEIILDQLEKEWFDLMKRIPPKARHSAATEVAA
EGADADDADSEGGDDSKCAICDDGECENSNAIVFCDGCNLAVHQDCYGIPYIPEGQWLCRKCTVSPDRAV
SCILCPHEGGAFKQTTTGKWAHLLCAMWIPETGVSNPVYMEPIDSVERIPKARWKLQCYLCRYRMGACIQ
CDNRSCFTAFHVTCARKAGLLFRTERTRVSHHLYDDSDNSDDEGSEVLRACCHRHMPADLRHQFKIDFGR
QAALEDDRSEASYRASPFVSSRTRELSVESGKGAPLISVSRRSSLVGSNESATATSDPRSTSKSARAYKK
SFKAGPPLVPAYIANRVLEYITKVQLRKKATVVQLIARYWSLKREARRGAPLLKRLHLEPWTASSQNKEQ
TDAQKAKKLHFLRSIREDLERVRMLVEQVRKREKEKLRQAQEIRNSLIEPVLFPFHTDLRAAIAKLEAVD
RYGFFAQPVSKVDVPDYYDIVKDPMDWATIKDKIAHKAYDTVEEIRQDVLMIATNAMTYNKADTPYHKAA
SKILKMIPEVFKELAAIETSHLVYHQQKRVQAEPTTDTDSKEGSRKHAFVTSDTSNKQIYTIDAESRRQL
IELGLEPPSDLVSLLRDYCLMEEEEQLQIRQQAYNTHPMPPTTHVKAEEHGHADNQADPTASQRRRLTAP
VMNLMEDFVQQLYVPPPRPAPVERDSLPGSANGKRSARMSAAVPAEKTSVRAARKRNPSDSQPKEPVERR
STRRSIAAETEAEVKSEQAIAEALPATPVRSRRRSTQSSAHAASTEHIDAQSDARPQSSSMQTSLSQTSA
KTQDGVQVKADVGAHDSFLLFNTGWVLPEGSKRHRSSAARPEMIGQRPRKISAPESPVAFGASRAAKSSS
PTTPQQRPRSRTAPSTDPRSVPASSTAKAKDSSSTRKEAGSKPDRRNSKDTDRAASSPLTTDDEGEKHAG
AQSARKPNRLKRNRGEELSAASQAGSEAASNETASTTRRSARQATSLIDPSASPPAAKRRRAECKVSRSK
EAFDYVTHPPAAGTKVWAKVETFPHFPAIVIRDKTDLPGEMLRTQPEDESMVAVRFFGKQTSYGWITPLK
LAPLLMDESEDEKYLKLAAKKGKTKQVRDAYEDAKSY

Canonical Epc-N
PHD 2.5e-15
Bromodomain 5.7e-23
PWWP 7.1e-08

>DdiscoideumEAL65544
MVMMEGVTPPPTAKVIPFSLKKKKPAALVPTTATVATTTTTTTTNSTTNQNSNDNTPVVIETNNNNNNNN

NNNNNINNTTETIATTTETTETTATIENDSPTISSEKIKTSTMEPINPASIISNNNNNNNNNNNNNNNNN
NNNNNNNDNDNNNDTIMKNIEDEEEDLYTDITFVSGPSTNVEAEDEQEEVEKQPQQTQPQQQQQQRLDIN
KKTDTNINGVIQMDTDSKSLSSSSSSSSSRYQKTPKKNNNNNNNNNNSINNKANNEAFFDKDSFKIKLQVI
NPESYSEEEIQDINLNSVTSEVYMNPTTLGDKLQQHQQQQHQKQQQKQKNKNTTTSNNNNKNKNNNKNHH
DHAGSKDQINSLEDEKRYTQRYRYLKSNEEILNKPVFKEIEESPMAPYNKPSGYIIYQEKSSEMLHDEVE
YDMDSEDEKWLEEFNKTTNNNYSEDIFEYVIDRLEKETFKYKQEIGSDMFPHFSSVEADDVCGVCFDGTS
DDTNQIVYCDGCDIAVHQDCYGILLIPEGHWFCQKCESPDKDSISCVLCFKKNGSFKQTIDGEWVHLVCA
YNIPELSQIIKKGSGREKLGPPGLLSNILKKRKKLKCIICKKIGGACIQCRERHCSVAFHPYCIKMNQKS
MIDENLNPHIIFCKKHFLRNNNINGNVKDILDDDGSSSDGSEKENSSDDDDYTDSSDEHDISGDDDNDDD
DNDDDSFEVIKTPSKKSHISNKSSKSTPSKSNTNLKSKPQPPKPKLKSKSKSTPSKKKANHKKKKKKIDS
TDEDEDSNNEKEDENEEDEDEEDSEEEEEEEEEEEKEEKEKINNKNKKRNNKNNKNNNIIIDGRKKKKLKT
TDSNNNKVATVTAKDTTTTTTTTTTTTTTTTSTTTTGITAKKVGLISSSRAAKVIRKVPSTFVHAVYDYWV
KKRISRSGLALIKRFQPSILFSLRSTLHAKDPGINSVSDYNKMLSIREDLERTRTILELVKKREKQKREL
IINIRNEIESTILNDYYFLRMVLKSLESIEQLRPFYVPVLEEEAPSYFLIIKQPMSFQQIKQSLNTYAYE
NTDDFWKDITLIYTNAQLFNCSKTSVYKASRLLQEIANKLESDCPPKVTPEELDDFIKNIKYPSKKLLTT
NHKKFSTPLKSVSSSVSSSSSSSSNTTNTQSTPIKTTPTETPKPTSRTRSMVAQEQEQEQGKGKEKEQEKE
KEQGKGKGKEKEKEKEKEKEKEKEKEKEKEKEKEKEKEKEKEKEKEKEKEKENEEQVIPKKRRGCPSQPAPRTRQS
DSKLASIEPTEDQRLSKTPPTSKATSSKTTQPITPKTPQPTTPKTPQPATPKTPQPPRTTPTSSSKTPNI
TSPMSASKIITRSSTPSKTTLQKKEIPPQKKITAKQTPQRIIKPTLKSQQVIPATTSTISRTRRNLQKQL
EETLENDQDQKELVEENENENENENENENENENENENENENENENENEEEEIEEQEMKQKEEKEEEEEDDEG
EEQKGIIQKGDEIDTDVEDYGVKRKKKSLSPLKLTIPNTNQLMLLDTMNTQTEKKSRSKSILDQTTKDLF
VVPQIIKRVAIMSQLKHIPLEINFSKEITDDLRELLSITQEIQSIEKGGFLSHANRAVSSKEGLKFIEHI
DLRIFRGTYDHDKKLFWYHLEKIAQRIPPQFSKSKKLTLEKISELKEKYEQNHPEILDEENNEEENDE

Canonical Epc-N
PHD 4.7e-14
PHD 3.1e-05
Bromodomain 5.3e-18

>TnigroviridisCAF96088
MKKRARQHRPSVPRRPSSPIKPSPNKQILTYAQAQRMVEFDVDGRVHRISIFDKLDVIWDDDPTAQEMME
CTSNKENAEKPQQQQQILVRSVRLKNNQEKRNAALGLTGHGDGGENHASVHSGPKLPEPKFRTVEYNLPA
VPRRPTTTYYKYAEMTEEELDEETEYDMDEEDYAWLELVNEKRRSEGVSQVSFNVFEFLMDRFEKELYLEN
LDQGSEMHAAIDEDAVCCICMDGECHNSNAILFCDMCNVAVHQECYGVPYIPEGQWLCRHCLQASSQPAE
CVLCPNKGGAVKKTEDERWGHVVCALWVPEVGFSNTTFIEPIDGVGQIPPARWKLTCYLCKEKGVGACIQ
CHKANCYTAFHVSCAQKAGLFMKMEPVTEVKESGEATFSVRKTAYCGAHTPNGCVRRPLTIYEDSQPKNG
LCKKADRSRSLAKGHSKGKQRKKSKNKKPETESESEEPTPAPAPSFPAHRLQNILNQVSIQKKKAFVELV
LNYWKLKRQSRNGHPLIRRLQTSLHAQKNTQLGQNEEESRALKEQLKEWHRLRHDLERARLLLELIRKRE
KLKREEIKQHETLMEMQLTPFSILLRALLDQLQMKDQARIFAQPVDVSEVPDYLDHIKDPMDFSTMRQRI
DAQSYSNLDQFEKDFNLIIDNCMKYNSKDTYFYRAAVRLRDQGGALLRKARRDIEKIGFDSESGMHLPDA
PKIQDSPPFSWEDVDRLLLPANRQHLPPDKQLQQLLEKLDLTCSMKSSPSRSKRLKLLKKTINDVRSEMS
LSRVVPSAHHPPSSTPTSTPAHPEPSAAADKEKSKSNGHFTDDEGDKSLPPKLELSDSIPPLIHSDADPE
PPTLKPIDAAPDRGGKARRKSADSDGEIPQLLPSVPHLNGYAHDGAASSLLEGDATTAEAAGAVNRRTAV
LFRKSKAASPHKPPRDGDRATDVGAEAGKEGQDADEAQLGLQILPVGGLSSGFLGGEEEKELSESPSRAG
QAVEPRRRCASESSISSSSSLQGSTSTTLSLPKCGKGKPALVRRNTVEDKSELIACIETGNFAKAARIAA
EVGNSNIWMPASAATVALEPLKLVWAKCSGYPSYPALIIDPHMPRAGCQHNGVSIPTPPTDVLRIGEQMQ
YKAEEKLYLVLFFDNKRSWWLPRSKMVPLGLDKTIDKIKMMEGRTSSIRKAVQVAFGRAMNHLSIVQDEP
VSDLSDVD

Canonical Epc-N
PHD 3.1e-10
Bromodomain 1.3e-34
PWWP 1.3e-19

>CneoformansEAL20164
MPLHDLPKVSFTLITDDPALLHPAGVQSEQSRSYGYNDFSDFERPEHYIRYIEPIESELAVQVEYDMDEQ
DQAWLDTYNAERKKDQCGPISYEVFEIIMDKLEKEWFNLSKRIPQPQQQLAPEDSKCSICDDGEGENSNA

IVFCDGCNLAVHQDCYGVPYIPEGQWLCRKCTVSPENPVSCIFCPNEGGAFKQTTTGHWAHLLCAIWIPE
TTLGNSIYMEPVESVELVPKGRWKLVCSLCKERVGACIQCENRNCFTAFHVTCARQLGLLKSMKSLTTDG
TLHAYCHKHMPLDESKDQEVEDEEEEDDGWEVSPRNHHAHAKTKSKTQSSKSRTSKSHPTPTAKRPAQPL
VIPVTKKSAQAHSKSFRPGPPIIPKMIVNRILEYYVGKVQLRKKPQVVEKICRYWSLKREARRGAPLLKRL
HLEPWTASTAARTQTESEKAQKLKFLQMLRNDLEKVRMLADLVRKREKEKLRQAQVIKDVIDRFIFPYSE
RLRVTLERISAMDRREMFLNPVTPAEAPDYSDIVKEPMCWLYIDEKLEKNAYIDIADFKRDIMLVLDNAM
LYNARDTPFHRAASKLKTAAQPLLNELDSITASRQSTSTESLVPSDLPVGDLEPALPHILAFFQETTVSS
QPEEPQDLLTSLFATELEPPKPPTPTPSPPPQPRKYRRPTKEEKQKKWNALVKERQEKGLGRSTRGTEKL
TREFEEEAGLAAAATQREKSTPVPAEEGNGMGIDHDGRAVRRSTRNSLGGASSAVSTPKSAPRQRKREPA
QVPVAEEPSRSARSTSTILPPTRQQRGVIGLEALPLLTTKERLEQERALDLTTTEIDAADQFKRFNVGWV
LPEEEEEGEIEVTTPKRSDKGNAVEPENGGFIEAADDDSDLSPPPTSLPGSAAPSPRATSSVAKPADAHG
SDIENDARSDDEDQLQTEKKKPDLEGQGDQETISHGTKRKGESDIETPVREKKRSKTTQAKEEKVAKAPP
KGKGRVKALYPPGTLVWAKIHTFPFFPAEIVDLIDDRDEVPDAVLKEETASRAAAKNVNKDVWLVRFFDA
RSSYGWITGDRLDELGEDENVDAMYLAGKDREGKSKGFKTPSLKRACRKGYRDALNSMETSANEGGRKEK

Canonical Epc-N
PHD 7.1e-16
Bromodomain 8.9e-18
PWWP 2e-05

>HsapiensNP_056510
MRKPRRKSRQNAEGRRSPSPYSLKCSPTRETLTYAQAQRIVEVDIDGRLHRISIYDPLKIITEDELTAQD
ITECNSNKENSEQPQFPGKSKKPSSKGKKKESCSKHASGTSFHLPQPSFRMVDSGIQPEAPPLPAAYYRY
IEKPPEDLDAEVEYDMDEEDLAWLDMVNEKRRVDGHSLVSADTFELLVDRLEKESYLESRSSGAQQSLID
EDAFCCVCLDDECHNSNVILFCDICNLAVHQECYGVPYIPEGQWLCRCCLQSPSRPVDCILCPNKGGAFK
QTSDGHWAHVVCAIWIPEVCFANTVFLEPIEGIDNIPPARWKLTCYICKQKGLGAAIQCHKVNCYTAFHV
TCAQRAGLFMKIEPMRETSLNGTIFTVRKTAYCEAHSPPGAATARRKGDSPRSISETGDEEGLKEGDGEE
EEEEVEEEEQEAQGGVSGSLKGVPKKSKMSLKQKIKKEPEEAGQDTPSTLPMLAVPQIPSYRLNKICSG
LSFQRKNQFMQRLHNYWLLKRQARNGVPLIRRLHSHLQSQRNAEQREQDEKTSAVKEELKYWQKLRHDLE
RARLLIELIRKREKLKREQVKVQQAAMELELMPFNVLLRTTLDLLQEKDPAHIFAEPVNLSEVPDYLEFI
SKPMDFSTMRRKLESHLYRTLEEFEEDFNLIVTNCMKYNAKDTIFHRAAVRLRDLGGAILRHARRQAENI
GYDPERGTHLPESPKLEDFYRFSWEDVDNILIPENRAHLSPEVQLKELLEKLDLVSAMRSSGARTRRVRL
LRREINALRQKLAQPPPPQPPSLNKTVSNGELPAGPQGDAAVLEQALQEEPEDDGDRDDSKLPPPPTLEP
TGPAPSLSEQESPPEPPTLKPINDSKPPSRFLKPRKVEEDELLEKSPLQLGNEPLQRLLSDNGINRLSLM
APDTPAGTPLSGVGRRTSVLFKKAKNGVKLQRSPDRVLENGEDHGVAGSPASPASIEEERHSRKRPRSRS
CSESEGERSPQQEEETGMTNGFGKHTESGSDSECSLGLSGGLAFEACSGLTPPKRSRGKPALSRVPFLEG
VNGDSDYNGSGRSLLLPFEDRGDLKPLELVWAKCRGYPSYPALIIDPKMPREGLLHNGVPIPVPPLDVLK
LGEQKQAEAGEKLFLVLFFDNKRTWQWLPRDKVLPLGVEDTVDKLKMLEGRKTSIRKSVQVAYDRAMIHL
SRVRGPHSFVTSSYL

Canonical Epc-N
PHD 4.3e-08
Bromodomain 2.2e-35
PWWP 6.4e-28

>DrerioCAI20717
GPPMKRKSQPLNIFTPHREPLTYAQAQRMVDLEVDGQLHRISIFDKLEIITDEDPLAQEIIKCISSKENA
EKHLQISEYSAQIKNNNQKKNTSDSSIQGSILPEPKFHTVDYNLPVIPSRPSTYYKYKQKTVEELDQEVE
YDMDEEDFAWLDLVNEKRKDEGLNLVSFDDFEFLLDRFEKESFLESQGQHTLQSIDDEDAVCCICMDGDC
MDSNVILFCDMCNLAVHQDCYGVPYIPEGQWLCRHCLHSPTQPANCILCPNKGGAVKQTEDDRWGHVVCA
LWVPEVGFSNTVFIEPIDGVSNIPPARWKLTCYLCKEKGVGACIQCHKANCYTAFHVSCAQKSGLFMKME
PIKVLTDSGIPTFSVKKTAYCGAHTPNGSVKRPLAIYKDNETNYGLCQLVDRKSSRIGFNKQKRLKKNEE
AIVTTIPPLSVPSFSSERLSPILNQVSLQKKTAFGELVLSYWTLKRQSRNGVPLNRRLQTRLHPPKNSQR
KQTEEETQALKEQLKEWHQLRHDLERARILLELIRKREKLKREEVLLQQTLMEFQLTPFTVILRAVLDQL
QEKDQAHIFAEPVNIKEVPDYMDHVLHPMDFSTMSKRIEAQGYKYLDEFEADFNLITDNCMKYNGKDTFF
YRAAVKLRDQGGAILRKTRRDVQRIGLDLETGLHLLEPPKIEPSVPFSWEDVDRLLVPANREHMSFEEQL
RELLEKLDMTTAMKSSPSRSRRLKLLKKNISDVRLELSLRKALTAVKLKNAAEEEVGHIHQKGDKSVPPK
LEPSVSVLPLINAESHSEPPTLKPIEPNPDKHSHQKDGISQSLNGDSHLLLEDSDINVVATSTLAEPSSP

VNRRTSVLFRKSKSSTSPLRTLKDDETLKGSSQIGTKTFLSVVIPRLETLLHTRKRPRSASGDSCKDELP
VKRLDTELSNSFGLNSERELSSSSQPRQRCTSESSMSSSNSSIALNLTKCGKGKPALIRRNTVEDKSEII
ACIESRNFAKAARIAAEVNSSSIWMPSNAATVLLEPLKVVWAKCSGYPSYPALIIDPQMPRVGCQHNGVS
IPMPPLNVLQIGEQMQYKSEEKLYLVLFFDNKRTWQWLHKSKMVPLGVDKTIDKIKMMEGRTSAIRKAVR
TAFNRAMNHLNHVQDEPVNDFSDVD

Canonical Epc-N
PHD 1.2e-09
Bromodomain 2.6e-29
PWWP 4.4e-21

>AgambiaeEAA03942
MGIDFEVNDYIKTLKKDGGPFKCPMCDKTYKSVIGLQYHLNNYDHDNPSPAVPASVSTPIKQKGRKLKVG
VTPKNQIACSPPKEGITYLESEHLVRLDCNGKTVKIPVLDALPVMSLEEYESKCKTASDFEAFAVAPPEE
PEVQLPEGKFKEIEDYTICDAPARPNAYIRFIEKSSEELDGEVEYDVDEEDTTWLSIINERRAAQNVGPV
SVDSLELLMDRLEKESFFQAAANGQNGAVVDDDAVCCICMDGECQNTNVILFCDMCNLAVHQDCYGVPYI
PEGQWLCRRCLQSPSRPVDCVLCPNTGGAFKQTDHNQWAHVVCALWIPEVRFANTVFLEPIDSIETIPAA
RWRLTCYICKQKGIGACIQCNKTYCYAAFHVTCAQQAGLCMRMDTVKGTDSNPVVVQKTAYCDAHTPLNA
LQTTPGNSPDGGPPTDAREVTREKMKKARKLLALKRTSAPVILIPTIPQNRIEEISSLVNIPKKQQFIQR
LIAYWTLKRQYRNGVPLLRRLQSQGQAQGTGMPGCRDRTDGSPDAQELYQQLKYWQCLRQDLERSRLLCE
LVRKREKIKLILIKTTEQLVMAQLNPIESVLHRILDQLEVKDDKEIFREPVDTEEVPDYTDIVKHPMDLG
TMRQKLKRGAYIRIEDLEQDFQLMIRNCLAYNNKDTMFYRAGVRMRDAGAIVFRTVRKELERSGQFQEPA
PVEPIASSALSGRAIGSSSEDNIAMDIENELTKITGQSANAEHIGKLQTLLSKANGIRHALTRSKRIKQI
RNEIARVKRAISKDPERASSSFSPKKLSHSMHAEPTSSGLGGLAGSSLALAQKHHQITPEASPLKVLQNS
PSPSGVNRRTAVLFTRKAQAALKKPDASLKQEGAGVGASGGTGGAGGPSGSTVTPMTTTELLQKTAKKIN
RGKRIGKSGKGIESFLEATSASALDGKPIERKSLEAIPDSFRVYRGQQDREISDSESNLSLTGSTCSSCS
GFSGSGTESEFGGRRTSACGEGFEATNFVCLLSYGLALAPAMTIRTVTRMSTNSEPEVEPFPEKPALEPL
KLVWAKCRGYPWYPALIIDPNIPKGFVHNGVPLPAPPADVLALRSNYDEPVFLVLFFDVKRTWQWLPVGK
LELLGVDKELDQSKLIESRKPTERKAVNKAYQEALHYHSQV

C2H2 0.0027
Canonical Epc-N
PHD 2.3e-11
Bromodomain 3.9e-29
PWWP 2.9e-17

>PtroglodytesXP_518433
MTDRSRVRDPCLSQLDPNVHSPLIGLAPWRAGPWCAGIAAGADPVSCSVSPPRPHRHRLSPQFPGAMRKP
RRKSRQNAEGRRSPSPYSLKCSPTRETLTYAQAQRIVEVDIDGRLHRISIYDPLKIITEDELTAQDITEC
NSNKENSEQPQFPGKSKKPSSKGKKKESCSKHASGTSFHLPQPSFRMVDSGIQPEAPPLPAAYYRYIEKP
PEDLDAEVEYDMDEEDLAWLDMVNEKRRVDGHSLVSADTFELLVDRLEKESYLESRSSGAQQSLIDEDAF
CCVCLDDECHNSNVILFCDICNLAVHQECYGVPYIPEGQWLCRCCLQSPSRPVDCVLCPNKGGAFKQTSD
GHWAHVVCAIWIPEVCFANTVFLEPIEGIDNIPPARWKLTCYICKQKGLGAAIQCHKVNCYTAFHVTCAQ
RAGLFMKIEPMRETSLNGTIFTVRKTAYCEAHSPPGAATARRKGDSPRSISETGDEEGLKEGDGEEEEEE
EVEEEEQEAQGGVSGPLKGVPKKSKMSLKQKIKKESEEAGQDTPSTLPMLAVPQIPSYRLNKICSGLSFQ
RKNQFMQRLHNYWLLKRQARNGVPLIRRLHSHLQSQRNAEQREQDEKTSAVKEELKYWQKLRHDLERARL
LIELIRKREKLKREQVKVQQAAMELELMPFNVLLRTTLDLLQEKDPAHIFAEPVNLSEVPDYLEFISKPM
DFSTMRRKLESHLYRTLEEFEEDFNLIVTNCMKYNAKDTIFHRAAVRLRDLGGAILRHARRQAENIGYDP
ERGTHLPESPKLEDFYRFSWEDVDNILIPENRAHLSPEVQLKELLEKLDLVSAMRSSGARTRRVRLLRRE
INALRQKLAQPLPPQPPSLNKTVSNGELPAGPQGDAAVLEQALQEEPEDDGDRDDSKLPPPPTLEPTGPA
PSLSEQESPPEPPTLKPINDSKPPSRFLKPRKVEEDELLEKSPLQLGNEPLQRLLSDNGINRLSLMAPDT
PAGTPLSGVGRRTSVLFKKAKNGVKLQRSPDRVLENGEDHGVAGSPASPASIEEERHSRKRPRSRSCSES
EGERSPQQEEETGMTNGFGKHTESGSDSECSLGLSGGLAFEACSGLTPPKRSRGKPALSRVPFLEGVNGD
SDYNGSGRSLLLPFEDRGDLEPLELVWAKCRGYPSYPALIIDPKMPREGLLHNGVPIPVPPLDVLKLGEQ
KQAEAGEKLFLVLFFDNKRTWQWLPRDKVLPLGVEDTVDKLKMLEGRKTSIRKSVQVAYDRAMIHLSRVR
GPHSFVTSSYL

Canonical Epc-N
PHD 4.3e-08
Bromodomain 2.2e-35
PWWP 2.9e-27

>DpseudoobscuraEAL24777
MGLDFDALEYCKGMKNKQSPPPYACPVRDCGRSYKTIMGLQYHLVKYDHDNPQPLTPVLTPNRKKARSRS
THHSTPKPKDNGSGGDASIVSGEAKNGCSGSSGSGTRHQYANPESLVAYNEEEATVTFNIEGKSVRLGID
DALPMVEEDEFVALVERGCILNADAPPLEENAPWAKVQVPVAKVCEIEDYNVPDAPPRPLAYYRFIEKSL
EELDGEIEYDVDEEDSAWLEHMNEERQKLGLTAVSIDTMELLMDRLEKESHFQAAANGTPTGVEVDDDAV
CCICLDGECQNTNVILFCDMCNLAVHQDCYGVPYIPEGQWLCRRCLQSPSKPVNCVLCPNAGGAFKQTDH
GQWAHVVCALWIPEVRFANTVFLEPIDSIETIPPARWRLTCYVCKEKGLGACIQCHRNSCYAAFHVTCAQ
QAGLYMTMDTIKDGHNDSSMHVQKFAYCHAHTPADAKLKMNVPDFEDTRHKMREARKALAKKRSTAPVVL
IPTIPPDRVQEIATMVTMQKKKEFLDRIIAYWTLKRHYRNGVPLLRRLQSQGHNHGVIQRNGIEGSPDTN
ELYRQLKYWQCLRQDLERARLLCELVRKREKLKVAFVKISEEVVMLQLNPLESALTKLLDALEARDTMEI
FREPVDTSEVPDYTDIVKHPMDLGTMRTRLKDCQYTTLEQLETDFDLMIQNCLAYNNKDTVFYRAGIRLR
DQAAPLFVQLRKELQRDGLLERSQRSHVDHVEAEVEQELRQLLAVPASEDIVQKLLILADKSQVLKNPSY
RTKKIKQIRLEITRMRKSLQKARYAARHSSVHANRSRSDEDEARGGQGSPSKKRSRKRLNTIAMDMDLEQ
DEDLEDEDDSEEDSVGDGDEEAAKNMLNTTQTPPCSPIKSLNNSSSPVGINRRTAILLTRKSQAALKRPS
EPLTTPVKEEHHNSQSSSSQSISVSASASSSSNATVATAATSVASLNSVLAAPNASASVANFALTQNSSA
GAGVGAGVSASPASGAGALAGAAAAASLTSTALSISSKMSLNLGVKSPKRPGRYRRLPELRNSASMSPKK
SPNPAASGQAMPLPDARLFERIPDSFRVYRANNQRDVSDSDEGPSQSSSPCSSCSDFSISGSCSDFDSDE
ASDGHSEERESTSQDGTTDAMDLQHASLNNSQGNGNMAISSSSGASGSSSEDEAVMDGQTPHQKRRGRPS
KTRTSRSTPTPTPIPMAARGPVMSAARGRGKRRSNLSESTTSSTATPPPIMRKAGKLRSATPSIVSPLVN
NIKARRNTTATATTNNNRSRHGEDAEEVVTMTRHHNSHKPTLEPLQLVWAKCRGYPWYPALILDPKTPKG
FVYNGVPLPAPPTDVLALRKNCLDDIVFLVLFFDVKRTWQWLPANKLDILGIDKKVDQQKLIESRKPAER
KAVKKAYQDALHYQSQVSDLEGQGPDPIM

C2H2 0.0032
Canonical Epc-N
PHD 2.3e-11
Bromodomain 2.7e-31
AT hook
PWWP 3e-15

>TnigroviridisCAG01066 (misannotation?)
MKKKARHNRTPTLKRDSSPIKPSPNRETLTYAQAQRIVELEVDGRVHRLSIYDKLDVITDDDPTAQEILE
CNSNKENREKPPQVLVRSVRLKINRQKKNAALEASHGGGGSGTHGCPALLQPKFRTVEYNLPVVPKRPAA
FYKYTERLAEELDEEVEYDMDEEDYAWMELVNEKRRSEGIGQVSHNLFEFLMDRFEKESHSLTRGQSDLQ
AMVDEDAVCCICMDGDGADSNVILFCDSCNIAVHQECYGVPYIPEGQWLCRHCLQVRLLPQQRRSLKKTD
DGRWGHVACALWVPEVGFSDTVFIEPIDGVRNIPPARWKLTCYLCREKGAGACIQCDKVNCYTAFHVSCA
QKVGLYMKMEPVKEVLESGSATFSVKKTAYCCSHTPGTWSHRPLNVYEEPHPKHAACHRRGAKRGRARPK
SWGKKRSKRVEPEAEAETPPTSGPSITASSFEAILNQVAVQKKRSFVERVLSYWVQKRQSRNNVPLIRRL
QANPQPIRDSQTSRAETNQALKEQLKEWHRLRHDLERARLLLELIRKREKLKREQVKQQQSVLEFQLTPF
NVLLRAVLSQLQEKDKYSIFAQPVSTKEVVEDFNLIIDNCMLYNAKDTFFYKAAQRMQDHGGAILRRARK
EANRIGFDLPSGLHLAQSPRPEPAAPFSWEDVDRLLSPAYRQATPLEEQLKELLEKLDLSAAMKHSPSRS
KRLKLLKKSILEVRSELSLRKSFLPASPPRLAAPPQSEDQSPARPLSQPRPPPEEPLAPPVLELLTSLSQ
IDVPGECPTAPSPDVDAHTSTSALPDAPDGGAPASPLLDGELYAEASATCSQRANTLFRKSKSTSPQKAP
RSQEAGAVPPQPLGAKTFLSVVIPRLETLLLPGKRRRSIDSEQEEAEPVKRLGTGRVANGLAAEEELSAA
PRPLEPRRRCASESSISSGSGGLLPNTSAAFLTKSGKGRSPPARRSTVDDKNALRTCVEHRETPGSGESK
GLPVTTAPRRQKTAPRLRWAELPRPPVDVLRAGEQRRFRFAEKLFLVLFIGSRHSW

Canonical Epc-N
PHD 1.2e-11
Bromodomain 4.2e-10 (misannotation)

>CfamiliarisXP_538883 (misannotation?)

MATIQGCQCSHLFLVLRCKVVTCCHYTKIHFPWTIAQGAGGRRSQAPRWGRRRRAPGMSFTRKKGFYRQD
VNKTAWELPKTYVSPTHVGSGAYGAVCCAIDKRSGEKVAIKKLSRPFQSEIFAKRAYRELQLLKHMQHEN
VIGLLDVFTPASSLRSFHDFQDLTGSPHSVCTNLPRQLCRVPGVGLDLGALPGRKQGSGSPGHLWLVGQP
RVHGGFEHWGDVDRSDLGDLAGGKKQGAALGAEKACYLVMPFMQTDLQKIMGMEFSEDKIQYLVYQMLKG
LKVHGGREGGWGYIHSAGVVHRVSALSAVVLRGSSTLKGFSTDLKPGNLAVNEDCELKILDFGLARHADA
EMTGYVVTRWYRAPEVILSWMHYNQTDLDQLSQILKVTGVPGAEFVQKLNDKAAKSYIQALPQSPKKDFS
QLFPCASPQGESWGCPSGHLPSSPWAALPGLGCEVLSVAAATDLLEKMLELDVDKRLTASQALAHPFFEP
FRDPEEETEASQPFNDSLEHEKLTVDEWKRLIFENSLLGFWEEKMLGGQYTCEVVWLLLNRKRRGDQWAL
ASLPRWFAGASFLCVRVGLRVSRPLVRSPPPFRGGDVISLLSPRKGQDSSGRGAPLKGQGPWQGRSLSPE
SLVGYGGHRLIAETPRPSETWWGLSPWFPGAMRKPRRKSRQNAEGRRSPSPYSLKCSPTRETLTYAQAQR
IVEVDIDGRLHRISIYDPLKIITEDELTAQDITECNSNKENSEQPQFPGKSKKPSSKGKKKESCSKHGSG
TSFHLPQPSFRMVDSGSQPEAPPLPAAYYRYIEKPPEDLDAEVEYDMDEEDLAWLDMVNEKRRVDGHSVV
SADTFELLVDRLEKESYLESRSSGAQQSLIDEDAFCCVCLDDECHNSNVILFCDICNLAVHQECYGVPYI
PEGQWLCRCCLQSPSRPVDCVLCPNKGGAFKQTSDGHWAHVVCAIWIPEVCFANTVFLEPIEGIDNIPPA
RWKLTCYICKQKGLGAAIQCHKVNCYTAFHVTCAQRAGLFMKIEPMRETSLNGTIFTVRKTAYCEAHSPP
GAATTRRKGDSPGSLSEAGDEEGLKEGCGEEEEKEEVEEEEEDEGQGGVGGPLKGLPKKNKMALKQKIKK
EPEEVSRDTPSTVPMVTVPQIPSYRLNKICSGLSFQRKNQFIQRLHNYWLLKRQARNGVPLIRRLHSHLQ
SQRNAEQREQDEKTSAVKEELKYWQKLRHDLERARLLIELIRKREKLKREQVKIQQAAMELELMPFNVLL
RTTLDLLQEKDPAHIFAEPVNLSEVPDYLEFISKPMDFSTMRRKLESHLYRTLEEFEEDFNLIVTNCMKY
NAKDTIFHRAAVRLRDLGGAILRHARRQAENIGYDPERGTHLPESPKLEDFYRFSWEDVDNILIPENRAH
LSPEVQLKELLEKLDLVSAMRSSGARTRRVRLLRREINALRQKLAQPPPPPQPPSLNKTVSNGELPAEAQ
GDVTVLEQAPQEEPEDDGDRDDSKLPPPPTLEPTGPAPSLSEQDSPPDPPTLKPINDSKPPSRFLKPRKV
EEDELLEKSPLQIGSEPLQRLLSDNGINRVSLMAPDVPAGTPLSGVGRRTSVLFKKAKNGVKLQRSPDRA
LENGEDHGAVGSPASPASIEDEQHSRKRPRSRSCSESEGERSPRQEEETGVTNGFGKHTESGSDSECSLG
LSGGLAFEACSGLTPPKRSRGKPALSRVPFLEGVNGDSDYSSSGRSLLMPFEDRGDLEPLELVWAKCRGY
PSYPALIIDPKMPREGLLHNGVPIPVPPLDVLKLGEQKQAEAGEKLFLVLFFDNKRTWQWLPRDKVLPLG
VEDTVDKLKMLEGRKTSIRKSVQVAYDRAMIHLSRVRGPHSFVTSSYL

Pkinase 9.2e-35
PHD 4.3e-08
Bromodomain 2.2e-35
PWWP 2.9e-27

>MmusculusXP_196171
MGRGGDARPLPRSEGGDEAGKSARKGAAKRGPPPAPAILFLPGLPSWFPVAMRKPRRKSRQNAEGRRSPS
PYSLKCSPTRETLTYAQAQRIVEVDIDGRLHRISIYDPLKIITEDELTAQDITECNSNKENSEQPQFPAK
SKKPSSKGKRKESCSKHASGTSFHLPQPSFRVVDTGSQPEAPPLPAAYYRYIEKPPEDLDAEVEYDMDEE
DIAWLDMVNEKRRADGHSSVSADTFELLVDRLEKESYLESRSSGAQQSLIDEDAFCCVCLDDECHNSNVI
LFCDICNLAVHQECYGVPYIPEGQWLCRCCLQSPSRPVDCVLCPNKGGAFKQTSDGHWAHVVCAIWIPEV
CFANTVFLEPIEGIDNIPPARWKLTCYICKQKGLGAAIQCHKVNCYTAFHVTCAQRAGLFMKIEPMRETS
LNGTTFTVRKTAYCEAHSPSVAVARRKGDSPRSLSEGVSKKGKMSLKQKIKKEPEEAGREAPSITLPMVT
VPQIPSYRLNKICSGLSFQRKTQFMQRLHNYWLLKRQARNGVPLIRRLHSHLQSQRNAEQREQDEKTSAV
KEELKYWQKLRHDLERARLLIELIRKREKLKREQVKVQQAAMELELMPFTVLLRTTLDLLQEKDSAHIFA
EPVSLSEVPDYLEFISKPMDFSTMRRKLESHLYHTLEEFEEDFNLIVTNCMKYNAKDTIFHRAAVRLRDL
GGAILRHARRQAENIGYDPERGTHLPESPRLEDFYRFSWEDVDNILIPENRAHLSPEAQLKELLEKLDLV
STMRSSGARTRRVRMLRREINALRQKLAQPPPPQLLSLNKTVPNGELPAGSRGDTAVLEQAQQEEPEEEG
DRDDSKLPAPPTLEPTGPAPSLSEQESPPDPPTLKPISDSKPSSRFLKSRKVEDEELLEKSALQLGSEPL
QCLLSDNGIDRLSLTNPDSHPDTPLGTVGRRTSVLFKKAKNGVKLQRGPDGTLENGEDHGPEDDPASPAS
TEDEHYSRKRPRSRSCSDSEGERSPQQEEETGVTNGFGKHTESGSDSECSLGLSGGLAFEAGSGLTPPKR
SRGKPALSRVPFLEGVNGDSDHSGSGRSLLMPFEDHGDLEPLELVWAKCRGYPSYPALIIDPKMPREGLL
HNGVPIPVPPLDVLKLGEQKQAEAGERLFLVLFFDNKRTW

Canonical Epc-N
PHD 4.3e-08
Bromodomain 2e-33
PWWP 1.1e-09

>RnorvegicusXP_228039

MNFENIGVRRQVGDAQVQLCRIKGLSSSFPVAMRKPRRKSRQNAEGRRSPSPYSLKCSPTRETLTYAQAQ
RIVEVDMDGRLHRISIYDPLKIITEDELTAQDITECNSNKENSEQPQFPAKSKKPSSKGKKKESCSKHAP
GTSFHLPQPSFRVVDTGSQPEAPPLPAAYYRYIEKPPEDLDAEVEYDMDEEDMAWLDMVNEKRRADGHSS
VSADTFELLVDRLEKESYLESRSSGAQQSLIDEDAFCCVCLDDECHNSNVILFCDICNLAVHQECYGVPY
IPEGQWLCRCCLQSPSRPVDCVLCPNKGGAFKQTSDGHWAHVVCAIWIPEVCFANTVFLEPIEGIDSIPP
ARWKLTCYICKQKGLGAAIQCHKVNCYTAFHVTCAQRAGLFMKIEPMRETSLNGTIFTVRKTAYCEAHSP
SVATARRKGDSPRSLSEVGDEDGLKEGGGEEEQEEGEEEGQEGQGGVGSPLKGVSKKSKMTSKQKIKKEL
EEAGRETPSITVPMVTVPQIPSYSNVLPLVQSDINPGDSVSDVVAFTVTLSDIVDRPEPPTDLSLTPLCL
YPRLNKICSGLSFQRKNQFMQRLHNYWLLKRQARNGVPLIRRLHSHLQSQRNAEQREHDEKTSAVKEELK
YWQKLRHDLERARLLIELIRKREKLKREQVKVQQAAMELELMPFTVLLRTTLDLLQEKDPAHIFAEPVSL
SEVPDYLEFISKPMDFSTMRRKLESHLYHTLEEFEEDFNLIVTNCMKYNAKDTIFHRAAVRLRDLGGAIL
RHARRQAENIGYDPERGTHLPESPKLEDFYRFSWEDVDNILIPENRAHLSPEAQLKELLEKLDLVSAMRS
SGARTRRVRMLRREINALRQKLAQPPPPQLLSLTKTVPNGELPAGSRGDAAGLEQTLQEEPEDEGDRGLA
LRRGKALGSDGVEGLSSYNIWDAITNIYRSTQNKIYCQSRHQLLHWFVFLGTQMLQNDDSQLPAPPTLEP
TGPAPSLSEQESPPDPPTLKPISDSKPSGRLPKPRKVDDEELLGDSALQLGSEPLQCLLSDNGADRLPLT
NPDSPPGTPLGNVGRRTSVLFKKAKNGVKLQRGPDGTLENGEDAPASPASMEEEHCSRKRPRSQSCSDSE
GERSPQQEEETAKVSDLPLALVQQSELWDCGVGNSTDKQRKPPGQGVTNGFGKHTESGSDSECSLGLSGG
LAFEAGSGLTPPKRSRGKPALSRVPFLEGVNGDSDHSGSGRSLLMPFEDRGDLEPLELVWAKCRGYPSYP
ALIIDPKMPREGLLHNGVPIPVPPLDVLRLGEQKQAEAGERLFLVLFFDNKRTWQWLPRDKVLPLGVEDT
VDKLKMLEGRKTSIRKSVQVAYDRAMIHLSRVRGSHSFVTSSYL

Canonical Epc-N
PHD 4.3e-08
Bromodomain 1.4e-33
PWWP 4e-27

>GgallusXP_419262 (misannotation?)
MRKPRRRSRQKLEGRRSPSAYSLKCSPTRETLTYAQAQRIVEVDIDGRLHRISIYDPLKIITEDELTAQD
ITECNSNKENSEQPLFPSKSKKTPSKGKKREACSKHTSGTSLHLPQPNFRVVDSFKQSDAPPLPTAYYRY
IEKPPEDLDAEVEYDMDEEDLAWLEMVNEKRRDDGYGMVSAETFELLVDRLEKESYLESRNNGTQHSIID
EDAFCCVCMDDECHNSNVILFCDICNLAVHQECYGVPYIPEGQWLCRCCLQSPSRPVDCVLCPNKGGAFK
QTSDGRWAHVVCAIWIPEVCFANTVFLEPIEGINNIPPARWKLTCYICKQKGMGAAIQCHKVNCYTAFHV
TCAQRAGLFMKIEPMRETSINGTTFTVRKTAYCESHSPPGTVKKRYSAATSERQEGIVKEEREEEGSSGP
PKGSLKKNQVKLKQKIKKEPSEGTDGRSSMPMMTVAQIPSYRLNKICSGISLQRKNQFMQRLHNYWLLKR
QARNGVPLIRRLHSHLQSQRNAEQKEQDEKTSAVKEELKYWQKLRHDLERARLLIELIRKREKLKREQVK
VQQAAMELWLTPFNVLLRTTLDLLQEKDAAQIFAEPVNLNEVPDYLEFISNPMDFSTMRRKLESHLYRTL
DEFEEDFNLIVTNCMRYNAKDTIFHRAAVRLRDLGGAILRHVRRQAESIGFDTDVGIHLPESPKTEDFYR
FSWEDVDNILLPENRAHLSLEAQLKELLEKLDIVSPMRSSGARTRRMRLLRREINSVRQKLAQQQSRTMA
NGEPVLWEEELDKTSREEDEEGDDAKLPHPPTLEPTGPAPSFSELESLQDPPKLKPIHDSKSLNQLQKKV
MSDRDFFDKKALQRESQAFQRLLSDSSLNGLTLPPADTLMSPPFSGVGRRTSVLFKKAKNGVKLQKGLDC
SLENGEDHEQSEQLSPSCANGERQTRKRPQSRNYSESDGEKSPRQAGQRGVTNGFAKHAESGSDSEHSPS
LGSGLVFETCSGLMPPKRSRGKPALSRVPFLEGVNGDSDYSSSGRTLLMSFESQTELEPLELVWAKCRGY
PSYPALVGTQRREQLQICFLH

PHD 3.2e-07
Bromodomain 3.2e-35
AT hook
PWWP 8.8e-09 (misannotation)

>AmelliferaXP_395348
MVKEIDEYEQQLGEAEALPNSYIRFMERSGEELDGEVEYDLDEEDTAWLSIVNERRLASGLNPPLEPDTF
ELLMDRLEKESYFQQQSNGGGGIAADEDAVCCICMDGECQNSNAILFCDMCNLAVHQDCYAVDCVLCPNR
GGAFKQTDRPATWAHVVCALWIPEVRFANTVFLEPIDSIESIPAARWRLTCCVCKRRGSGACIQCHKSNC
YAAFHVTCAQQAGLCMRMRTVQPTNGEPMLVQKTAYCEAHTPSDYQPSTNPADARRRAIANKKSSSAPVI
SIPTIPPERIKEIASLAEGLPKRSQLIQRLIAYWTLKRQYRNGVPLLRRLQSSHPQSRPPPLGENSSPPP
DSELRGELYRQLKYWQCLRQDLERARLLCELVRKREKLKKELFKVKEKCLWFELRPLESILRSLLEAIKM
KDINDVFGQPVNTKEVPDYLEIVSHPMDLSTMQTKIERQEYDTIGAFEADFNLMVNNCLAYNRKDTMFYR
AGIKMKEQGGILIEQARKDYPELDPGENEQTVSKSRKRDRNNRSRGEIELQSGEKEIGGGGVNRRTAVLF

TRKARARASRSNQMFTLEDDKKKQSDSFKIYSKEIIANNGLEALQLVWAKCRGYPWYPALIIDPNTPRGT
VHKGVPIPAPPDDVLALAVNYKEPVFLVLFFDTKRTWQWLPGEKLEKLGVSQELDEAKLIESRKPADRKA
VKKAYQEALHYRKQTHNTSLNTASTS

Canonical Epc-N
PHD 0.0015
Bromodomain 1.5e-30
PWWP 7.8e-20

Epc-N SUBFAMILY IV

>GgallusXP_416870
MTGLNFELTTSTVPGHQRTWCQEERDHYSRSRGGLRVQVGNAAKTWRSLCLIIGVACPTAKATQATTSGG
GKGEGVPGVLLDSVVYRSCSEKARSVFLATILSSYQKTRRTFAAATISTNHCAGSSVLRSESDTALVAAD
LEKYWRSAPPRVSRQTAVLVAKPARAVGLEGPRGQNCSVSSATSCFTSLLGRCLSAVQKLRYAKPSPPRS
VLSSFLLLRTTVFARTFPQRPAESKSSGSGQHPRFGMPDVVSLSAEARAGGSPPTPPPPPLRRGAAREAA
GGLCGKGVKWTILGFYSEFVDSSQDLHGQFGTGVKTEKLLNIECRRRSPFVGCRLDCPPEGRGGVPGSHP
ATPVPQQRPVACLSCRPACVLRGTIRAPYLGCCFLCCSWYVCLDIGSSSFASFLLSNAVFPSSTQMYMCV
TPQEGIAGRNMPPLPMLLIAQSELVNQKEQLDALALMSSASYLEELCKADNKPHQQLHLCCRTLFDVNVF
VALGRLLLCKTHCCGSPVGLCPHFSALNTAESSSAGEGKDPAWGPEQPRACLCIGLIYIHPMVFSEIDSV
QIHVDFRFGAKGHLGEIRDVRAAGVERGSSPETVLKARVRFESTAKAPLLTFHESENYYLLILLQKSGCN
PALLLRGALTLGCVRMKRHRHLSTSDSSDNESPSTSFSSCAKYRSKSKTPANEQKKPAEVFRKDLISAMK
LPDSHHVNPDEYYVFADTWKQEWEKGVQVPASPETIPQPSLRVVAEKVKEVLYTRPRKYIHCSSQEPTEP
GYINILELAESVCRYDLDDMDIFWLQELNEELTEMGCGPLDENTMEKTIEVLERHCHENMNHAIETEEGL
GIEYDEDVICDVCRSPDSEDGNDMVFCDKCNICVHQACYGILKVPEGSWLCRTCVLGIHPQCLLCPKRGG
AMKATRTGTKWAHVSCALWIPEVSIACPERMEPITKVSHIPPSRWALVCSLCKLKTGACIQCSVKSCITA
FHVTCAFEHSLEMKTILDDGDEVKFKSYCLKHSKNKQNSLPDVDEHPKSISDQKQTESEKTSLRAQKLRE
LEEEFYSLVKVEDVAAELGLPKLAVDFIYNYWKLKRKSNFNKPLFPPKEDEENGLVQPKEDSIHTRMRMF
MHLRQDLERVRNLCYMVSRREKLKLSHSKVHEQVFNLQVQLINQEIAAGHTLPSALENTLFYPPPRITLK
LKMPKSALGDCKNNSLKPGNRPLSPDNNSTVYSKRSVPMSKEPFEIKAKSYSRYQHDTRSNGLLVTGGIG
KPRGEAKDSGLVQVPEFHRGQPSGKPLALQAALHGQSSIGNGRLQQDGSRLVAKSNGLMGRTGDVSQRDS
SSQTPFEQESVLTAHLASQSGFRKSTIEHFSRSFKEATNSLVRTTEDLRCCEKPTRRLAAKERLWSKQTL
EGAPYQDNDGYCPDLELSDSEAESDENKEQMRLRRSSSERESPSKDFGRECHNRNKKNMISHGSVQR

Canonical Epc-N
PHD 1.7e-16

>CfamiliarisXP_540950
MGMDQWVRSHGGVHGHGAVGVQSGAGAFGVHGHGQGRSRSSGCVVTEQWVRGHGAVGARSRGGGQSRGSG
CAVTGREGGVWGAQSRGVRGACGVRRHGAGAESWRRVVAAEGLSVPGRCQDKHRGKRGKSPGPARQTGTE
QTGMEQLVPGPGARVSRRSAQACRWLRPADAVTVVEDDRGKPAALAQHPRNATAYKNAYVSAPWDPLCSH
SEGLWHQPASLTGFWQQFPAQCRTCPSGGEAAADVSAGPRHRRSHAWALLASGLESFALFSGLIQGNTRV
VFTVGAGWVVSVPVQALIQLQLELLKPLSRCPVEDWPHGGRSSIGVFAGCFCLSTTWSQNSRSQHRRGSC
SRHEDRKPSERAQLTDAGVLRAKYNLGPALMPGSELPCVALRPWWPEDEAPLAGSAVLLSCPSRREQPCE
AAFGWSPLRPDEGRGAPSGQGYFEFCGSVFGRERRGEHPRGKYQQPSFELGAHRISGEETEVSLLLSDML
PACGVEVFRTDLITAMKLHDSYQLNPDEYYVLADPWRQEWEKGVQVPVSPGTIPQPVARVVSEEKSLMFI
RPKKYIVSPGSEPPELGYVDIRTLADSVCRYDLNDMDAAWLELTNEEFKEMGMPELDEYTMERVLEEFEQ
RCYDNMNHAIETEEGLGIEYDEDVVCDVCQSPDGEDGNEMVFCDKCNICVHQACYGILKVPEGSWLCRTC
ALGVQPKCLLCPKKGGAMKPTRSGTKWVHVSCALWIPEVSIGSPEKMEPITKVSHIPSSRWALVCSLCNE
KFGASIQVLQRVPFAEAVIRTQVGYLIRPGFHHVWGPEWGGGNGFVKTACVFRVHTTHRQVSRILRFGLK
LKRGKTLRYLWERMEETLFKNLRVKAQALGPCKRVCVPTYPVSTCRTAAEGSGSSCLPWHRRPLRIEFLP
FLSTPVLPRHRQRDVGACEGSGLGFRNICLLSQPEEENPEEGRLAPLHSPVEVPPPFVGPGPPEEGSLGF
SAAVLAAGARPPAGPRLLSGDWLQWPQGASSRSPRQAEGAVVSGRAQPPCDHRKRPFRVLRPGRGQRPGG
PGPEAFPRAPGGAAWPSAPRGGLSIPPPGLASLLLPFFPPLPGVSYSLSSDLSSPRLLGGNCSVKNCRTA
FHVTCAFDRGLEMKTILAENDEVKFKSYCPKHSSHRKPEESLGEGAAQENGASECSPRNPLEPFASLEQN
QEEAHRVSVRKQKLQQLEDEFYTFVNLLDVARALRLPEEVVDFLYQYWKLKRKVNFNKPLITPKKDEEDN
LAKREQDVLFRRLQLFTHLRQDLERVMIDTDTLAAADGTSSTADSGSGSPLPAAGILGAGCLPSSGASCP
RSFWQRLANWGGVSEQPTFSSLVLGSGDPGPLSGLTHPAPKGTQGMSQGMSQGMSQGMSQGMSQGMSQGM
SGPSRGADGTVPNEGSVQKGGRASGAPFLHGVRNLTYMVTRREKIKRSVCKVQEQIFNLYTKLLEQERVS
GAGPSLSPAFVAYCKQPARKAAGCRSPASRAAAVQAGPVEGRSVALVHTEEETQKCGFVKVDPKHSTGSG
GNSLPKQPDLCGRREGTVVPEGFLSFEKTFAEARLVSAQQKNGVVMPDSGDRRDHRLHCDLSRGDFKDRP
CKQSHKPLRPTDPSQRHLDSPRAAAASPGTGQSAPGSRKEIVPKCNGSLIRVNYNQTAVKVPTTPASPVK
NWGGFRIPKKGERQQQGEAHDGACHQHSDYPYLGLGRVPAKERAKNKLKSDHENDGYVPDVEMSDSESEA
SEKKCIHASSTISRRTDIIRRSILAS

PHD 7e-16

>TrubripesCAE30495

MKRLRSSSSSDSSDNESPSTSFCSSNKNGGKPGTPASNPKKPAEVFRKDLISAMKLPDSHHVPPEDYYLL
ADTWRQEWEKGVQVLASPDTIPEPSARVIAERPTEVLYSHQRKYIQSSNQESTEPGFVNIRELAEAMCRY
DLDDVDLYWLNMLNKELERMGEEPIDELTMERTIEALERHCHDNINHAIETVEGLGIEYDEDVICDVCRS
PDSEEGNDMVFCDKCNICVHQACYGIVKVPFGNWLCRTCVLGITPQCLLCPKKGGAMKATRAATKWAHVS
CALWIPEVSIACPERMEPITKVSHIPPSRWSLICSLCKLKTGACIQCSVKNCTTPFHVTCAFEHNLELKT
ILDEGDEVKFKSYCLKHSQGKTGEAGLSPARSKPPAEAVKVGLRAQRLQELEEDFCALVQPGEVSQELGL
PERPADFIFQYWKLKRKANFNKALLPPKEEEENVVLQPQEDSIHTRMRMFMHLRQDLERVRNLCYMVSRR
EKLKLLQSKAQEQMFNLHVALVNQELSAGLQPSCLMESMLFHPAPRITLKLKMPKSSSVGNGNPAFKSGN
GPLCPDNSGNVADQPGGGLGQGKPQLHENGRGEERPNNGQLSSSSNSASAVRFTGKPLALHAALHGHSSK
GRRKPDQERPRVSKSNGLYEKIVSQKNKSSEPPCDQYASKKGVSKSSFHKSAMEHFGRPFKDATISLVRT
TEDRLASDKLSRDTAAKDRLWTKPVPEHKVKSSRSYQDSDGYCPDLELSDSEPEAKGRRGQQVERAHLEP
PRKGISHGKKTLGSRHAMQR

Canonical Epc-N
PHD 3.5e-15

>TnigroviridisCAG01391
MSWTGVRRAVPPALARTGAGYRSRTRRDAGPLPGGDCGLQPLQEGTSLCPLAPMFGSSPARSAKGPAASA
SLAPTWFSGTGSLLYFNPLLFFLSKSILKGLVAAVSSRCCSPASPPALSAPPDGSAFRTGALTLAGAAVA
PGWSRMKRLRSSSSSDTSDNESPSTSFCSSNKNGSKPGTPASNPKRPAEVSSRLQDVCGGEVEDVVTASE
KHTLQKKRGRSQFCCFLKVFRKDLISAMKLPDSSHVPPEDYYLLADTWRQEWERGVQVPASPDTIPEPSA
RRSTSSAGARSPQNRGSANIRGLAEAMCRYDLDDVDLYWLHALNTELERMGEEPVDELTMERTMEALERH
CHDNINHAIETVEGLGIEYDEDVVCDVCRSPDSEEGNDMVFCDKCNICVHQVAQSPAEEPDVARETNQLL
SLLSRSPPGLLRHCQGALWELAVQDLRARHQPPVPALSQEGRRHEGHTRRHQMGARQLCSVDTRGPIQAA
GVLVGRRSTGDTFSPLQVSIACPERMEPITKVSHIPPSRWSLICSLCKLKTGACIQCSVKNCTTPFHVTC
AFEHSLELKTILDEGDEVKFKSYCLKHSQGRAGEATLSPARSKPPAQAEKVGLRAQRLQELEEDFYTLVQ
LGEVSRELGLSERPADFIYQYWKLKRKSNFNKALLPPKEEEENVVLQPQEDSIHTRMRMFMHLRQDLERV
RNLCYMVSRREKLKLLQSKAQEQMFDLHVTLLNQELSAAAASMSVCGSSAPSPGELGQLPMDSWMAHLPC
ALWDVPLSHLAIPGSHNAITYCLDMNERSPVDLTQPDMLQKLDKYMKPLIRPFVYKWAITQEYPMKQQLD
CGVRYCDLRIAHRPNDSSTDLYFYHGVYTTLTVEVGVYKHSPSCRVAVFAMVTAFCEHQQMLQTSVLMEM
REWLDAHPKEVLILSFSHFLGLSQELHALLLTTIRNIFCSKLCPKTRWANKCKAETLIEAFEHRKKQDRP
EGLFVTGINLTVDLKYICTHPTESLKDLVMSTYSVLLGWVREQTPGSRVGSVNIIAGDFVTERHFAPTVV
TLNEKLLKRSS

Canonical Epc-N
PHD 3.2e-09 (misannotated?)

>DrerioCAE30492
MKRLRTPSSSDSSDNESPSTSFSSNKYGSKPGTPASAQKKPAEVFRKDLISAMKLPDSHHISSEDYYLLA
DTWKQEWEKGVQVLASPDTIPQPSVRIITEKPKEVLFSKPRKYIQCWSQDSTETGYVNIKELAEAMCRYD
LDDMDLYWLQQLNAELGMMGDGVVDELTMERVMEALERQCHENMNHAIETEEGLGIEYDEDVICDVCRSP
DSEEGNDMVFCDKCNICVHQACYGIVKVPDGNWLCRTCVLGITPQCLLCPKTGGAMKATRAGTKWAHVSC
ALWIPEVSIACPERMEPITKVSHIPPSRWSLICSLCKLKTGACIQCSVKNCTIPFHVTCAFEHSLEMKTI
LDEGDEVKFKSYCLKHSKPKAGEPGLSPARHKPPTETDKLSLRAQKLQELEEEFYTYVHPEEVAHDLSLP
LHLLDFIFQYWKMKRKSNFNKALLPPKEDEENLLLQPQEDSIHTRMRMFMHLRQDLERVRNLCYMVSRRE
KLKLSQSKAQEQIFNLHVKLVNQELSAGLPVSSSIENLLFHPPPRITLKLKMPKVQLGNGKSSSKSGNGP
LCPDNSCNLYDTSEGGIGQGKPQLHLGRQRIEERINGILPASIYIRRDGGTPPLAVKQSGKPLALHAALH
GQSSNGKTKNEAEKSRQIKSNGILDKPILQRDTSCLAASEKDPRSEISGKSQSSGFHKTSLEHFSRSLKE
ATVSLVRTEDLRTFEKNSRKSSGFSKPLSTERPQGGGRASQESDGYCPDAELSDSEPEAKGKCRQGGRTQ
NQRGDYVKSASRAKHSYGSRTSVQR

Canonical Epc-N
PHD 4.3e-17

>RnorvegicusXP_227074
MSWVTTPKDPRRLRGILACGKRDPLPGDLSAWAQLFPVCTLAVTQHQGPPRVPFRDGEELVRLEQLATPF
SRLVCLISLNCGPLPHRTAFISNLRAGDSRGSPGSPRGLCGPDSCGAACSPRRPSPCAAGCLLSPGEIMK
RGRLPSSSEDSDDNGSLSTTWSQHSRSQHGRSTCSRHEDRKPSEVFRTDLITAMKLHDSYQLNPDEYYVL

ADPWRQEWEKGVQVPVSPGTIPQPVARVVSEEKSLMFIRPKKYIASSGSEPPELGYVDIRTLADSVCRYD
LNDMDAAWLELTNEEFKEMGMPELDEYTMERVLEEFEQRCYDNMNHAIETEEGLGIEYDEDVVCDVCQSP
DGEDGNEMVFCDKCNICVHQACYGILKVPEGSWLCRTCALGVQPKCLLCPKKGGAMKPTRSGTKWVHVSC
ALWIPEVSIGSPEKMEPITKVSHIPSSRWALVCSLCNEKFGASIQCSVKNCRTAFHVTCAFDRGLEMKTI
LAENDEVKFKSYCPKHSSHRKPEESLGEGAAQENGATECSPQSPLEPFASLEQNREEAHRVSVRKQKLQQ
LEDEFYTFVNLLDVARALRLPEEVVDFLYQYWKLKRKINFNKPLITPKKDEEDNLAKREQDVLFRRLQLF
THLRQDLERVRNLTYMVTRREKIKRSVCKVQEQIFSLYAKLLEQGKVSGVPSSCSSLENMLLFNSPSVGP
NVPKIEDLKWHSAFFRKQMGTNLVHSLKKSHKRDALQNSSGTEGKTLLKQPGLSGRREGSGVPESLLSFE
KTFAEARLISSAQQKNGVVTPDHGKRRDNRFHCDLVKGDLKDKSFKQSHKPLRSTDTSQRHLDNTRAATS
PGVGQSAPGTRKEIVPKCNGSLVKVPTTPASPVKSWGGFRIPKKGERQQQGEAHDGACHQHSDCSHLGIS
RAPAKERSKSRLRTDSENDGYAPDGEMSDSESEASEKKCIHASSTISRRTDIIRRSILAS

Canonical Epc-N
PHD 7e-16

>GgallusXP_420467
MGADNRSPVRGTGKHQHQGTRRNGSPGGYRGSCYTSVQTEPPELLCWTQKDVALALLVGRQQPRGSRRCR
FIRAPAVIRYGRALPNSRVPRFPRLKTAEPRGCREPGPRHKPCAEPARRAPGERSGRESKAAHAYKPEAF
TKRCRNARCDSVRRGFSPQRDVGTARLCAQQQRGFPSGCTHSLLAAFELCFAAAENVQRFLSCFLPAVHP
AAGPSGAVRADSSMEAGRNRGGCRGWSRGAALSGSGLSEGRSWPGNMLPLSNGTPRAFSPNLGFRAFYVL
RYFTSVFMGQCLDVCSEEARIKDLDFVPLQLSVCLKKRERKEFGIAVPCSFCCLSVVSNQSAVEAAVCVC
SRWSDGGTAWLAASGKHSSCEGSGKWFCGACGRSECFVGTPRLLSSYAVAISCWGLSSLQISHKAWRKMV
FFLESDIIASILSVCKLRMHVAEQGKPAPSDCTSPPAVCGCLLFPGEIMKRRRLPSSSENSDDNGSDFFT
YVHFQGASVLHGRAAQTALAVRSVPYTNPTQTIAEMRECEVPLLGEVPEYGHKAAKSNHTLHFPPALLLV
PLERVLCSLMEAALGLSTWSQHSRSHHRRSSCSRQEDRKPSEMTANPEVGKDAAGFRAKREVKLGSIIQH
HVSSVGGTPGLIEEVTQVIVSVFAPFATLFSAMKLNFCPAVGWVFRTDLITAMKLHDSFQLNPDEYYVLA
DPWRQEWEKGVQVPVSPGTIPEPVARIVSETKAVTFTRPRKYISSSGTKPPELGYVDIRTLADSVCRYDL
SDVDVAWLQLANEEFKEMGMPELDEYTMERVIEEFEQRCYDNMNHAIETEEGLGIEYDEDVVCDVCQSPD
GEDGNEMVFCDKCNICVHQRCLLAFALLFSGVNKFLVEEWTVFFCIAAGHDFCQRRACYGILKVPEGSWL
CRTCALGVQPKCLLCPKKGGAMKPTRSGTKWVHVSCALWIPEVSIGSPEKMEPITKVSHIPSSRWALICS
LCNEKVGASIQVDSGALGVSNGGKEEHQQKNNLLACSVKNCRTAFHVTCAFDRGLEMKTILAENDEVKFK
SYCPKHSSTKRADDETFSDHLCQENGNGIQDSSLPAHIDPFHSMDQNQEEAHRVSLRKQKLQQLEDEFYT
FVESLEVAKALQLPEELVGFLYQYWKLKRKANFNKPLITPKKDEEDNLAKREQDVLFRRLQLFTHLRQDL
ERVRNLTYMVTRREKIKRSVCKVQEQIFNIYAKQLEQERVSGVPSSFSTMENAVLFNSPSLGPNAPKIED
LKWHSAFFRKQMGTSLTHSLKKSHKRDGVSNSSGNSSKSMLRQPSQRGDGATPGSLLNFDKSFAETRIVS
AQQKNGIVMPDHRKRRDNLPQCEVIKTELKEKTKHNHKPLRPTELSQRQSENKRAVNHSSGRSAPGTRRD
IVPKCNGGLVKINSNQTEVKVPTTPTSPVKNWGGFRIPKKGERQQQGESQEETCRQNSGYRYLGVGRVSP
KDRAKSKPKPDSENDGYIPDAEMSDSESEVAEKKCRQQRLSPNSTISRRTDIIRRSILAS

Canonical Epc-N
PHD 2.2e-07

>DrerioCAE30491
MKRSRVPSTSEDSDNGSNSTSWSQHSNSKHRKQSGKRPSEVFRTDLITAMKLHDSHQLNPEDYYELADPW
RQEWEKGVQVPVSPESIPQCAVRTVAEKSTAPLFIKPKKLIRSSESSMLGYVGIQTLADGMCRYDLNEED
VAWLQITNEEFSKMGMQPLDELTMERVMEEFERRCYDNMSHAMETEEGLGIEYDEDVVCDVCQSPDGEDG
NEMVFCDKCNICVHQACYGILKVPEGSWLCRTCALGIFPKCHLCPKKGGAMKPTRSGTKWVHVSCALWIP
EVSIGNPEKMEPITNVSHIPSNRWALICCLCKEKTGACIQCSAKSCRVAFHVTCGLHCGLKMNTILTEAD
EVKFKSFCPKHSGLDWNEEEGDDDRPVKVPTREDRSRNRGIDFSASSQTRLSQNPEETRLSERKLRVQQL
EDEFYRFVAADEVAEHLQLPLEMVDILFQYWKLKRKVNFNQPLIMPKKEEEDSLARREQEVLLRRLRLFT
HLRQDLERVRNLTYMVSRRERIKRTLCRVQEQIFHHHVRLLEQGRVTGVSSTRRLEEAMFYFRTTPPVPA
SPQPLKGHCGQNSTLSSSEKGSNSYRSSKHIEADKPAKMLMDGVPSSGDSVRSETVMSASSRRSEGRTRS
GESHRKEEESERPLEDRRRKSKLWDQVSIKDKLRHAKSMEDTLSSETELDTMDDRLLLSHTNANSVATAP
NMYSGSPRKTNASHQGKLVPNGTSGRHLKNWGSFRIPKRSERTSAGRQTERQEADNTADQNSSLKTFSTS
PSSPQIRTRLRTGSENRRHLEESDLGQSEQGKRCHTQRLPSPMTRRYGSDVIQRGVLAS

Canonical Epc-N
PHD 7e-16

>HsapiensBAC86931
MKRGRLPSSSEDSDDNGSLSTTWSQNSRSQHRRSSCSRHEDRKPSEVFRTDLITAMKLHDSYQLNPDEYY
VLADPWRQEWEKGVQVPVSPGTIPQPVARPKKYIVSSGSEPPELGYVDIRTLADSVCRYDLNDMDAAWLE
LTNEEFKEMGMPELDEYTMERVLEEFEQRCYDNMNHAIETEEGLGIEYDEDVVCDVCQSPDGEDGNEMVF
CDKCNICVHQACYGILKVPEGSWLCRTCALGVQPKCLLCPKKGGAMKPTRSGTKWVHVSCALWIPEVSIG
SPEKMEPITKVSHIPSSRWALVCSLCNEKFGASIQCSVKNCRTAFHVTCAFDRGLEMKTILAENDEVKFK
SYCPKHSSHRKPEESLGKGAAQENGAPECSPRNPLEPFASLEQNREEAHRVSVRKQKLQQLEDEFYTFVN
LLDVARALRLPEEVVDFLYQYWKLKRKVNFNKPLITPKKDEEDNLAKREQDVLFRRLQLFTHLRQDLERV
RNLTYMVTRREKIKRSVCKVQEQIFNLYTKLLEQERVSGVPSSCSSSSLENMLLFNSPSVGPDAPKIEDL
KWHSAFFRKQMGTSLVHSLKKPHKRDPLQNSPGSEGKTLLKQPDLCGRREGMVVPESFLGLEKTFAEARL
ISAQQKNGVVMPDHGKRRDNRFHCDLIKGDLKDKSFKQSHKPLRSTDVSQRHLDNTRAATSPGVGQSAPG
TRKEIVPKCNGSLIKVNYNQTAVKVPTTPASPVKNWGGFRIPKKGERQQQGEAHDGACHQHSDYPYLGLG
RVPAKERAKSKLKSDNENDGYVPDVEMSDSESEASEKKCIHTSSTISRRTDIIRRSILAS

Canonical Epc-N
PHD 7e-16

>MmusculusCAE30496
MKRGRLPSSSEDSDDNGSLSTTWSQHSRSQHGRSSTCSRPEDRKPSEVFRTDLITAMKLHDSYQLNPDDY
YVLADPWRQEWEKGVQVPVSPGTIPQPVARVVSEEKSLMFIRPKKYIASSGSEPPALGYVDIRTLADSVC
RYDLNDMDAAWLEVTNEEFKEMGMPELDEYTMERVLEEFEQRCYDNMNHAIETEEGLGIEYDEDVVCDVC
QSPDGEDGNEMVFCDKCNICVHQACYGILKVPEGSWLCRTCALGVQPKCLLCPKKGGAMKPTRSGTKWVH
VSCALWIPEVSIGSPEKMEPITKVSHIPSSRWALVCSLCNEKFGASIQCSVKNCRTAFHVTCAFDRGLEM
KTILAENDEVKFKSYCPKHSSHRKPEEGLGEGAAQENGAPESSPQSPLEPYGSLEPNREEAHRVSVRKQK
LQQLEDEFYTFVNLLDVARALRLPEEVVDFLYQYWKLKRKINFNKPLITPKKDEEDNLAKREQDVLFRRL
QLFTHLRQDLERVRNLTYMVTRREKIKRSVCKVQEQIFTQYTKLLEQEKVSGVPSSCSSALENMLFFNSP
SVGPNAPKIEDLKWHSAFFRKQMGTSLVHPLKKSHKRDAVQNSSGTEGKTSHKQPGLCGRREGLEVSESL
LSLEKTFAEARLLSSAQQKNGVVTPDHGKRRDNRFHCDLVKGDLKDKSFKQSHKPLRSTDTSQRHLDNTR
AATSPGVGQSAPGTRKEIVPKCNGSLVKVPITPASPVKSWGGFRIPKKGERQQQGEAHDGACHQHSDCSH
LGVSRAPAKERAKSRLRADSENDGYAPDGEMSDSESEASEKKCIHASSTISRRTDIIRRSILAS

Canonical Epc-N
PHD 7e-16

>HsapiensCAE30500
MKRGRLPSSSEDSDDNGSLSTTWSQNSRSQHRRSSCSRHEDRKPSEVFRTDLITAMKLHDSYQLNPDEYY
VLADPWRQEWEKGVQVPVSPGTIPQPVARVVSEEKSLMFIRPKKYIVSSGSEPPELGYVDIRTLADSVCR
YDLNDMDAAWLELTNEEFKEMGMPELDEYTMERVLEEFEQRCYDNMNHAIETEEGLGIEYDEDVVCDVCQ
SPDGEDGNEMVFCDKCNICVHQACYGILKVPEGSWLCRTCALGVQPKCLLCPKKGGAMKPTRSGTKWVHV
SCALWIPEVSIGSPEKMEPITKVSHIPSSRWALVCSLCNEKFGASIQCSVKNCRTAFHVTCAFDRGLEMK
TILAENDEVKFKSYCPKHSSHRKPEESLGKGAAQENGAPECSPRNPLEPFASLEQNREEAHRVSVRKQKL
QQLEDEFYTFVNLLDVARALRLPEEVVDFLYQYWKLKRKVNFNKPLITPKKDEEDNLAKREQDVLFRRLQ
LFTHLRQDLERVRNLTYMVTRREKIKRSVCKVQEQIFNLYTKLLEQERVSGVPSSCSSSSLENMLLFNSP
SVGPDAPKIEDLKWHSAFFRKQMGTSLVHSLKKPHKRDPLQNSPGSEGKTLLKQPDLCGRREGMVVPESF
LGLEKTFAEARLISAQQKNGVVMPDHGKRRDNRFHCDLIKGDLKDKSFKQSHKPLRSTDVSQRHLDNTRA
ATSPGVGQSAPGTRKEIVPKCNGSLIKVNYNQTAVKVPTTPASPVKNWGGFRIPKKGERQQQGEAHDGAC
HQHSDYPYLGLGRVPAKERAKSKLKSDNENDGYVPDVEMSDSESEASEKKCIHTSSTISRRTDIIRRSIL
AS

Canonical Epc-N
PHD 7e-16

>MmusculusBAC33688
MKRGRLPSSSEDSDDNGSLSTTWSQHSRSQHGRSSTCSRPEDRKPSEVFRTDLITAMKLHDSYQLNPDDY
YVLADPWRQEWEKGVQVPVSPGTIPQPVARVVSEEKSLMFIRPKKYIASSGSEPPALGYVDIRTLADSVC
RYDLNDMDAAWLEVTNEEFKEMGMPELDEYTMERVLEEFEQRCYDNMNHDIETEEGLGIEYDEDVVCDVC
QSPDGEDGNEMVFCDKCNICVHQACYGILKVPEGSWLCRTCALGVQPKCLLCPKKGGAMKPTRSGTKWVH

VSCALWIPEVSIGSPEKMEPITKVSHIPSSRWALVCSLCNEKFGASIQCSVKNCRTAFHVTCAFDRGLEM
KTILAENDEVKFKSYCPKHSSHRKPEEGLGEGAAQENGAPESSPQSPLEPYGSLEPNREEAHRVSVRKQK
LQQLEDEFYTFVNLLDVARALRLPEEVVDFLYQYWKLKRKINFNKPLITPKKDEEDNLAKREQDVLFRRL
QLFTHLRQDLERVRNLTYMVTRREKIKRSVCKVQEQIFTQYTKLLEQEKVSGVPSSCSSALENMLFFNSP
SVGPNAPKIEDLKWHSAFFRKQMGTSLVHPLKKSHKRDAVQNSSGTEGKTSHKQPGLCGRREGLEVSESL
LSLEKTFAEARLLSSAQQKNGVVTPDHGKRRDNRFHCDLVKGDLKDKSFKQSHKPLRSTDTSQRHLDNTR
AATSPGVGQSAPGTRKEIVPKCNGSLVKVPITPASPVKSWGGFRIPKKGERQQQGEAHDGACHQHSDCSH
LGVSRAPAKERAKSRLRADSENDGYAPDGEMSDSESEASEKKCIHASSTISRRTDIIRRSILAS

Canonical Epc-N
PHD 7e-16

>AnidulansEAA57618
MAPYTSPKRSSPGGPLKNGSKTPLLDSGNSETPVADNLGPIIKKRKYVPGGPGGGGRYIEIEVRETPKQP
KPKPKPKQPKPSPVKRTSSSISRTRHQLREAESSAQPAQLPPPPPPVHAPTTPPSTRLRREKSQTRGRFG
SSTAAALALQQGDGYKPREERGWEEFHPELDIDAQFPVFPSAEVDRPPLSAQLASILSPNGLDKTNENDP
VAELIRAHANGTVQTPIKRRPGRPPRRPEAILQSLGVFTPQPKVVPPPGPNPREKLTLPKPSFRLKDPFT
FYDQPGVGQQNYVDRTMASVGYQESDLFIRHDRRLIRMPEGAQEDDLDIGNPLLGEGEVNAAVGRVEYDM
DEQDEKWLEDYNAKRREDQYEPIKPAVFEITMTKIEKEWHSLEKRIPKPNPKPPQTQRPRSSSAAAVNGE
TTGPGEDQDTKCAICDDGDCENANAIVFCDGCDLAVHQECYGVPFIPEGQWLCRKCQLIGRGSPNCIFCP
NIEGAFKQTTTSKWSHLLCAIWIPEVSIGNPSLMEPITDVEKVPRSRWKLQCYICRQKMGASIQCSNKNC
FVAFHVTCARRAQLYLKMKSGHGNLAVMDSHLLKAFCDKHVPPEWRREHGTDAATADAIEYYRTTMQGRR
WGDSQAAALSLEPAHPLGGGPEDEDGQRTHTPRITLTVGGNKRKRPNAPRTIWKLPSGAPVIPQVLLNSV
VASLQRFGVRQRKQYAEDACKYWTLKREARRGAALLKRLQLQLETFSSMEMTRRDYAAMGATGSKRLQRR
IEFGDRLYRDLDRLRMLCDEVKKREREKLKDAEMLRNIVDTVYFPIFPLLWPIFEKAQVLDSKGFFRRGL
LAIRTKLEQRYYPSVSSFSAHLASIFTSEIGVQPAGDTAELQMQISGRAPELSLEQREKRKLAKRIIKAI
QPALEDAIRKESELTRQPFEQELKDLDVMLGDVSSGRGSEIDSAERPGEEAFEKSELPNGDTDSTNMDPN
STAKMKSTEEVLESTETNTPGAEPSKLTGSQAAQAAATENTPVDVPMSDASTTQPPNEAQAAITPELAEE
PGEAPVIQQQTNQTPQVPLSPPALETDQHHPLARGGIQWYMQPFDPMGTTIHEERWTGREVMRGLSEELS
ELDEEELNDLVDEDELEENATGTGVFANRAPDSSRGEQGVKVHRTRRRWRGFK

Canonical Epc-N
PHD 1.2e-12

>TnigroviridisCAG03789
MKRSRHHSSSEDSDNGSSSTCWSQHSTQPRRSAGQKPSEVFRTDLITAMKVHDSYQLNPEDYYVLADPWR
QEWEKGVQVPVSPQSIPQPVVRTLAEKEKEVMFVRPKKLIRTSGAEALGYVDIRTLAEGMCRYDLNEQDV
AWLQIANKQFAEMALPPLDEITMERVMEEFERRCHENMTHAMETEEGLGIEYDEDVVCDVCQSPDGEDNN
EMVFCDKCNICVHQACYGIQKVPKGSWLCRICALGILPKCQLCPKKGGAMEADPEWNQVSIGNPEKMEPI
TNVSQIPSNRWALVCCLCKEKTGACIQCSAKNCRTAFHVTCGLHASLRMKTILTEDDEVKFKSYCPKHSG
LDGAQVRDSGGEEESARDKKRRTGRTRGEEEEEEEEEEEEEEEEEEAAPASLASQVSSRTSGAADALSSH
QQEKRVNLRKLKLEAMEEEFYRFVQAEEVARHLKLSPEPADFLYQYWKLRRKANFNQPLLTPKKDEEDSL
ARREQEVLRCRLQLFTHLRQDLERVRNLTYMVTRREKMKRSMWKVQEQIFQHQVHLLDHELLTGDPSAKD
LENLVFLGWFSSPDPRSRSSCAPSALKPKQGPLKVEKRKLSARKGLDSPEAPRRDGPSRDGRGAQNGGSG
SESESKSREEIPSPELREPEASQEEAKLCRLESRKAPRKEAPGPTQEDLVGQEPEEDQEERRKKKRSDMM
DRFRFKHLEKSVSIRLVDIRNSDIGGAARGPFRSPVLLSDVAVGNRANGWLKKPAAGGRAAAAGHLRSWG
KFRIPKRNEKTAAEEPPLLPNASSQPRTRLRTGAENAGYSPTAGEGEVEPCLKRCHSHQLRGASALGRRY
GSDIIRRGVLAS

Canonical Epc-N
PHD 3.6e-15

>PtroglodytesXP_517435
MKRGRLPSSSEDSDDNGSLSTTWSQNSRSQHRRSSCSRHEDRKPSEVFRTDLITAMKLHDSYQLNPDEYY
VLADPWRQEWEKGVQVPVSPGTIPQPVARVESLLTLLTIRPKKYIVSSGSEPPELGYVDIRTLADSVCRY
DLNDMDAAWLELTNEEFKEMGMPELDEYTMERVLEEFEQRCYDNMNHAIETEEGLGIEYDEDVVCDVCQS
PDGEDGNEMVFCDKCNICVHQACYGILKVPEGSWLCRTCALGVQPKCLLCPKKGGAMKPTRSGTKWVHVS
CALWIPEVSIGSPEKMEPITKVSHIPSSRWALVCSLCNEKFGASIQCSVKNCRTAFHVTCAFDRGLEMKT

ILAENDEVKFKSYCPKHSSHRKPEESLGKGAAQENGAPECSPRNPLEPFASLEQNREEAHRVSVRKQKLQ
QLEDEFYTFVNLLDVARALRLPEEVVDFLYQYWKLKRKVNFNKPLITPKKDEEDNLAKREQDVLFRRLQL
FTHLRQDLERVMIDTDTL

Canonical Epc-N
PHD 7e-16

>MgriseaEAA57521
MPSAPSTPQRPTPARRRGRPRTSTSAARSSRVAVADTEPPPKKRRYIPGGPGGGGRFVDENGNEVPRPAG
SSSAPRPRAAAGTPSTGKPRRSLAAGSTAVYPRRERSTRMRTATSRFEPDDEQFSSAAAITSAVIQSEGY
KPREERGWEEFHPNLDIEATFMVFQSEDVDGTARPRPSTPKYAVPAASADTSTPKGASALKTQEDEEATK
DAALVPPEGSQNSTTTPMTRRSRGRPSRDPLGVYGMRGADSTTPKVPKVLPIHNQTPKERLDLKQPSYRK
TDRISLFESKTFGQARYVEKSMMNVGYQESDQYLRPERMLIKATDGNIEEDLEQLSAIAKVDGATTSPAS
NALGRVEYDMDEQDDMWLEKYNERRKEASLDTITREIFEIAITRIEKEWHALEKRIPKPNPKPPQTHRPR
SSSAAAVNGETQAGEEQDSKCAICDDGDCENTNAIVFCDGCDLAVHQECYGVPFIPEGQWLCRKCQLIGR
GVPTCIFCPNTDGAFKQTNSSKWAHLLCSMWIPEVSLGNHTFMEPVMDVEKVPKTRWKLTCYLCNQRMGA
CIQCGNKACYQAFHVTCGRRARLYLKMKNSQGALAVLDGNMVLKAFCDKHCPADYTKENNVAQATRDAKK
FYKKMMKGRLWATSQDSAAELAATHRQALHDQPADDLQATGDNSVAAADKKRGQVQKCWWRLPSGAPVIP
QSVLTIVEGAMQRFPFSKRKDFVAEACRYWTLKREARRGAALLKRLQLQMETFSSMELTRRDFSAMGQSG
KARLERRIEFANMLIKDLEQLKTLSDEIVKRELDKLEAAELEQDFVDTVYFPIYKLLLPVIDKAFALDKS
VFQEGLNGLQNRLDERHYTTTLAFAHDLCEVIHVGINSSTIKPDVVELSDRPEEGEALDVSPSKQGSGYV
EARDRKRLGKRILKAVQPQLETALHAEADIANKPFEKLVKELEGMLEASLELRQASITVSQDENVAPEHN
GEVAMVDAPDVDAGQIIVADHETRTTHHVDAQADVDRMQIDVAVDDAGNIEVDTSGVENAVVEVNGCASS
TSSVAGQENQPTDLTKLDVHLTNGTPKQSGTPPADSNYMHPQLPSGPQPGPLTPPQSNGGLGAGGASGPA
DVLTDGGLPWYLKSFHVDGTSAAEDKWAGRDAVRSLSEDLTDMDEEELNGLEFDVEDSTITASPVNASSS
GPMLSTRKRSISSVRHRKGVRTSARRR

Canonical Epc-N
PHD 1.8e-12

>HsapiensCAE30502
MKRHRPVSSSDSSDESPSTSFTSGSMYRIKSKIPNEHKKPAEVFRKDLISAMKLPDSHHINPDSYYLFAD
TWKEEWEKGVQVPASPDTVPQPSLRIIAEKVKDVLFIRPRKYIHCSSPDTTEPGYINIMELAASVCRYDL
DDMDIFWLQELNEDLAEMGCGPVDENLMEKTVEVLERHCHENMNHAIETEEGLGIEYDEDVICDVCRSPD
SEEGNDMVFCDKCNVCVHQACYGILKVPEGSWLCRSCVLGIYPQCVLCPKKGGALKTTKTGTKWAHVSCA
LWIPEVSIACPERMEPITKISHIPPSRWALVCNLCKLKTGACIQCSIKSCITAFHVTCAFEHGLEMKTIL
DEGDEVKFKSYCLKHSQNRQKLGEAEYPHHRAKEQSQAKSEKTSLRAQKLRELEEEFYSLVRVEDVAAEL
GMPTLAVDFIYNYWKLKRKSNFNKPLFPPKEDEENGLVQPKEESIHTRMRMFMHLRQDLERVRNLCYMIS
RREKLKLSHNKIQEQIFGLQVQLLNQEIDAGLPLTNALENSLFYPPPRITLKLKMPKSTPEDHRNSSTET
DQQPHSPDSSSSVHSIRNMQVPQESLEMRTKSYPRYPLESKNNRLLASLSHSRSEAKESSPAWRTPSSEC
YHGQSLGKPLVLQAALHGQSSIGNGKSQPNSKFAKSNGLEGSWSGNVTQKDSSSEMFCDQEPVFSPHLVS
QGSFRKSTVEHFSRSFKETTNRWVKNTEDLQCYVKPTKNMSPKEQFWGRQVLRRSAGRAPYQENDGYCPD
LELSDSEAESDGNKEKVRVRKDSSDRENPPHDSRRDCHGKSKTHPLSHSSMQR

Canonical Epc-N
PHD 8.7e-17

>NcrassaEAA26920
MAPVPSTPRRRPQSRPKARSRAGGSATTSRGKRHASGAPATEPPLKKRRYVPGGPGGGGRFIDEDGVETP
AEPSGSASRIRTSAARASPSVFPKRERSTRIRTAVNRDKLDDMQYSSAAAVVAAVVQSEGYKPREERGWE
EFHPNLDIEGTFMIFQADEVDGILREAPHTPAVQQQPQQHQLGGQDHGATTPTKSAINNMVSTDAPNAAT
PSGQDRPLLAGSVTDTPSRRRPLRPTRESSSIYANRPLDFGITPKTPKVLPIHNQTPKERLDLKQPSYRK
TDRIALFESKTFGQARYVDKSMSNVGYQESDNFIRPDRKLIKATDANVEEDIDPSTMTQANSDSAHNSAG
AVGRVEYDMDEQDDMWLETLNRRRKDSNLDTITREIFEITITKIEKEWHALEKRIPKPNPKPPQTHRPRS
SSAAAVNGEPQTGEEQDSKCAICDDGDCENTNAIVFCDGCDLAVHQECYGVPFIPEGQWLCRKCQLIGRG
VPTCIFCPNTDGAFKQTNSSKWAHLLCAMWIPEVSLGNHTFMEPVMEVEKVPKNRWKLVCYICNQKMGAC
IQCSNKNCFQAFHVTCARRSRLYLKMKNSQGALAVLEGGLPLKAYCDKHCPQDYAKENDVVQATKDAKRF
YKRAMKGRIWADSQASALQLAATHRHAITEHPPDESQMTGAKVSAVLGETKNGHPGKPIWKLPSGAPIIP

QAVYDLVESALARFPILKRKDFVAEACRYWTLKREARRGAALLKRLQLQMETFSSMELTRRNFAAMGPSG
KTRLARRIEFARTLLKDLEHLKALSEEIVQREASKLEAAEMERDFVDSCYFPVYEMLIPALQKAIQSDRD
VFAKGLSELQDKMEKRYYTTTLQFTHDLCQVISAEINSAMNPHAGLDQIVEPSYASPTKPGTNAEVKKCK
QMGKRILRQVQPYLEAALQAEADICSKPYDTLHQELEGMLDASLEVRQPTITVSSHDGVAAEDVEMADAP
EEGGQIIVADESDGEADGDGDEDGDGKADANVDSNGEPDEMNVDTSQQKAGVGSIEVNTSEVDAAPESNN
TLSTSQSVAGEEEKENHKEDSQQLQLPDGPTKDSGTPPSLPGEGPDRANVPQLSHPQQSGPLTPPQSNGS
FGRDPTTNILTEGGVPTYLHGFQIEGTSVIEEQWPGREAVRSLSEELTDMDDEALKDLEFDVDEADTITA
TSPNHVPVPLGTAEIAVEGGASSSKDVAAAVVTAAVANATATLSAPPVTRRRERANPAMARKGVRSSARR
R

Canonical Epc-N
PHD 1.8e-12

>CfamiliarisXP_538010
MKRHRTLSSSDSSDECPSTSFTSSSMYRSKSKIPHEHKKPAEVFRKDLISAMKLPDSHHISPDSYYVFAD
TWKEEWEKGVQVPVSPDTVPQPSLRVVAEKVKDVLFMRPRKYIHCSSSETTEPGYINIMELAASVCRYDL
DDMDIFWLQELNEDLAEMGYGPVDENLMEKTVEVLERHCHENMNHAIETEEGLGIEYDEDVICDVCRSPD
SEEGNDMVFCDKCNICVHQACYGILKVPEGSWLCRSCVLGIHPQCLLCPKRGGAMKTTRTGTKWAHVSCA
LWIPEVSIACPERMEPITKVSHIPPSRWALVCNLCKLKTGACIQCSVKSCITAFHVTCAFEHSLEMKTIL
DEGDEVKFKSYCLKHSQNRQKLGEAEYPLHRAAEQSQAKNEKTSLRAQKLRELEEEFYSLVRVEDVAAEL
GLPTLAVDFIYNYWKLKRKSNFNKPLFPPKQDEENGLVQPKEESIHTRMRMFMHLRQDLERVRNLCYMIS
RREKLKLSQNKLQEQIFGLQVQLANQEMAAGLPLTSALENSLFNPPPRITLKLKMPKSAPEDCQNSSTEP
DHGPPSPASSSPVHGVRSMQVPQEPLEMRTKSYPRHSLESKSNRLLASLSHPRSETKDPSPAWRTPSPEF
YHGQSLGKPLVLQAALHGQSSIGNGKSQSTSKLAKSNGLEGNWSGDVTQKDSSSETFCDQESILSSHLAS
QGTFRKSTVEQFSRSFKETTNRWPRTMEDLQCYMKPTKNINPKEQLWGKQLVKRSAGRASYQENDGYCPD
LELSDSEAESDGNKEKVRVRRVSSDRENPSHDSRRDCHGKSKTHPVSHSSMQR

Canonical Epc-N
PHD 2.3e-16

>XlaevisAAH72750
MKRVCLPSSSEDSDDNGSLSTSWSQHSRSLPSFRHEDRKPSEVFRTDLITAMKLHDSNQLNPEDYYVLAD
PWRQEWEKGVQVPVNPEFIPETIARVIAEKDKVVTFTRPRKYIHSSGSEPPEVGYVDIQTLADAVCRYDL
NEMDVAWLQLINEEFKEMGMQQLDEYTMEQVLEEFEKKCYDKMNHAIETEEGLGIEYDEDVVCDVCQSPD
GEDGNEMVFCDKCNICVHQACYGILKVPEGSWLCRTCALGVQPKCLLCPKKGGAMKPTRSGTKWVHVSCA
LWIPEVSIGSPEKMEPITKVSHIPSNRWALLCSLCNEKVGACIQCSIKNCRTAFHVTCAFDHGLEMKTIL
TQEDEVKFKSYCPKHGSTKKPEDSHFCRSASDGKDTCEASPTFLGGLRVLEASQQNVKHGSQRKLKLQQL
EDDFYSFVDVHDISQALKIPLDVTEYIYQYWKLRRKANFNEPLITPKKDEEDNLAKKEQDVLIRRLQLFT
HLRQDLERVRNLTYMVTRREKMKRSVCRVQEQIFNLYTKISEQEKDLGFPLENGLLFNTQPSNPDAPKIE
DLKWHSAFFRKRLGSSLRCSMKDSHKKSRERIIGKSLDTEILLTDRKKEGQTSDVSFPLEKTVAKIKPVQ
QKNGGSFPEHRKRRDSRTQGDTKFDSKEKPLRQQHRPAKHTEPPERPAEKKRALSQCGGKSATASSNKKQ
CSSSLPRYSGSLIKIHCNRPSVKVPTSPIKNWGGFRIPKKGEKVQPGSMETCQPNLNCQFLGQVSKKGRT
KEKVKLDNDNDGYTPDAEMSDSESEPTDKCRLQRLTSSSSLSRGYETDFIRRSILAS

Canonical Epc-N
PHD 7e-16

>MmusculusCAE30499
MKRHRPVSSSESSDECPSTSFTSSSMYRKKSKNPKEQKKSAEVFRKDLISAMKIPDSHHVNPDSYYLFTD
TWKEEWEKGVQVPANPDSVPTPSLRIISEKVKEMLFVRPRKYIRCSSPESAEPGYINTLEQAASTCRYDL
DDMDIFWLQELNEDLGEMGYGPIDETLMEKTIEVLERHCHENMNHAIETVEGLGIEYDEDVICDVCRSPD
SEEGNDMVFCDKCNVCVHQACYGILKIPEGSWLCRSCVLGIYPQCVLCPKKGGAMKTTRTGTKWAHVSCA
LWIPEVSIACPERMEPVTKISHIPPSRWALVCNLCKLKTGACIQCSVKSCITAFHVTCAFEHGLEMKTIL
DEGDEVKFKSFCLKHSQNKPKLGDAEYHHHRVAEQSQAKSEKTSLRAQKLRELEEEFYTLVQVEDVAKEM
ELSAFTVDFIYNYWKLKRKSNFNKPLIPPKEEEENGLVQPKEESIHTRMRMFMHLRQDLERVRNLCYMIS
RREKLKLSHTKVQEQIFGLQVQLINEEITEGLSLTNALENSLFYPPPRITLKLKMPKSTSEDCKDSSTET
EHQLSSPGSSSPGHSKRSPQMPEEPLDMNVKIYPRYPLESKSNCLQTSRSHSRCETKSSSPTPRAPSAEF
YHGQSLGKPLALQAALHGQVSIGNGKNQPNSRVSSSNGLEGNWSGNITQKVNSSEVCYDQESMLSSHLPS

PGNIRKSSMEHFSRSFKEATNTWVKPTEDLQYCVKPTKNVSSKEQLWGRQLLRRPTGRASYQETDGYCPD
LEPSDSEAEGEGSKETPRVKRESSDRENPSHDSARECHGKTKTHPHSHSSMQR

Canonical Epc-N
PHD 5.1e-17

>CintestinalisCAE30490
MTSERKYSRRLPSVSCDEVNISGLGSSLNSHTPSRSRRCSEKHDLEANIDENRTDGDDNNNNNRSASAYL
SKLLDCKDLLNDLDASPSPGNPELEANGFTYNSLKPSTPSTSNMRSAIQRSTSLSQKRQSRIPNRHKPAE
IFRKDLISAMKIPDTSQLLNEEYWIMQDPWRIEWEKGVQVPVNAKSVPTKNIQIVPKETLGDFRMPRKML
KSSPDIGDRDMIDLNVLADSTCRYDLDEMDVAWLKIINHERKMMGLATLDEFSMEQIMEELETQCHDNMQ
LAIKTKEGLGIEYDEDVVCDVCRIPDCEEGNEMVFCDGCNLCVHQACYGILKVPVGSWLCKPCALGIRGS
AMCILCNKKGGAMKSTRSGNKWAHVSCALWIPEITIADPDRMEPITKVSHVPSSRWALLCSICKDRVGAC
IQCSVRHCVTAYHVTCAIEDKLDMIADCGVSPLEGQEDAVIFRSYCKKHSTNRKESDGEDEQTEERIRGM
RHRRIMELEQEFYTLVKYQAVANALSIPSQTARDVMEYWKLKRAANFNRPLITPKHEEGTALAEAAETNL
QRRLRMFTHLRQDLERVRNLCYMIG

Canonical Epc-N
PHD 2.1e-10

>GzeaeEAA77830
MAPASPTPRRPVTGRRRGRPPGSTNAARAARAAALAAASATEPPPKRRRYAPAGSRFIAGGAGGGGRYVT
SDILATPNTAGPSTSSRSRAAAREAINGPSPSLMPRRERGARTRAAGNEDLEEMQWGSAAAMATAVKQAE
DYKPREERSWEDFHPNLDIEATFLVLRSEQVDGIPQEQPDMSTVPMLTTPLDDTRTPSRQPNPASTGNTP
NPQGRSDSNAADAPNETPLRRPRRPTRDVVSFYSSRPLDLMTTPKTPKILPIQNQTPKEKLDLKLPSYRK
TNRIELFESKTFGQARYVDKAMSNVGYQESDHYMRPDQSLIKSSDLHAEDDADLTDATASEEPVSHRRIG
RVEYDMDEQDDMWLERLNVRRKEDELEEITREIFEITMTKIEKEWHALEKRIPKPNPKPPQTHRPRSSSA
AAVNGEPQGGEEPDSRCAICDDGDCENTNAIVFCDGCNLAVHQECYGVPFIPEGQWLCRKCMLCGRGVPT
CIFCPNTDGAFKQTNSSKWSHLLCAMWIPEVSLGNHTFMEPVMDVEKVPKSRWKLTCYICRQRMGACIQC
GNKNCYQAFHVTCARRSRLFLKMKTSQGALAVLDGGMVLKAFCDKHCPPDYAQEHNIHQATKAAKKFYKR
TMRNRIWADNTVAANNIAAQHRDALAEQPSEETQLAGNKSSISGDKKKGQPPKNLWKMPSGAPVIPQVVF
EIVEASIQRFPFRKRKDFLSEACRYWTLKRQKRRGAALLKRLQLQMESFSSMELTRRDFAAMGPSGKARL
TRRIEFAEDLVKELEQLRNLASQVVEREQIKVDAGELEQEFVDECYFPVAKLLDPAIDRAISLDKDLFSN
GLDKLQTRINTRFYVTVMSFAVDLCKVISDGVATTPEPKLSTDATQHESNDASPAKHTFSDIRERRKLGK
RILKAVQPFLEAALRVESEISQKPFEGLQKELEDTIDKSVESRRPLTATSQDKSTDPSDEANDTIMVDAE
LQITVKADSTEGGDAMDITSDDGNIDVSTNIDVDTSEIAKAEAGEKQESLPNTVQSSDTPPGTDGYVSKP
QTAQSGPPTPPQSNGSLGLDPSDPLTDGGILWYLKGLDPKGTSVLEEQWAGRDAVRTLSEDLTDLDDEAL
KGLGMDVDNAVASAAVEADEKEEVKAAAEPVGGKTRASKAKKRRASTRRR

AT hook
Canonical Epc-N
PHD 1.6e-14

>DpsedoobscuraEAL30007
MSQRGKRGNQHHHQSHHPPPQQQQRKDVEPQPPPTKRRKGDRVPVIPSSNSKNEVEQELDIGGGGSGLLG
AASSSSSITKSKSTKLAKSSSKSKSQGASTSSSWQARSVADIKMSSIYNRSSTEAPAELYRKDLISAMKL
PDSEPLANYEYLVVADQWKQEWEKGVQVPVNPDSLPEPCVYVLPEPIVSPAHDFKLPKNRYLRITKDEHY
SPELHCLTNVVALAENTCAYDIDPIDEAWLRLYNSDRAQCGAFHINETQFERVIEELEVRCWEQIQVILK
QEEGLGIEFDENVICDVCRSPDSEEANEMVFCDNCNICVHQACYGITAIPSGQWLCRTCSMGITPDCVLC
PNKAGAMKSNKSGKHWAHVSCALWIPEVSIGCVDRMEPITKISSIPQSRWSLVCVLCRKRVGSCIQCSKH
SMSKGKKENAGGASGGGSASVTSSMHKANKYATGTGDGANEGSSACGKTGEDQRRRKNHRKNDMTSEERN
QARAQRLQEVEAEFDKHVNINDISCHLFDVDDDAIVAIYNYWKLKRKSRHNRELIPPKSEDVEMIARKQE
QQDLENHKLVVHLRQDLERVRNLCYMVSRREKLSRSLFKLREQVFYKQLGVLDETRLDKQQQKQDDKQRP
ALDMDAVIYANDGPTLYDRFYSSAGGQTVPAQYQNLEYVLEQLMGKLQSGKQGRGRASQSPNKRKQVAKA
SPTKKLNNGAITSRTSSPEKPAAMGKVPSTSTSTATATTAAKVRGAPPGKPLAGRRASKSGAATGTSTHN
KTQSQSQSHSHIRSSASSHTSSGSSSSGDSSSPNGTSSSDSSSASDSGSESGSSSAASGISRRKSTTGSP
LKKQSYARSVEQRQKQRQRRQSEAAAGASAASPNSRSATSSSEDEDEQQRRRQEPERERDGRGRGAIYNK
TVPNRTQPTKSKQSTQADAGSGAGTGAAVETGAKRKLSTNTRGLAQMHKDAEESLSSDESEELLPLKNER

QREAALSSGLAASGPTTGGRKMGQHIYSDSESSSSSNEKEQEEITAVESNVSDSQNQQTIRTKAAMKEFV
PGTAATTTQSASSATAAASKFTKSTKEAKEGASSCKANSNAKLPYPADLLVVPQRQAAKKASENMRSTNL
AATLQPDAADRMREPESHPATTIAKNKLKDSGSKQATEADKFGGGDKVRSKEQSKSTAKSAGEAIVERGK
RGRPPKVPREPPAPSTSSTEKEKPPPPAEAKSTAPAAKPTAAKTSFAVSYVPQRQAAKKAAEQLKSSKPL
QDTTFSTANESAEKEPAATTATTTGMTTLGVATSSPAKPSRRTSLKEAPNTPKESSANRRKSKEEAAAAA
KTTTPIKRRIAAPNLSSSSSGDSDSSSSSSSSSGSSSSSGSGSDSDSDSQASDAEKPRQAGKEPAPSAAAP
CSTVSSNVPKRSPRKSPLTARTRQNSTNKSPKRVPQKSVATVDIQDDAQSAPKTHSHRRQSSPDEGSKQV
QSEQVTKRATRGSKSRPPSPVVKSSPEKQTARRKSRADESPKKIPNLEHEINQRKAASGKATSALDKMLD
KKQQQINNSTPASPPRKSLTPTPTPPPARSPLPEKLQREPEPVVEPEVETEIEPATEAGELPMDIDEELT
TAPTHTQLSANASKLADIIDDERPPAAPLPASPTPTPTSNDELSDAGSDLSERCRSRWRSRRRRRRRSHE
PDEEHTHHTQHLLNEMEMARELEEERKNELLANASKYSASTSSPAVTVIPPDPPEIIELDSNSANSGGDQ
QQQQQQQPLPQQLMVHSPSSEVASTIQQQQQPPPSHQTLIDQLPVEHMAPVLTTGPAAPCVLEPIVDTIL
EMEDSKFANNFASSLASVLNPPNPGQMSLLGSSLDRGEQISEEDSIQATRNLLEKLRKTKRKAQDDCCSK
EAVDLLPPTPAIPSVFPFHNAADPEDIIHAQKDCLYGNSSGPNSVASLTIKDSPMTANSGSYANSLTNTP
NATPTNATMGNNLGASGGYQVNFGNSQQAPPLSCFLEKSPHQKGGCPLSNNGGAVPGATPDFVDLAAAAV
KNSLGSYQAGAPVTAQSGAGSNSNKLSDYDENTRMQSPFGRMQRWNENDLIAARRSSSPSSVSESNDQPP
ATVAARNISQLEGCKTFFNSYASGNGGGGSAVNPTAPYSHAPLVNGIDGMSMFSNAAAPQQQTTPTHQTP
NSQFNGGIYPQLAVMMHTQSTTTESTPSLYGNGGVGVVPGVGVGVGAVPTTLPLAPPPQAPQYSGTPYTT
PSLGMLPVQQQPVVPPVQVSTTPNHQFALASPVDGKIPAYPAHPAHPAHPAHPAHPAHPAQLLSSCVEAA
VVSMMPPTTPVAVAAKESPNKRSSANSGSAAKKQPNKSPQLPQGKSPGKSPRQPVQPQPPTPPAPAPAVA
LPPSKYDPLTHTIQGKPRQRAPRGSGGSGAPGRGRGRGRGRGRGAGAASGMALPLPPPMSDYGSNTHIVN
NLVGTPFEFNNYDDDITGPGVENLQSLRDRRRSFELRTTRGQPKPTPAPTAATTTNPLLHPVLPGPVDMR
TYNLGFEAPHSTASQEAYQNNLLGAFDSGTADQTLSEFDEEDERQFQSALRATGTGTSPSKHPAVSAAPV
APAPAPAANSQPPANLLLHSTEANQMAPSVAATGGATHLMEGSLVEASLEATSEEVSIDSDTTIMNSKTS
ICDARNQLKLKIKSPLAYSGEHYGAMANSLSSSLSLSSSTLVQSSSAVQTTVSTSTVVSASSVGSGNSRR
MRKKELLSLYVVQKDNLNDDSSCGLPAASDNLPLGPDFLRKSEEEEDVSTGNGNGSKRFKKNSSSRELRA
LDANSVLVEDQQLAGASGGGAGTASGDGRRRSACSSGSNNDNNGKTGAATSAGKRRGRSKTLESSEDDHQ
TPKLKIKIRGLSGSEAVASAGISNAGDSKSYSYEMTRRACPPKKRLTSNYSTPTLEEIKRDSMNYRKKVM
QDFDKGEENNKQDSSGLPLDGEALMPQPPSKRPKSSKPKKDKKEKKRQKQKQLILNSSSATTTMTTTLIE
NTASASPGDKPKLILRINKRKTETSTKITCLEQPPVNEAPLRLKIARNLSGGGYIIGAKAEKKEDPPPDP
PPDPNQPNVSPANELPLMAPLGETSPQGLLLNSFTPHSQNANASPALLGKDSGTPSPPCLVIDSSKSADV
HDSTSLPESGVAAMGVPASLVGATTPLCVNVGNYENSNNSLPSASGTGSASSNSCNSNSNNNNNNGSGGG
AASGGGSLLPLKKDCEVR

Canonical Epc-N
PHD 1.5e-17
AT hooks

>DmelanogasterAAS64921 (rhinoceros)
MSQRGKRGNQQHHQSHHPPPQQHQRKDVEPQPPPTKRRKGRPPNGATTAAVAEVTGSGPATGSERVPVLP
LCKSKHEEPGAEAGGGGQGRAAAGATSTSKSKSTKLAKSASKCKSQGASSSSSWQARSVADIKMSSIYNR
SSTEAPAELYRKDLISAMKLPDSEPLANYEYLIVTDPWKQEWEKGVQVPVNPDSLPEPCVYVLPEPVVSP
AHDFKLPKNRYLRITKDEHYSPDLHYLTNVVALAENTCAYDIDPIDEAWLRLYNSDRAQCGAFPINATQF
ERVIEELEVRCWEQIQVILKLEEGLGIEFDENVICDVCRSPDSEEANEMVFCDNCNICVHQACYGITAIP
SGQWLCRTCSMGIKPDCVLCPNKGGAMKSNKSGKHWAHVSCALWIPEVSIGCVDRMEPITKISSIPQSRW
SLICVLCRKRVGSCIQCSVKPCKTAYHVTCAFQHGLEMRAIIEEGNAEDGVKLRSYCQKHSMSKGKKENA
GSHGGGSASVASAMQKANRYGSGAGGGADDGNNACGTTGEDPRRRKNHRKTELTSEERNQARAQRLQEVE
AEFDKHVNFNDISCHLFDVDDDAIVAIYNYWKLKRKSRHNRELIPPKSEDVEMIARKQEQQDMENHKLVV
HLRQDLERVRNLCYMVSRREKLSRSLFKLREQVFYKQLGVLDEMRLEKQQTKQEQQQPVMDLNAVIYAND
GPTLYDRFYSSVGGQTVPAQYQDLKYILEQLMGKLQSGKQGRGRASQSPNKRKQPAKASPNKKLNNGILS
SRTSSPEKTVAGSKVGTTTSKVRSPPGKNPTGRRASKSSAAAATSTHNKSQFHSNIRSSTTSHSSSGTIS
SGNSSSANGTSSSDSSSGSDSGSESGSSSAGSGVSKRKSSSGSPLKKQSYARSVEQRQKQRQRRQNEAVA
GASATYPDSRSASSSSDGEDERCRNRQEPERGARRGPIQSKSVPNRSQASRSKPTTEADVGEGTGASARR
KLSTTTRGLAQMDKDADESVSSDESEELLPLRGERQRESTTTSGLATTGSAIGRNLGQHIYSDSESSSSE
QEKDQEEQATVESNVSDSQNQQTIRTKAAMKEFVPGTAATTSSTSQAASSTSKAKNTREGKEGAASIGNS
TKTKPNPNAKLYPADLLVVPQRQAAKKASENMRSTNLATTLQPDVSDRVREPDINSISGTAKSKVKDSSS
RVSNEADKSSLEKVRPKEHLQKTVGKTSESAPAERGKRGRPPKVPKDARPPSITENDKPALPTHTQSKPP
SVVATPVSAKSNFAVSLVPQRQAAKKAAEQLKSSKPVLESFSTGNDISDKETVTSATISGSGSSVPAAST

PVKPTRRSSIKEAPITPKEPLSGRRKSKEDLLATPIKTTPLVKRRVVVPNLSSSSSGDSESSSSSSSSGS
SSSSGGSDSDSESQASNSENPSSREPPVAPAKVPSDSSLVPKRSPRKSMDKPSALTIAPASVNVLNIPST
RSRQNSTTKSTKVALQKAVQSVEDDVKCTPKTNRLQGSMDECGKQVQLEQATKRATRGSKSRPPSPTAKS
SPEKTVSRCKSRAEESPKKVANLEQEISQRKVASGKGTSSLDKLLNKKQQQMNHSAQATPPPISPTPPAS
ETRIVKDQCDLKPDEVSIQQINLGADAQPEPDLDPESAAEAGELPMDIDEELTTAPTRTQLSASASKLAD
IIDDERPPAAPLPASPTPTPTSNDEMSDAGSDLSERRRMRWRSRRRRRRRSHEPDEEHTHHTQHLLNEME
MARELEEERKNELLANASKYSASTSSPAVTVIPPDPPEIIELDSNSAEQQQQHLHDQPLPPPLVVQSPAA
DVVPTVMQQQLLPSQRPLIEQLPVEHLPIVETILEMEDSKFANNFASNLASVLNPPNQMSLIGSSIDRSK
QISEEDSIQATRNLLEKLRKTKRKAQDDCSSKEAVDLLPPTPAIPSVFPFHNAADPEDIIHAQKEQQHQQ
QQQLQSSQTCIYGNSSGPNSVASLTIKDSPMTANSGSYANSLTNTPNATPTNATMNNLGYQVNFPNSQPP
PTLGLFLEKSPHQKGACPLSSNGGANVGQPAPTPDFVDLAAAAVKNTLGSFRGAATVPTQSGTGVNAKIN
DYDESTRMQSPFGGMPWNESDLIAERRSSSPSSVSESNDPPQPPPVVTATATTARSLAQLESCKNFFNSY
PSGNAGPGTAANATAPFNHPPMVNGIDSIPMFNNTNTTQHQPTTPAHQQQQQRTPNNQYNGTIYPQLAGI
MHPQTTPTEPPSSLYGNGGVGGAVQSTTLPPPAQVNQYPGTPYSATTLGMISVQQPALSTVPVQTATTPN
NPFTLTSPIDGKMPTYPAQLLSSCAEAVVASMMPPTPPVTATAKDSPSKRTSVSGSNLSKKQTHKSPQLP
QGKSPGKSPRQPLQPPTPPAPVPVVALPPTKYDPQTHTLQGKPRQRAPRGSGGSGAPGRGRGRGRGRGRG
GGVTSGMAMVLPPPMSDYGSNTHIVNNLVGTPFEFNNEFDDMAGPGVENLQSLRDRRRSFELRAPRVQNK
PTTTPTTATTTNPLLHPVLPGPVDMRTYNLGFEAPHSTASQEAYQNNLLGAFDSGTADQTLSEFNEEDER
QFQSALRATGTGTSPSKQHSGPTALVAPPTGPNPTPAPNLLLHCTEANQMAPNVAATGAATHLVEGSLVE
ASLEATSEEVSIDSDSTIPHSKTSTSDARSQIKLKIKSPMAYPEHYNAMTNSSSLTLTSTLVQSSNVVQT
TVSTSTVVSASSAVSGNSRRMRKKELLSLYVVQKDNHNDDSSCGLPAASDTLPLENLRKSEEEDELSGGN
GTKRFKKNSSSRELRALDANLALVEEQLLSSGAGACGGGSSGDGRRRSACSSGSNNDNNGKTGAASSAGK
RRGRSKTLESSEDDHQAPKLKIKIRGLTANETPSGVSSVDEGQNYSYEMTRRACPPKKRLTSNFSTLTLE
EIKRDSMNYRKKVMQDFVKGEDSNKRGVVVKDGESLIMPQPPTKRPKSSKPKKEKKEKKRQKQQQLILSS
STTTMTTTLIENTASASPGDKPKLILRFGKRKAETTTRTASLEQPPTLEAPAPLRFKIARNSSGGGYIIG
TKAEKKDESTADNTSPITELPLISPLREASPQGRLLNSFTPHSQNANTSPALLGKDTGTPSPPCLVIDSS
KSADVHDSTSLPESGEAAMGVQSSLVNATTPLCVNVGNYENSNNSLPSASGTGSASSNSCNSNSINNNGS
GGGRASGEGGLLPLKKDCEVR

Canonical Epc-N
PHD 1.5e-17
AT hooks

>CfamiliarisXP_538629
MSVGGGLRRPVPEEASLRICVYKIIFVMAITVPGKALKLMRDRKEWGSDPRLKRFKSVQLLFSECLAFPT
QGGSEDRGNLALGEIEGIGGKRKPEVKTQLSVPTNPTWISALVKESSESPSSMEEKRRKYSISSDNSDTT
DSHATSASRCSKLPSSTKSGWPRQNEKKPSEVGSDKCLGKAEAVVGKPQEWSPVETKGVFRTDLITAMKI
PDSCQLSPDDYYILADPWRQEWEKGVQVPAGAEAIPEPVVRILPPLEGPPTQVSPSSSELGEGSQPDWPG
GSRYDLDEIDAYWLELINSELKEMERPELDELTLERVLEELETLCHQNMVRAIETQEGLGIEYDEDVVCD
VCRSPEGEDGNEMVFCDKCNVCVHQVWGIPGPYLQKTCLEPSGPTLALTLSTYNNSLASAAGIALGEPRG
ASPPVSQACYGILKVPTGSWLCRTCALGVQPKCLLCPKRGGALKPTRSGTKWVHVSCALWIPEVSIGCPE
KMEPITKISHIPASRWALSCNLCKECTGTCIQCSMPSCVTAFHVTCAFDHGLEMRTILADNDEVKFKSFC
QEHSDGGPRGEPTSDPVEPSPASEDLEKVTVRKQRLQQLEEDFYELVEPAEVAERLELAEALVDFIYQYW
KLKRKANANQPLLTPKTDEVDNLAQQEQDVLYRRLKLFTHLRQDLEREEEAGPTFQGVGGPCQHAAFPAA
PELDWQAPAESKARERAALAGGGEETTVLAVGAAQGLVPKALGLVTRSLLGSMTWLERVGIALRNTALSL
KIDLWISPRPCVAANPLGPPPPLKPGPTSPAASSVTCNLKCPSSPTASRLPPRSSRRLPAHVAVLVPIPQ
PRFLFLGPDPSNFWAVKPVKPVEHLYQFSALSPPAPRTHCAHGNIKRSVRNLCYMVTRRERTKHAICKLQ
EQIFHLQMKLIEQDLCREGSGRRAKGKKSDSKRKGREGPKGSPEKKEKMKAGPDSVLGQLGLSTSFPIDG
TFFNSWLAQSVQITAENMAMSEWSLNNGHREDPAPGLLSEELLQDEETLLSFMRDPSLRPGDPARKARGR
SRLPAKKKPPQDGPGSRTTPDKSPKKPWGQEAGKAGQGPVARKPTRRTPSHLPSSPAAGDCPVPVAPESP
PPLAPETPDETAPVAADSNVQVPGPTVSPKPLGRLRLPRDKGTQRSPGARPDVGTGPPSTVAERPKVSLH
FDTETDGYFSDGEMSDSDVEAEDSGVQRAPREAGAEEVVRMGVLAS

Canonical Epc-N
PHD 1.3e-06

>MmusculusCAE30498
MEEKRRKYSISSDNSDTTDGHVTSTSASRCSKLPSSTKSGWPRQNEKKPSEVFRTDLITAMKIPDSYQLS

PDDYYILADPWRQEWEKGVQVPAGAEAIPEPVVSWAARGGQAKASLFITTILFPRLLPPLKGPPTQMSPD
SPTLGEGAHPDWPGGSRYDLDEIDAYWLELLNSELKEMEKPELDELTLERVLEELETLCHQNMAQAIETQ
EGLGIEYDEDVVCDVCRSPEGEDGNEMVFCDKCNVCVHQACYGILKVPTGSWLCRTCALGVQPKCLLCPK
RGGALKPTRSGTKWVHVSCALWIPEVSIGCPEKMEPITKISHIPASRWALSCSLCKECTGTCIQCSMPSC
ITAFHVTCAFDRGLEMRTILADNDEVKFKSLCQEHSDGGPRSEPTSEPVEPSQAVEDLEKVTLRKQRLQQ
LEENFYELVEPAEVAERLDLAEALVDFIYQYWKLKRRANANQPLLTPKTDEVDNLAQQEQDVLYRRLKLF
THLRQDLERVRNLCYMVTRRERTKHTICKLQEQIFHLQMKLIEQDLCRGLSTSFPIDGTFFNSWLAQSVQ
ITAEDMAMSEWSLNSGHREDPAPGLLSEELLQDEETLLSFMRDPSLRPGDPARKARGRTRLPAKKKPSPL
QDGPSARTTPDKQPKKAWAQDGKGTQGPPMRKPPRRTSSHLPSSPAAGDCPVPATLESPPPLASEILDKT
APMASDLNVQVPGPTVSPKPLGRLRPPREMKVSRKSPGARSDAGTGLPSAVAERPKVSLHFDTEADGYFS
DEEMSDSEVEAEDSGVQRASREAGAEEVVRMGVLAS

Canonical Epc-N
PHD 1.3e-15

>RnorvegicusXP_220398
MEEKRRKYSISSDNSDTTDGHVTSTSASRCSKLPSSTKSGWPRQNEKKPSEVFRTDLITAMKIPDSYQLS
PDDYYILADPWRQEWEKGVQVPAGAEAIPEPVVRILPPLKGPPTLMSPESPTLGEGAHPDWPGGSRYDLD
EIDAYWLELLNSELKEMEKPELDELTLERVLEELETLCHQNMAQAIETQEGLGIEYDEDVVCDVCRSPEG
EDGNEMVFCDKCNVCVHQACYGILKVPTGSWLCRTCALGVQPKCLLCPKRGGALKPTRSGTKWVHVSCAL
WIPEVSIGCPEKMEPITKISHIPASRWALSCSLCKECTGTCIQCSTPSCLTAFHVTCAFDRGLEMRTILA
DNDEVKFKSLCQEHSDGGPRSEPSSEPVEPSQAVEDLEKVTLRKQRLQQLEENFYELVEPAEVAERLDLA
EALVDFIYQYWKLKRRANANQPLLTPKTDEVDNLAQQEQDVLYRRLKLFTHLRQDLERVRNLCYMVTRRE
RTKHTICKLQEQIFHLQMKLIEQDLCREPSGRRSKGKKNDSKRKGREGPKGSSPEKKEKMKAGPESVLGQ
LAGLSTSFPIDGTFFNSWLAQSVQITAEDMAMSEWSLNSGHREDPAPGLLSEELLQDEETLLSFMRDPSL
RPGDPARKARGRTRLPAKKKPPPLQDGPSARTTPEKPAKKAWAQDGKGTQGPPARKPPRRTSSHLPSSPA
AGDCPVPATLESPPTLASEILDETVPAASDLNVQVPGPTVSPKPLGRLRPPREMKVSRKSPGARSDAGTG
LPSTVAERPKVSLHFDTEADGYFSDEEMSDSDVEAENRGVQRASREAGAEEVVRMGVLAS

Canonical Epc-N
PHD 1.3e-15

>HsapiensCAE30501
MEEKRRKYSISSDNSDTTDSHATSTSASRCSKLPSSTKSGWPRQNEKKPSEVFRTDLITAMKIPDSYQLS
PDDYYILADPWRQEWEKGVQVPAGAEAIPEPVVRILPPLEGPPAQASPSSTMLGEGSQPDWPGGSRYDLD
EIDAYWLELINSELKEMERPELDELTLERVLEELETLCHQNMARAIETQEGLGIEYDEDVVCDVCRSPEG
EDGNEMVFCDKCNVCVHQACYGILKVPTGSWLCRTCALGVQPKCLLCPKRGGALKPTRSGTKWVHVSCAL
WIPEVSIGCPEKMEPITKISHIPASRWALSCSLCKECTGTCIQCSMPSCVTAFHVTCAFDHGLEMRTILA
DNDEVKFKSFCQEHSDGGPRNEPTSEPTEPSQAGEDLEKVTLRKQRLQQLEEDFYELVEPAEVAERLDLA
EALVDFIYQYWKLKRKANANQPLLTPKTDEVDNLAQQEQDVLYRRLKLFTHLRQDLERVRNLCYMVTRRE
RTKHAICKLQEQIFHLQMKLIEQDLCRGLSTSFPIDGTFFNSWLAQSVQITAENMAMSEWPLNNGHREDP
APGLLSEELLQDEETLLSFMRDPSLRPGDPARKARGRTRLPAKKKPPPPPPQDGPGSRTTPDKAPKKTWG
QDAGSGKGGQGPPTRKPPRRTSSHLPSSPAAGDCPILATPESPPPLAPETPDEAASVAADSDVQVPGPAA
SPKPLGRLRPPRESKVTRRLPGARPDAGMGPPSAVAERPKVSLHFDTETDGYFSDGEMSDSDVEAEDGGV
QRGPREAGAEEVVRMGVLAS

Canonical Epc-N
PHD 1.3e-15

>ScerevisiaeCAA95027
MNRGSLDDGPKLREEKHFQDFYPDLNADTLLPFIVPLVETKDNSTDTDSDDISNRNNREIGSVKSVQTKE
LIFKGRVTTEPLVLKKNEVEFQKCKITTNELKGKKNPYCVRFNESFISRYYHINKVRNRKSYKQQQKEFD
GVEAPYFTKFSSKEAPNITISTSTKSAIQKFASISPNLVNFKPQYDMDEQDELYLHYLNKRYFKDQMSHE
IFEILMTTLETEWFHIEKHIPSTNSLIARHNILRDCKNYELYGSDDGTGLSMDQACAVCLGTDSDNLNTI
VFCDGCDIAVHQECYGIIFIPEGKWLCRRCMISKNNFATCLMCPSHTGAFKQTDTGSWVHNICALWLPEL
YFSNLHYMEPIEGVQNVSVSRWKLNCYICKKKMGACIQCFQRNCFTAYHVTCARRAGLYMSKGKCTIQEL
ASNQFSQKYSVESFCHKHAPRGWQTSIEGINKARKYFSLLSTLQTETPQHNEANDRTNSKFNKTIWKTPN
QTPVAPHVFAEILQKVVDFFGLANPPAGAFDICKYWSMKRELTGGTPLTACFENNSLGSLTEEQVQTRID

FANDQLEDLYRLKELTTLVKKRTQASNSLSRSRKKVFDIVKSPQKYLLKINVLDIFIKSEQFKALERLVT
EPKLLVILEKCKHCDFDTVQIFKEEIMHFFEVLETLPGASRILQTVSSKAKEQVTNLIGLIEHVDIKKLL
SRDFIINDDKIEERPWSGPVIMEEEGLSDAEELSAGEHRMLKLILNSG

Canonical Epc-N
PHD 1.9e-17

>TnigroviridisCAG08803
MAHILARPQHRALSLLLGSTCESTGSPVGDQSALIPTSGTSTFYPAGTSFQLLCERGEEFSPFFPPLLGP
LTQSRYWQRNHPSVYRCVFLNRINGGTNISENFSKFSKTDSNRADVGRERRRAEAWSQRQPVSGAKASAN
ANCSGAEELLPALASEAAGRPERRSSGRPAPFCCYDLDDLDVAWLELVNHEFRQMALPELDELTMECVLV
ELESACEEKMRQAIETEEGLGIEYDEDVVCDVCRSPEGEDGNEMVFCDKCNVCVHQACYGILKVPRGNWL
CRTCALGVQPKCLLCPKRGGALKPTRSGTKWVHVSCALWIPEVSIGCPEKMEPITKVSHIPASRWALSCS
LCREHTGTCIQCSMPSCIVAFHVTCAFDHSLEMRTILAENDEVRFKSFCLEHSCTASNSAPGLAGVSNGN
HGLPVTKGAQASAHDQASAAGCMALRPDGEQRSLSAAERDQLEREKVSLRKQKLQELEDEFYRLVDPREV
ADNLELPVFQVDFLYQFWKLKRKSNFNRPLVTLKRDEVDNLAQQEQDVLYRRLKLFTHLRQDLERMKLRH
EVIICPPGCESPPVTIAPTRAAGFCLGPVEVRNLCYMVTRREKMKHTLCDLQEKIFHLQIQLLEEDIAGG
ETETNETPQLWLHGSSDGLRVCERKEEKVRVKDEKGQKEKSQRGKRKQNGVKERGKERRKSSPQKKKEKL
SENGSLLKELGLSKSFPLDKSLFDSWLAQSVQITADDMLSQWTLGAQHRDKSGSLLSDQLLQGEESLLNL
MMENSMQSTWKTPHLGRKQRGSSKSRPRGPSQSPSPGLPPPLLSAAATSAASRPSSTKNLWVNVQEKFSH
LGQRGERSRSSLKALHHHFHQHQSRNSDLRRDPPPQPPISKMDSCHISEDHDRWESVHSGGGVAAGTRSG
FSTSSSLAPASSPGLDTGTLLQSSVPRLRVSRQAKSRGRSFGCVEDLEAVRLPLPEKDRLEAAETDGYFS
DGEQSDVDTRSGKRKLRLPPLHSRERGSAEKERASVLKHRDSGHALKSPCGGSKHTPLWSRFIAHLLSSW
DDYECSCLPGVRLKPSTGPESPPLASHTRTSSALFALMRQIGSPPGIGRTRPWRYRPGSGDKHFWALLFL
L

Canonical Epc-N
PHD 8.7e-16

>AgossypiiAAS51419
MSSGQTPVRAQEEGPKLREEKHYRDFYPDLNHSVLLPVFTASGEETALQDIEYKARINALRQLSRASGSL
KQIIYKNKVTVERLGPQLKKTCFKPCKVKVTQLNGKYHVPAVFHRYGYRSNVQTLNVDLARRAYMKKTDD
LLRNENEFFVDVSRYLRDFKVQYDMDEQDDLYLQYLNSGKARGSANALSAELFEILITALEIEWFYLERK
IPQRHPTNQQSSTHESEAAIAHYELYGSDDGSGSSADQSCAICNGTDSDNSNAIVFCDGCDVAVHQECYG
VVFIPEGQWLCRRCMISKNRKINCLFCPSNTGAFKQTDTGSWGHVICGIWIPELFFANQHYMEPIEGIDM
VPRSRWKLNCYICKQKCGACIQCSNKNCFVAYHVTCAKRAGLFMTFGGCTVPEAASKNFRPGVKLESFCD
KHSPSGWGDCQVGILKTRRYFENIKEMVMRGNQRATSSEAQQPPTRNRWKTNRGTPIAPQLFATILKQLL
DKFGIAEAEQTAIDICKYWSMKRELKRGAPLVRIFDPTSFNSMDSADILKRVAFADVLLNDLAKLDELST
LLVRRQQAAQARLDAVDIINDLGFHPVRHLVQKNVTTHWSTKEFIALMNFEPAFAGVLAKVDSDQYDSIA
SFSREVRTLFDQIAKEKDIPVELVSTITVCQRQFSKQIAKIEGLDVHKLMSRDFIFEGKKIREVDWHGPI
LMKEEELSEVEEDELTPAQERILKSFLRY

Canonical Epc-N
PHD 2.3e-17

>AmelliferaXP_396822 (misannotation?)
MAQRGKRINRNDNDLASCPGAIKRRKCRLGPATGSSSLAATMGPSEEEETMASSSQGGASWTPRPLCDIK
ISSIYNRSSAEAPAELFRKDLISAMKLPDSEPLSPNEYWVITDQWKQEWERGVQVPVNPDSLPEPTVTIT
QATPIKQHSEFKLPKKFVRISRDDYFNPEDHHLSTTPARAEKACAYDLDDTDIAWLDVLNGERAQAGQLP
ITESQLERVIEELEVRCWERIQTIVKNEEGLGIEYDENVICDVCRSPDSEEGNEMVFCDCCNICVHQACY
GITSIPDEVSIGCVERMEPITKISSIPQSRWALICVLCRERVGACIQCSIKTCKTAYHVTCAFKYGLEMK
AIIEDEMADDGVKLRSYCQKHSRTNTKDKVQGTGSSIGSGGDKAGSDSEDAESRRRKRKDMTSEEKNQAR
AAKLQEIEAEFDKHVSLKDIASQQLDVDPDGIVYIYNYWKLKRRAGHNKPLLAPRCGELSGSGSRNQGQA
ADLEKMRTFVQLRQDLERVRNLCYMVGRREKLCRSFLRLREQTFHKQALVMSGPPLPPAAAAAVMEANHG
PSIYDRLYSHPDSEDHTHDFDTIVARIDFNGASSKKLYFNGSVRRKNLYGSDLSSVSSSETDATKAKTEK
SKNLKLETESSTEEETNVSAKTKLSRRKAKKAVQSVEKLHSTVRENSENEKLVNSRSHTLEHMEKELGSA
SGSESDELLTLSGGATKHFVASAIYSDTDSDSQEHASHSAVLITKAAVKEFSAANLSKNSQKNFNKDSRH
VESTYHNTGSKNKENQNKTSVKKKEYIPSALIVPQRQAAKKASEIMQRTQQGKKDNSVPETMSEIIKSPV

EEIPKSSSSKQSKDKSPNKKQRENKQSKEIKNNDVYDFDKEFGDGTEILAYVPQRQAAKKAAEHIKSGMG
NKTTQQIEQETFDGRSKKESPETGKRKEFRKDDSSRKEELSSRKEEISSSSSSSSSSSSSSSSSSSSSSS
SSSSSRKEDLSSRKEELVSKKEESSRKEEISKESRSRRPRKSNELIDGSSATTSSESDSGNRTKSKAIPS
SANRKQQPSKTSPERQEAERKSISGRKLKKLPEKKLKTSDGRRGPEFQPPTEREEPRRACKEQQQQHQT
QQEASVKKQDQRDKKQSTTTAGTEVDEDLLIGSGDNDGKLHLGKKKTDRNLGKSQENAIEHQQSVELQNI
ESQTISLGENVQKESNEDDNGKSILERVENSEKEHQKRRKSANRSIFSPQHGKDASVSELFDFDQDMLTE
EIVNEDGFGISRDAEERGVPLSFSFNNELWFKEDSKEDSARETLHLVEKLRMELSKKSNSSQIELEAVPV
DEPVLNVKNIEISEEEISTNETRPSSCKSTIEEKRKSCYSGGNDRYAAYEEDRETNKVSLVERSKLDRAE
TDERWVPPSINSFQLSDVQHLNALMETQNRRYGNHQFPNESIPENESIARTHLNAPITAPPTESIERTQQ
QLQTQVADSPSLPMNFLNTVAPENHTNDTVVESLQEALADGKSQHSGKKSPSKPTRTSARVTSLQGKSPG
KSPRQDTQKTIPGARGRGRGTKNSAQHSGYRGRGRGRGRGRGRSGQNSGLSLVSGGGIGQHDDSIQNKLV
GTVYDFDSDEDSTSEANIADLRTMRERKKSTDAATAGVERRDSTVMASGESIQSRLSSPGGTRKYLEDRR
LSCSPNHDDSAVVENQSNAGQSFDTVLPLIPGPVDMRTYNSTLDASSSSTLHGQTYENHFLGTFTGVSTN
DPALPDIEEDLEKELRSALIKQGQEDCSKPSFDASIDASSSGFVDTNKVSLTDSRNQLKVKIKGPFLDAN
YVATSSVPPLAQQAPVIITPAATSASIASGTSNLRRMRKKELICLIVTCNKMKPTLVADEMFPEGAGSYI
ELEEVGGSRLLVDLTASEKAVHADFFNDFDDLYDDDDLE

Canonical Epc-N
PHD 1.8e-09

>KlactisCAG99327
MQTQEDGVRLREEVSFKHFYPDLDVNDLVLFGKNNHDELNDRHRHDEEEVYPDDNLREHLKSWKQLIVND
SITIEMLGPQIKNAEFKRCKIRISQLNPHGKVNKQLVKYGYRSHADRTCGLDQKSYCKKTDLLMKHSNEF
FADVSPYQVNFKVEYDMDEQDDLFLRHLNTALTNSKHKLSHEVFEIVITILENEAFHLEKKIPPRVSPSA
TNNTHESHAAWKHHELYGSDDGTGYPMDQPCAVCGGIECDNSNAIVFCDGCDIAVHQECYGVVFIPEGQW
LCRRCMISKNRKLECLFCPSTTGAFKQTDNGSWGHVLCGIWIPELYFGNLHYMEPIGGIENIPKSRWKLT
CYICKQEVGACIQCSNKNCFAAYHTTCAKRAGLYMNFNGCTVQEAASKNFSTGAFLESFCHKHSPNGWSS
CEDGIRKTRDYFNHINHHKVLQDSAVEENKQSAKKTIKNKWKTSRGTLIAPQAFADITMRVLNRFHVPNS
EQISLDICKYWTMKRELKRGAPLVRKFSPSSLNTLDTEELNDRIVFSEVLLRDLNSLEGLASSLVAKQEC
NVERVRNQRIINDIAFHPIRYILKDVVWNNLTKTLSFKSFADLESSSNFKVIPYISSRLENDEYLSVQEF
KDEFVGNIVKITSNERSPRSLVIMGSKLINDLNKLLSKIDELDVHTLLHDDFDIDERTHQVEEVNWKGRK
ILAREGLADVEDLSIKQERQLKGLLRD

Canonical Epc-N
PHD 3.1e-14

>CalbicansEAL04413
MNSVSANSYDFSLKPREETDFKEIYPDLDETCQLPVFVIDRNTNSEESSNLRHAVNLSELKAPTYRRVPQ
LSLTPTNLRFSKQLSEYGFQNPSKLTNKPNPDTYIRPFQLNASLRETTASIEKFIEKKKNLVEYDMDEQD
YLFLQDRNNQPENVIKITPEVFEIMMTSLENQWCALELKMNSITTNVGSSTSDPGSNHKLLTIGHNNAKY
GNDDGIVPGSIYDQKCAICNDSDCDNANAIVFCDGCDIAVHQECYGVAFIPEGQWLCRKCMINKNRTTEC
VFCPSTTGAFKQLDNSLWSHVICGLWINELYFANPIYMEPIEGMEGIPKSRWKLTCYICKQRVGACIQCC
NRSCFQAYHVTCAKRAGLYMSMTQGIKGAISNKLTLKSYCERHSPAEFDETKVLDGIRRTRLYYRDTKLL
NEENARLSKDRETANKLNIFKWNTEANTPIAPKLFSDVLVQILYQLKVENQISLPEESTNEVLHLKVLPN
RTKMEILQDLRSIADEVCRYWCLKRKSKRGASLIRKNNNLIATSSILYDSNSLENNNACESTKKIEFAES
LVSDIDRLISMGEMLTDEQEANHEINSVSFDVVDSVYFPLKQLIELTIMRLNNKFDFNETLRTYKPKSDA
VISLNSISGKNSQYGYQSIDQLEQDVENLREYIFQENKPSSAVYKKMKNWIAGFRKELPSLQVFEKDVIN
DGNQKSSLSFPNVRSAGTNTDLKLVDYRTILEENDLSEIDDDEIESVQNQKELEKFLQD

Canonical Epc-N
PHD 2.3e-14

>CbriggsaeCAE69485
MTPGSSEGPSFGSPGIIRRPPSEKRPRRNGYETFPTVQKPIDRRKSDVKHKTSKRPAPPPESDNDDVPST
SSAPVKPKKKKEKQEPRYDTSTSTIPFTQMASRYDMDADSDTELADCLPFAYIGDKWRTEYSRGVPIPCE
TTLPRAVVMDDGFVLKPKDDYRHPVKRIACSDRAEYFDEERFELLEGEEEEKVHYEVTAHDLKWLERLNK
ARKQSNGKTYLPTTVFSKIMEILETQTYTAIHKQLLNSLHVCVSSPRDDDECDVCRDVDTDGSEEMIYCD
SCNICVHETCGGVKTVPTGGWKCLKCRFSRQGPAPKCIFCPALGGSMTHSADKKLWAHHSCALFVKQIEF

EDAEDRAPIKFVEKVEEHQYREKCCVCDTKQGVCVKCSDEECEMTFHVCCALRAGCQVVVKEKLDHSGMD
YIHKCHRHSEPENNRRLAIEDEWLEYRNPWLAKLESFFYGFVNYVDVSISTSLPEFLIADAFEYWKQKRL
DAGGPLIRNLSDLIPVPTTIEDVARRAMETQQNRASISIGTVGMALKPSTSSDPSGPSTSTATTSSGPET
SGSGLRGAEHQENHPFFRPAVLMVNELRHIHLKRAANAMKRDIMLSKMVMRREGLNVKLAEASLETIRIA
QMMFETGEESLEEIQKAFDFESMSKNQLKRAAEPERFKAFLERIGGVQRPQKAPRTSGDHQNAQKATQKT
SGGFQKQQLPKTSPEKSPLRRQNHNSSPSKPRSSEAVRPGTSGTPFRPTSSASDAQNRPTSSGTSSIAAS
PDHPHNTRHKHNRITTPWNYDQNHNNNNKSMTSSGSHHRRSTSSKNQDFLASPASGPSTSSRFH

Canonical Epc-N
PHD 3.8e-09

>CglabrataCAG61415
MSRASNDVGPKLREEKHFNDFYPDLDANDLIPILVSQETDQSQSVDSQDEIIRKQHYKQLIFNGSITVEP
ITITKQATDVQQIPYPVNDFATFIDAKRYSKKSVGNTLISKFYSQDHIQGKTKRRQQRIINAHDSDKIYK
DKFMTHSNQIKRDKAIQSHFEALSPNLSGFIPQYDMDEQDALYLEFLNQSLGEKYITAEVFEIIITILEL
EWYHLDKHIPPKITDTNDSQLNSIQHHANKVRYELYGSDDGTGLTSDQACAVCDGTVSTTTNMIVFCDGC
DIAVHQECYGIVFIPEGQWLCRRCFISRNKQVNCVTCPSTTGAFKQTHTGSWAHVLCALWIPELVFANLH
YMEPIEGVENINKSRWKLVCYICKLRVGACIQCSNKNCFAAYHVTCAKRAGLCLDTHDTSIAEMASKHYQ
MHHHVTSYCDKHSPPGWPSCAEGIMKTRRYFANRDAISEVSKEKQLITKGVNSEDKNSAHWKTNKGTPIA
PMYFTHIIQKVLVMFDISNEIPLSILLCKYWAMKRELRKGAPLVRVHTNLSYNSQNESYLNDRIQFIDSL
LGDLQSLGNISRLIKERVNATKSFRATNKKINEVFTSPKEYFFRRNVLSKFLHFNAFTSLLALLDKHTIV
DHSVNALRNSTSATFIENVEAFVNFASTKLDETRLTSDYIHRISNYAKELSAQFDKVDYEREINMDFEVN
KEHDKLSVSERLWKGPILQQEEGLSDVEELTPSESRIVNNLPIEKEKPTKVKRRKKWKKSY

Canonical Epc-N
PHD 1.1e-12

>SpombeCAA21075
MQTFRLTSTGRNILRPDELAFQPREEIPYKSFHPDLQIDEPLEILEGDHTQYAGLRDSLVTYKSENSYVL
KALLNAKIENVKPVGVQTENINPQEKKFGYKTAKQLDWSPDEYFKFVAIHPYSKTSFPVSYDLDELDTMW
LTYYNEFQLSSNSEWENVSKEFLEIVLTIIEREWLYLEAWMPKIEPVRVEDELDGRCVICNEAECENSNA
IVFCDNCNTSVHQNCYGIPFVPEGQWFCKKCLLAPHEVICCAFCPDRDGAFCTTLDGRWCHTICAIAIPE
ISFHDTSRLDLVRNIASIPKSRWKLVCCICKLRWGTCVQCSDKNCYAAYHITCARRAGFFYKIYSHSASY
DSVDMETYCDKHTPPDYLNGLMKRLFPLAELYYKRMATDVPLNFQATKAPDFVPEGPWKSHPLPAFIVDK
VTKVLLSYNVKRQDLPSIVTDICKFYHMKRRSRKDAPLLKSQLLMDSLENLPVRASKDRVRSLEVAKALQ
DQYQSLLTLVESTAKRQLLKCQLSNLRKKFLNLNYFPAQRLLQDTLVKIIDLDVDGLFNMPLDNGWIGWV
ELKRQVFSYQIGSISSLEKKLEPIWDVDGVIQCIDDMEQLTAMVQFAQKTEGEVKKLFIKAKIYFESLSL
DERGNLKVPSLGINGLEYDNWPGLNELEMSQLDIPSQGNLKSLHDFIEGLDLNEKIGKFPISMFQNQVAQ
FSTIEIPKMSGRANGMHNFHSEDVTGQSNHALPNSVTKKNGTKQPYTKNSLPFNERITRSKAKKNYS

Canonical Epc-N
PHD 1.6e-15

>EcunuculiCAD27158
MYRKKKFSIPCGESWNPKMSTSGWLGGIPREEHSYKAIYRNLELSRMHRVVSDHVPMSEEKHEGKKTIDE
EQPRGLFESVIYRLDGYDQRYLRESAIDVSEDAFELIVDRLEKEWFFFVHGLVDRHVKPIEPSSFCDICT
KHTSTHNEALVVCQGCEICVHESCYGIQDLSSFWLCRKCIYGEYQIRCSFCISSDGIFKQTSDNRWGHVL
CAMFNRFLSFGHLLSKDPIDVSSYLEESGCLFCNEFGGTAIHCSYFMCTRKYHVGCALDKCYFDLNNGIS
YCIDHDPLKRNPYELGYGRMNGLRYFGYEKLRNPPAIRRKVRMARPRATLFMKLCKLQPVATPSVLSRVE
ACDLKEKDSVNIFKVSRYWELKRKEIGGPLVILPDIVYGRKTREDWMSKRRM

Canonical Epc-N
PHD 8.1e-07
